# Diversity in the Architecture of ATLs, a Family of Plant Ubiquitin-Ligases, Leads to Recognition and Targeting of Substrates in Different Cellular Environments

**DOI:** 10.1371/journal.pone.0023934

**Published:** 2011-08-24

**Authors:** Victor Aguilar-Hernández, Laura Aguilar-Henonin, Plinio Guzmán

**Affiliations:** Departamento de Ingeniería Genética de Plantas, Centro de Investigación y de Estudios Avanzados, Unidad Irapuato, Irapuato, México; Kyushu Institute of Technology, Japan

## Abstract

Ubiquitin-ligases or E3s are components of the ubiquitin proteasome system (UPS) that coordinate the transfer of ubiquitin to the target protein. A major class of ubiquitin-ligases consists of RING-finger domain proteins that include the substrate recognition sequences in the same polypeptide; these are known as single-subunit RING finger E3s. We are studying a particular family of RING finger E3s, named ATL, that contain a transmembrane domain and the RING-H2 finger domain; none of the member of the family contains any other previously described domain. Although the study of a few members in *A. thaliana* and *O. sativa* has been reported, the role of this family in the life cycle of a plant is still vague. To provide tools to advance on the functional analysis of this family we have undertaken a phylogenetic analysis of ATLs in twenty-four plant genomes. *ATL*s were found in all the 24 plant species analyzed, in numbers ranging from 20–28 in two basal species to 162 in soybean. Analysis of *ATL*s arrayed in tandem indicates that sets of genes are expanding in a species-specific manner. To get insights into the domain architecture of ATLs we generated 75 pHMM LOGOs from 1815 ATLs, and unraveled potential protein-protein interaction regions by means of yeast two-hybrid assays. Several ATLs were found to interact with DSK2a/ubiquilin through a region at the amino-terminal end, suggesting that this is a widespread interaction that may assist in the mode of action of ATLs; the region was traced to a distinct sequence LOGO. Our analysis provides significant observations on the evolution and expansion of the ATL family in addition to information on the domain structure of this class of ubiquitin-ligases that may be involved in plant adaptation to environmental stress.

## Introduction

The accurate maintenance of protein levels is an essential regulatory mechanism in all biological processes. The ubiquitin proteasome system (UPS) is a pathway that has a major role in the destruction of proteins in eukaryotes. In plants, the UPS performs several amazing and complex tasks. It is involved in the regulation of key steps of almost all hormone responses, it participates in plant morphogenesis, has a role in chromatin structure and epigenetics, and modulates the defense response against diverse pathogens. Such diversity of roles may be a reason for the large number and variety of UPS pathway components in plants [1], [2].

There are three minimal UPS pathway components for protein degradation: a) the protein target, a suitable protein subject to degradation; b) the three ubiquitinating enzymes, ubiquitin-activating enzyme (E1), ubiquitin-conjugating enzyme (E2) and ubiquitin-ligases (E3); c) the 26S proteasome proteolytic complex consisting of the 19S regulatory particle (RP) and the 20S core protease, which recognizes and degrades the polyubiquitinated proteins. E3 ubiquitin-ligases are the regulatory components of the UPS, they specifically recognize the protein target for degradation and coordinate the transfer of the ubiquitin [1]. There are various classes of ubiquitin-ligases, many of which possess a RING finger domain. In the sequenced genomes, ubiquitin-ligase genes encoding the RING-finger domain are highly abundant. The RING finger E3s assist in the transfer of the ubiquitin by binding to the E2 ubiquitin-conjugating enzyme and bringing together both E2 and the substrate. There are two types of RING-finger E3s, one where the single-subunit includes both the RING-finger domain and the substrate recognition sequence in a single protein, and another in which the RING-finger domain and the substrate recognition sequences are different protein components that form a complex. More than 1400 E3 genes have been predicted in *A. thaliana*, and a little more than 600 are annotated in the human genome. Among them, the estimated number of single-subunit RING finger E3s is 477 in *A. thaliana* and 300 in humans [2], [3].

Frequently, additional domains are associated with RING-finger E3s. These may be important to localize these E3s to a particular subcellular compartment or to mediate interaction with the substrate or with other components of the UPS. These additional domains have been instrumental to define families of RING-finger E3s. Several domains have been predicted in RING-finger E3s, among which putative transmembrane domains or transmembrane helix regions are commonly found. These are present in at least 46 human and about 110 *A. thaliana* single subunit RING-finger proteins. Other commonly found features are coiled-coils, ankyrin repeats, BRCT, and zinc-fingers of various types. Based on domain organization human and *A. thaliana* RING-finger E3s have been classified in 17 and 18 major groups, respectively. However, a large number of RING-finger E3s, 73 in human and 140 in *A. thaliana*, did not show any recognizable conserved domain that could aid in their classification [4], [5].

Few RING finger E3s containing transmembrane domains have been characterized in detail. Of these, some of the best characterized members are involved in the endoplasmic reticulum-associated degradation (ERAD) pathway that recognizes and targets misfolded and naturally short-lived proteins to the UPS [6], [7]. The *S. cerevisiae* Hdr1p/Der3p and Doa10p are multispanning membrane proteins with cytosolic RING finger domains that exert distinct roles in ERAD. Aberrant proteins with luminal domains are slowly degraded and require Hdr1p/Der3p, whereas aberrant proteins with cytosolic domains are rapidly degraded and require Doa10p [8]. The human orthologues, pg78 and TEB4/March-VI function in a similar manner [9]. UPS has an important role in the fusion and fission of mitochondria, the transmembrane RING finger MARCH5 controls mitochondria morphology by binding to proteins that regulate mitochondrial homeostasis. MULAN, a double transmembrane domain RING finger protein, also regulates mitochondrial dynamics [10]. Transmembrane RING finger E3s have been implicated in the plant immune system. RIN2 and RIN3 are multispanning membrane RING finger proteins that interact with the RPM1 disease resistance NBS-LRR (nucleotide binding site-leucine-rich repeats) protein. OsRHC1 is a *O. sativa* multispanning membrane RING finger E3 that enhances defense response upon ectopic expression in *A. thaliana*, suggesting that it is involved in a wide range of disease resistances [11].

We are characterizing a particular family of RING finger E3s, named ATL that includes at least 80 members in *A. thaliana* and 121 in *O. sativa*. This family contains the RING-H2 variation of the canonical RING finger domain, in which the fifth cysteine residue is replaced by a histidine residue. A common feature present in all members of this family is a transmembrane domain located at the amino-terminal end. No other additional predictable domain was detected in any member of the family, although modules commonly conserved were inferred [12], [13]. The name ATL (Arabidopsis Tóxicos en Levadura) was given because *ATL2*, the first member of the family described, was identified as a conditionally toxic *A. thaliana* cDNA when overexpressed in *Saccharomyces cerevisiae* [14]. Supressors that alleviated the toxic phenotype in yeast were identified as mutations in deubiquitinases or distinct ubiquitin-conjugases, implying the involvement of UPS in regulating the function of ATL2 in yeast and suggesting that ATL2 targets an essential yeast protein for degradation [15].

Roles for various ATLs have been predicted. A number of ATLs may have roles in defense responses. ATL2 and ATL6 are rapidly and transiently induced in response to elicitors of the defense response [12]. ATL2, ATL6, ATL12, ATL13, and ATL16 are induced in the *A. thaliana eca* (expresión constitutiva de *ATL2*) mutants that show alterations on the expression of several defense-related genes [16]. LeATL6, a tomato orthologue of ATL6, is induced by elicitors and probably functions through a jasmonic acid dependent signaling pathway [17]. EL5, an *O. sativa ATL* gene, was also rapidly induced by elicitor treatment [18]. In some cases, mutant analysis provided information on the involvement of ATLs in defense-responses, as is the case for *A. thaliana* ATL9. A T-DNA insertional mutant line for ATL9 (At2g35000), a chitin induced gene, was more susceptible to powdery mildew. ATL9 has been proposed to participate in ERAD [19], [20]. Silencing lines for StRFP1, a potato ATL gene, were also more susceptible to *Phytophtora infestans*, while overexpressing lines showed diminished disease symptoms [21]. RING1/ATL55 was induced by pathogen attack and by the fungal toxin fumonisin B1 (FB1) that promotes cell death. Knock-down and overexpressing RING1/ATL55 lines did not show any apparent phenotype, although they exhibited FB1 hyposensitivity or hypersensitivity, respectively. Interestingly, RING1/ATL55 was localized to plasma membrane lipid raft subdomains [22]. Pleiotropic effects were also detected when ATLs were overexpressed. Expression of the *O. sativa ATL* OsBIRF1 in tobacco resulted in increased resistance to tobacco mosaic virus and *Pseudomonas syringae* pv *tabaci*, and also significant growth differences compared to wild-types lines [23]. Overexpression of PtaRHE1, a poplar ATL gene, showed ectopic expression of defense-related genes and WRKY transcription factors, as well as growth slower growth, delays in flowering, and alterations in leaf morphology and senescence [24]. Finally, alfalfa the *ATL* gene MsRH2-1 overexpressed in alfalfa or in *A. thaliana* also showed growth and developmental effects [25].

Specific physiological functions have been also inferred for ATLs. NIP2/ATL25 mediates intraplastid trafficking of RPOTmp, the T3/T7 phage-like RNA polymerase imported into both mitochondria and plastids that transcribes the rrn operon in proplasts/amyloplasts during seed imbibition/germination; NIP2/ATL25 was identified in a yeast two-hybrid screening using RPOTmp as bait [26]. CNI1/ATL31 is required in the regulation of the carbon/nitrogen response during post-germinative seedling growth transition [27]. A collection of *O. sativa* transgenic lines expressing dominant-negative mutant versions of the *O. sativa* EL5, revealed a role for this gene in regulating cell death during root development [28]. A Ds insertion in MEE16/ATL49 showed unpaired embryo development at the one-cell stage and a normal endosperm development [29]. DNF/ATL62, an essential repressor of photoperiod response in *A. thaliana*, was identified as an early flowering mutant under short-days [30].

Plants seem to have adjusted several gene families to respond to environmental cues. Whole genome duplication, segmental, and tandem duplications have been major events driving expansion of gene families through millions of years of evolution [31], [32]. We took advantage of the availability of entire genome sequences and gene annotation of several genomes from evolutionarily divergent plants to advance the study of the evolutionary history of the ATL family of RING finger E3s. We identified 1815 members of the ATL family from twenty-four plant genomes. We discovered potential motifs among groups of ATL proteins by generating 75 PSPM (position-specific probability matrix) LOGOs. Based on phylogenetic and motif organization we classified them into 9 groups of ATLs. Comparison of tandemly arrayed ATLs suggested that unique clusters of duplicates are expanding in many of the species. Potential regions in ATLs that mediate protein-protein interaction were identified by means of yeast two-hybrid assays. Our analysis revealed important evolutionary features of the ATL family as well as information concerning domain structure of this class of ubiquitin-ligases.

## Results

### Identification of RING finger ATLs in the Plant Kingdom

We have previously surveyed the *A. thaliana* and *O. sativa* genomes for members of the ATL gene family and predicted 80 *A. thaliana* and 121 *O. sativa* ATLs [13]. The study showed that only 60% of the *O. sativa* ATLs were clustered with *A. thaliana* ATLs, suggesting that either an important expansion of ATLs in *O. sativa* or a dramatic contraction in *A. thaliana* occurred since their divergence about 140–145 million years ago [33], [34]. To improve the accuracy of the inferred ATL phylogenies and to further explore the domain diversity of this class of E3s, we identified members of the ATL gene family from 24 plant genomes. First, we queried for ATLs by performing BLASTP and hidden Markov model (HMM) searches using a canonical ATL RING-H2 domain. This canonical RING-H2 domain is a 42 amino acid long consensus sequence obtained after comparing *A. thaliana* and *O. sativa* ATLs. A collection of 5232 hits was initially retrieved (see Table S1), that roughly consisted of all RING-H2 sequences. From these sequences, we then retrieved 2132 that possessed the six cysteines and the two histidines that coordinate in zinc ligation with exactly the same spacing between them as the canonical ATL RING-H2. All of them contained a tryptophan residue spaced three residues downstream from the sixth zinc ligand; this residue is invariable in most RING finger domains [5]. Then we inspected these proteins for the presence of transmembrane helices towards the amino-terminus and for the previously described GLD motif. This is a conserved 12–16 amino acids motif that often begins with glycine, leucine and aspartic acid residues and is located between the transmembrane helices and the RING-H2 domain [13]. 1815 proteins contained at least one transmembrane helix as determined by the TMHMM Server v. 2.0 (http://www.cbs.dtu.dk/services/TMHMM/) [35]; about 82% of these also included the GLD motif (see below). Similar domain architecture between ATL family members suggests that they were related by origin and evolved from a common ancestor. In 17 of the 24 plant genomes at least 85% of the retrieved proteins that contained the canonical ATL RING-H2 domain also included a transmembrane helix, suggesting that this particular domain architecture in ATLs has been subjected to a strong selection during evolution (see Table S1).

**Figure 1:**
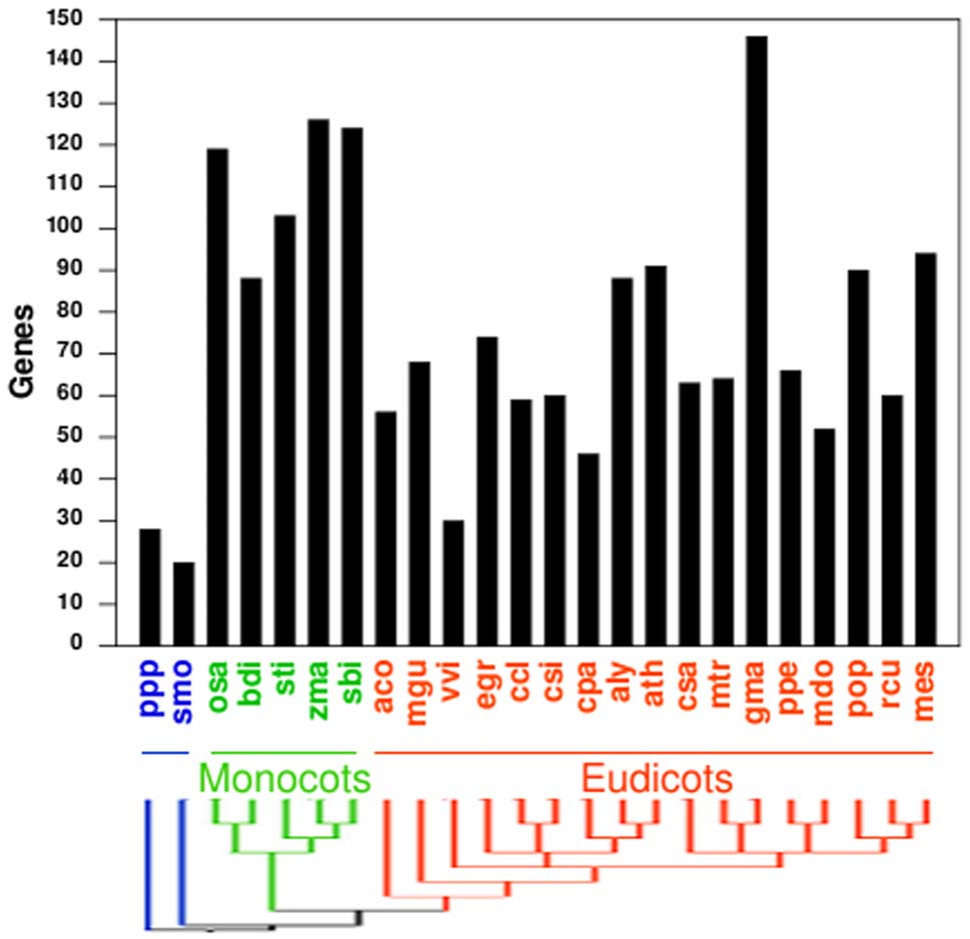
Number of retrieved *ATL*s in 24 plant species. The phylogenetic relationship between the 24 plant species is displayed at the bottom; it was adapted from the National Center of Biotechnology Information (NCBI) taxonomy server (http://www.ncbi.nlm.nih.gov/Taxonomy). The three major groups of plants are: basal plants (blue), monocots (green) and eudicots (orange). The species abbreviation is: ppp, *Physcomitrella patens*; smo, *Selaginella moellendorfii*; osa, *Oryza sativa*; bdi, *Brachypodium distachyon*; sit, *Setaria italica*; zma, *Zea mays*; sbi, *Sorghum bicolor*; aco, *Aquilegia coerulea*; mgu, *Mimulus guttatus*; vvi,*Vitis vinifera*; egr, *Eucalyptus grandis*; ccl, *Citrus clementina*; csi, *Citrus sinensis*; cpa, *Carica papaya*; aly, *Arabidopsis lyrata*; ath, *Arabidopsis thaliana*; csa, *Cucumis sativus*; mtr, *Medicago truncatula*; gma, *Glycine max*; ppe, *Prunus persica*; mdo, *Malus domestica*; pop, *Populus trichocarpa*; rcu, *Ricinus communis*; mes, *Manihot esculenta*.

ATLs were recovered from all the explored plant genomes: two basal species, moss and lycopod, and twenty-two angiosperms, five monocots, and seventeen eudicots (Figure 1, and Table S1). The number of retrieved ATLs from basal species was much lower than that obtained from the angiosperms, suggesting that ATLs underwent expansion in higher plants. *V. vinifera* seems to be an exception among angiosperms, having the lowest number of *ATL*s. In angiosperms the number of ATLs ranges from 30 in *V. vinifera* to 146 in *G. max*, and except for *G. max*, the mean number of ATLs is higher in monocots (112 ATLs) than in eudicots (71 ATLs), indicating that a differential gene rate of gene expansion may have occurred in these two groups since the split of the two major groups of angiosperms.

**Figure 2:**
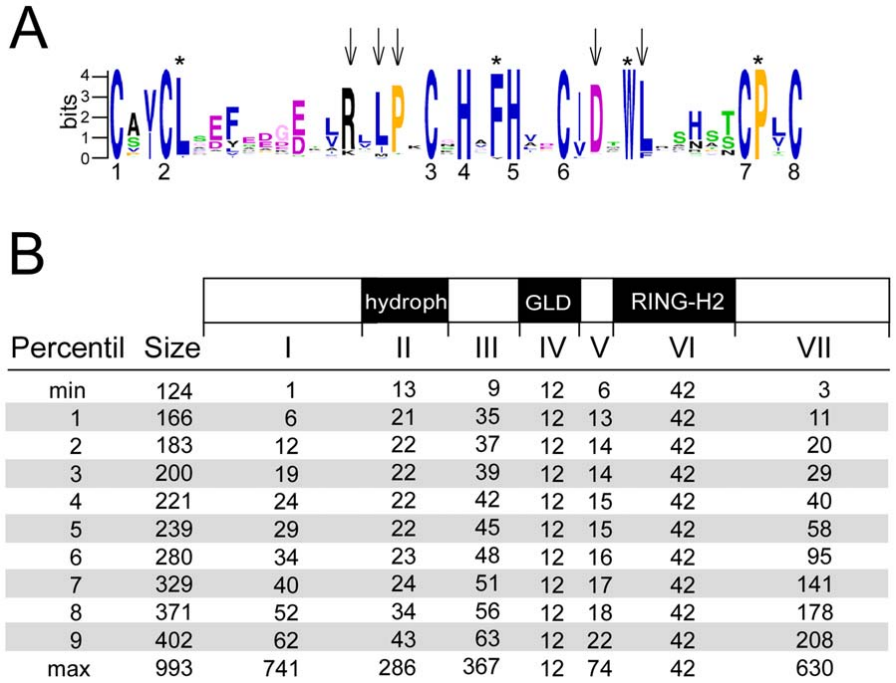
A broad view of domain architecture in ATLs. (A) The sequence LOGO represents the consensus sequence of the ATL RING-H2 domain. Numbers indicate the residues involved in zinc ligation, asterisks indicate the residues conserved in RING fingers, and arrowheads residues conserved in ATLs. (B) Schematic representation of a canonical ATL with its seven regions (I–VII). The second column displays the size diversification in number of amino acids sorted independently according to the statistic percentile.

The sequence LOGO generated for the ATL RING-H2 domain from the collected sequences showed conserved amino acid residues in addition to the canonical zinc ligands (Figure 2A). Some of these amino acids have been previously detected as commonly conserved residues in several classes of RING finger domains [5]. For instance, a leucine following the second metal ligand, a phenyl alanine preceding the fifth ligand, a tryptophan following four residues from the sixth ligand, and a proline next to the seventh ligand (labeled with asterisks in Figure 2A). Besides the conservation in distance between the eight zinc ligands found in ATLs, two patterns were frequent in the RING-H2 domain: [I/V]D-x_1_-WL, after the sixth metal ligand, and a R-x_1_-LP pattern, spaced by a single residue upstream from the third zinc ligand (denoted with arrowheads in Figure 2A). Some of the conserved residues may have a role in the specific interaction between the E2, ubiquitin-conjugating enzyme, and the ATL RING-H2 domain [36], [37]. These features suggest that the RING-H2 domain in ATLs exhibits a selective pressure for conservation throughout evolution and that a direct correlation exists for the co-occurrence of transmembrane helices and a particular type of RING-H2 domain.

An assessment of the size of ATL proteins showed high variability among them, ranging from 124 to 993 amino acid residues (Figure 2B, second column). Aside from the RING-H2 domain, the GLD motif and the transmembrane helix, no other recognizable domains were detected either in previous or in the current study of ATLs (searches on the Pfam database using cutoff value e^−1^). To obtain a broad view on the domain architecture of ATLs, we first inspected the size distribution of various regions of the ATL proteins (see Figure 2B), organizing them by size to observe in which regions the increased size occurred. The RING-H2 and the GLD regions displayed a specific size in all ATLs (regions IV and VI, respectively), and the size of the predicted transmembrane helices at the amino-terminus was usually 22–24 residues long (region II). Most ATLs contained three transmembrane helices or less (99.3%), rare exceptions included ATLs with regions containing up to thirteen transmembrane helices (Figure 2B; region of 286 amino acids, that corresponds to aly|354960). Regions that may uncover new important features were adjacent to these three regions. The region showing most size variability was that at the carboxy-terminus following the RING-H2 domain (region VII). It may be absent in some members or display an extension of several hundreds of residues. The amino-terminal region can also be absent or include several tens of residues upstream to hydrophobic helix (region I). The two regions between the hydrophobic helix and the RING-H2 domain display less size variation (regions III and V).

#### Phylogenetic Distribution of RING finger ATLs in Plants

To obtain consistent phylogenies we carried out different types of analysis. We compared trees generated from complete gene sequences, trees obtained from concatenated motifs, as well as trees based on the 42 amino acids segment encompassing the RING-H2 domain. Since we previously observed a high degree of diversity among ATL protein sequences, we reasoned that eliminating highly divergent regions would generate more consistent phylogenies as suggested in other reports [38]. Indeed, trees generated with the RING-H2 domain or the RING-H2 concatenated to the GLD segment generated trees showing better resolution of species in many clades and better support of the branch classifications than the trees based on the entire protein sequences. The phylogenetic trees were generated both by distance matrix methods (neighbor-joining, NJ) and by maximum likelihood methods (FastTree). The topology observed by these two methods was basically the same. In order to classify ATLs, we chose to work with trees based on the 42 amino acids sequence of the RING-H2 generated by FastTree.

**Figure 3:**
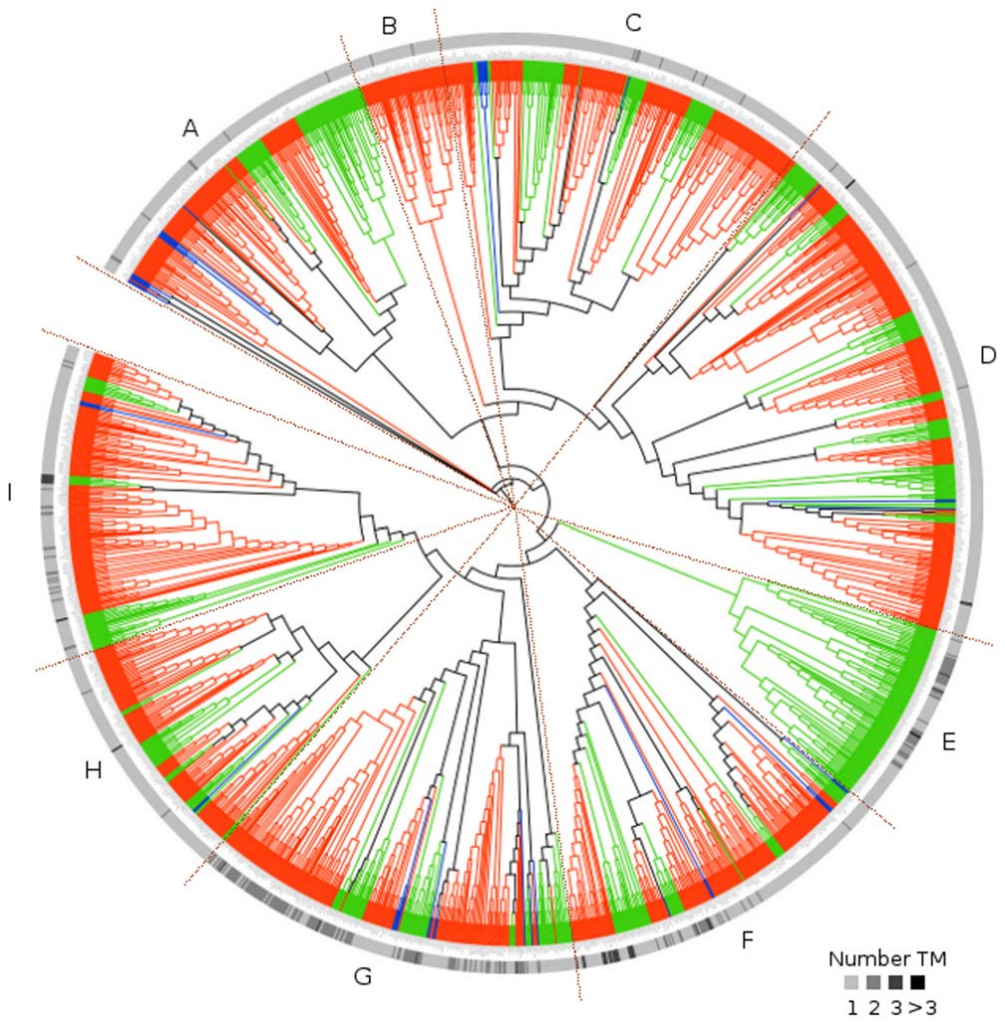
Phylogeny of 1815 ATL proteins from 24 plant species. The 42 amino acid sequence of the RING-H2 was used to generate the tree with FastTree; the tree is displayed in Figure S1. The branches were classified in 9 groups, A to I, collapsing branches with local support below 80%. Basal species are colored in blue, monocots in green and eudicots in orange. An outside circle represents presence of one or more transmembrane regions. ATLs have one or more transmembrane helix region toward the amino-terminal end, according to TMHMM, these are shown on a scale of shading. The basal gray color corresponds to one transmembrane helix.

**Figure 4:**
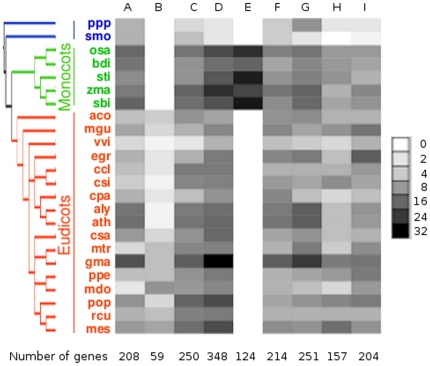
Distribution of ATLs from 24 plant species in 9 groups. Heat map representation of the number of ATLs from the 24 species in each one of the nine groups by a gray scale. The total number of genes in each group is shown at the bottom (the distribution of the 1815 ATLs in 9 groups in displayed in Table S2). The species tree is as in Figure 1.

The phylogenetic tree containing all 1815 ATLs consisted of two major clades and a set of three minor basal clades (see Figure 3, the phylogenetic tree is resolved in Figure S1). Based on a collapsed branch tree with local support less than 80% we classified clades in 9 ATL groups (A to I). The ATL proteins with a single transmembrane helix, that represented about 86% of all ATLs, were distributed in all the groups. Most ATLs containing two or three transmembrane helices were clustered in groups F and G or F, respectively (see the outside circle in Figure 3). These two groups included single members from the basal plants, indicating that they may correspond to the ancestral genes of these lineages. All groups included basal plant species and both monocots and eudicots, except for group B which had only eudicots sequences and group E that comprised only monocot sequences (see Figure 4). Groups B and E may represent *ATL* genes acquired after the split of the two major groups of flowering plants, suggesting that the ATL family has been subjected to lineage specific expansion. The presence of basal species in almost all the groups indicated that they might correspond to the primary group of *ATL* genes that were conserved and even expanded in higher plants throughout evolution.

#### PSPM LOGOs for Visualization of ATL proteins

**Figure 5:**
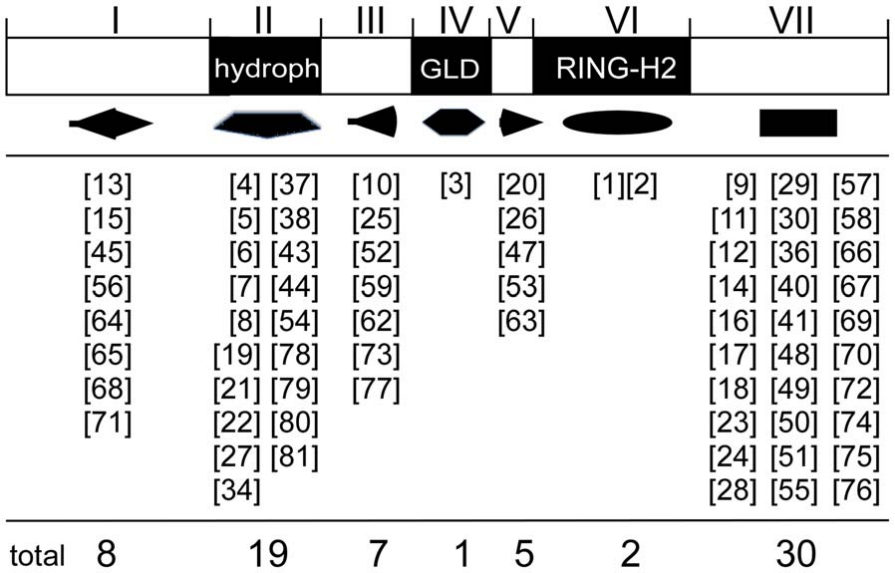
Sequence LOGOs mapped to ATL regions. Below the canonical ATL diagram, geometric figures represent the seven regions and the sequence LOGOs mapped to each region are shown. The total number of logos per region is indicated at the bottom. The catalog of the 75 sequence LOGOs is shown in Table S3.

In view of the fact that the degree of sequence identity among the ATL proteins is highly variable outside the RING-H2 zinc finger and that besides the transmembrane helix no other recognizable protein domain was detected, we performed MEME software package searches for all the 1815 ATL proteins. MEME searches were carried out with the aim to discover potential motifs in ATLs that may have a specific function and to assist with the classification of these proteins. Based on the PSPM sequence, 75 non redundant sequence LOGOs were generated ranging from 6 to 50 residues long; a maximum of two reiterations of a LOGO on a single protein was observed. The MAST software package was used to generate sequence LOGOs and to search for structural domains at PDB (Protein Data Bank, http://www.rcsb.org/pdb/home/home.do); only the RING-H2 domain was present in PBD (ID: 1IYM). To create a better visualization of the protein domain architecture we relied on shape and color codes using tools from the iTOL software (see Materials and Methods). A single geometric shape was used for each one of the 7 regions within an ATL and a different color for each different LOGO that was mapped to a region. In the cases in which the LOGO encompassed two adjacent regions it was mapped to the region containing most of the sequence (see Figure 5; sequence LOGOs along with the phylogenetic tree are shown in Figure S1)

Two sequence LOGOs were obtained within the RING-H2 domain (LOGOs 1 and 2) and one in the GLD (LOGO 3) motif, confirming their conservation and distinctiveness among ATLs (the display of the 75 sequence LOGOs is shown in Table S3). The region for which the greatest number of LOGOs was obtained was region VII, corresponding to the carboxyl-end of the protein; this is the region most diverse in size (see Figure 2B). Nineteen sequence LOGOs were mapped to the region encompassing transmembrane helices, and visual inspection of the sequence showed that 14 of them contained tracks of at least 15 hydrophobic residues (LOGOs 4, 6, 7, 8, 19, 21, 22, 34, 38, 44, 78, 79, 80, 81); the other 5 LOGOs were generated from sequences located at the boundaries of region II. LOGO #7 was generated mainly from hydrophobic residues, and it generally mapped toward the transmembrane helix proximal to the amino-terminus of those ATLs that contain two successive hydrophobic regions (see group G, Figure 3 and Figure S1). Likewise, LOGOs 38, 6, and 5 converge in ATLs that contain three predicted hydrophobic regions (see group F, Figure 3 and Figure S1). Region I generated 8 sequence LOGOs, four of which showed conserved serines (LOGOs 13, 38, 65, 68). Seven sequence LOGOs were mainly mapped to region III; these are short sequences that include regions rich in glutamine (LOGO 10), acidic amino acids (LOGOs 59, 73) or arginine (sequence LOGO 77). Likewise, five sequence LOGOs mapped to region V, three of them are rich in lysine or arginine residues (LOGOs 20, 26, 53). Finally, three logos were not located to a specific region but were scattered in different regions on various ATL proteins. These three LOGOs that mapped to either regions I, III or VII consisted of tracks of 10 prolines (LOGO 39), 7 glycines (LOGO 46), or 7 glutamines (LOGO 60).

### Diversity of ATL-generated sequence LOGOs in Plants

**Figure 6:**
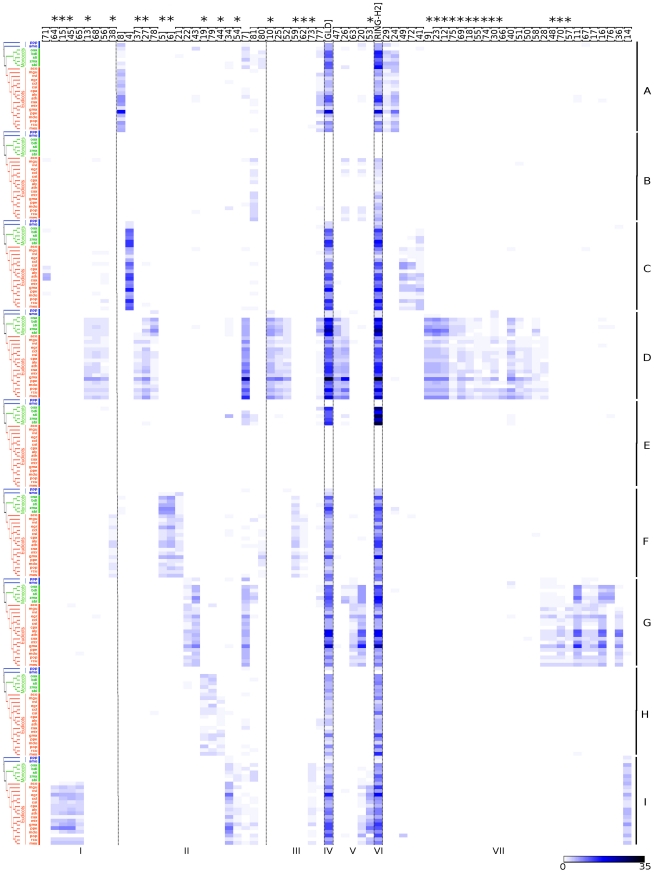
Distribution of 75 sequence LOGOs on the 9 ATL groups. A heat map shows the frequency of the conserved motifs in each of the nine ATL groups. Asterisks on top point to sequence LOGOs that are specific to a group. The GLD motif and RING-H2 are widely spread among all groups except for the GLD motif that is absent from group B. Roman numbers I–VII represent the seven ATL regions; the frequency of conserved motifs is shown according to a blue scale. The species tree is as in Figure 1.

With the purpose of evaluating the diversity of motifs in ATLs among species, we investigated the distribution of the 72 non-scattered sequence LOGOs in the 24 plant species. We generated graphical data displaying the occurrence of each sequence LOGO and its relative frequency in the 9 ATL groups (see Figure 6). A distinct modular organization was revealed for each one of the 9 groups. All groups showed the sequence LOGOs typical of the ATL family: at region VI, the RING-H2 domain; at region II, transmembrane helices and at region IV, the GLD LOGO (except for group B). Except for region I, sequence LOGOs were obtained in all regions from lower species, and to a much lower extent than in all angiosperms, suggesting that new LOGOs were acquired throughout evolution to constrain the diversification at different levels in different regions. The number of sequence LOGOs generated varied in each group. Groups D and G containing all species, included a large number of LOGOs scattered through all regions. This is not an effect of sample size as similar numbers of members are found in each group. In contrast, groups B and E which included only eudicots or monocots, respectively, exhibited a reduced number of LOGOs. Regions I and VII showed drastic differences in the number of LOGOs; in several groups LOGO-content in region I (groups A, B, C, G, H) or in region VII (groups B, E, F, H) was highly reduced. About 41% of the sequence LOGOs were found to be group specific and 20% of them were present in basal plants, suggesting both an ancient origin and a novel acquisition of sequence LOGOs during the evolution of ATLs (asterisk in Figure 6).

The functional analysis of ATLs is scarce and to date only a few members have been studied. In Table 1 we summarized the general features of some ATLs and their predicted protein domain architecture models based on the sequence LOGOs. It is readily observed that *A. thaliana* ATL2, poplar PtaRHE1 and *O. sativa* OsEL5 are included in the same ATL group and share a similar modular composition and a related role. Likewise, *A. thaliana* ATL6 and ATL9 both belong to group G and are involved in elicitor-responses. On the other hand, there are ATLs that share the same group and a similar protein domain architecture model, but whose predicted role is quite different. For instance, *A. thaliana* MEE/ATL49 and *O. sativa* OsBIRF1/ATL36, and *A. thaliana* ATL6 and CN1/ATL31.

**Table 1:** Classification and Modular Organization of Studied ATLs.

Gene	Predicted role	Group	Modular Organization^1^
*ATL2*	Early-elicitor response	A	[8][77][GLD][RING-H2][24]
*PtaRHE1/POPTR_0002s23000*	Ectopic expression of defense-related genes and pleiotropic effects when expressed in tobacco	A	[8][77][GLD][RING-H2][24]
*OsEL5/OsATL24*	Rice ATL, early-elicitor response. Regulation of cell death during root development	A	[77][GLD][RING-H2][39] [24]
*DFN/ATL62*	Repressor of photoperiod response	A	[GLD][RING-H2]
*RING1/ATL55*	Sensitivity to fumonisin B1 and pathogen attack. Located to plasma membrane lipid rafts	D	[39][GLD][26][RING-H2]
*MEE/ATL49*	Embryo development	D	[7] [27] [10][GLD][47][RINGH2][23] [9] [75] [12] [28]
*OsBIRF1/OsATL36*	Rice ATL, increased resistance to tobacco mosaic virus and Pseudomonas syringae when expressed in tobacco	D	[39] [46] [7] [27] [10][GLD][47][RINGH2][51] [23] [9] [69] [12]
*NIP2/ATL25*	Mediates intraplastidial trafficking of RPOTmp at thylakoid membranes	F	[38] [6] [5] [59][GLD][RING-H2]
*ATL43*	ABA response	G	[22][GLD][63][RING-H2][17] [67]
*ATL6*	Early-elicitor response	G	[7] [43][GLD][20][RINGH2][16] [11] [36] [70]
*CN1/ATL31*	Regulation of carbon/nitrogen response	G	[7] [43][GLD][20][RINGH2][16] [11] [36] [70]
*ATL9*	Chitin-induced, resistance to powdery mildew. Located to ER	G	[GLD][20][RING-H2][16] [11] [36]

1 A schematic representation of the modular organization can be found in Figure S1.

### Tandemly Arrayed ATL genes

The complete genome sequences of distant and closely related species provide the means to evaluate the evolutionary history, specialization and expansion that may occur within members of a gene family. Gene expansion and diversification events in plants are thought to occur through gene duplication. Moreover, tandem gene duplication events seems to occur at higher frequency among genes encoding membrane proteins and stress response proteins [39], [40]. Based on the fact that ATLs may be membrane associated proteins and that several of them are involved in stress responses (see Table1) we decided to evaluate the significance of tandem gene duplication on the ATL family structure and evolution.

We identified and compared the extent of tandemly arrayed genes in the 24 plant genomes. The tandemly arrayed ATLs were inferred from the locus name and by inspecting the arrangement of the genes on the chromosomes and/or scaffolds in the Gbrowse at the Phytozome database. In most cases, the number of genes for each tandem array was 2 or 3. The number of tandemly arrayed ATLs and the percentage of tandemly arrayed ATLs in each species were highly variable. Some species had few tandemly arrayed ATLs (2 to 5) and some had more than 20; similarly, for some species the percentage of tandemly arrayed ATLs was low (2 to 6%) but for others was much higher (more than 20%) (see Table 2). For instance, a notable difference was observed among grasses: *S. bicolor* contained 15 tandem arrays, corresponding to 36% of the ATLs whereas *Z. mays* contained 6 tandem arrays corresponding to 9% of its members (see Table 2). These features may be indicative of the role of birth-and-death evolution mechanism in *ATL*s to have sets of genes expanding in a lineage specific manner. About 90% of the tandem array clusters belong to the same ATL group, supporting the fact that they were related by domain architecture and arise by unequal crossing-over duplication events (Table S4). The pairs that did not group showed dissimilar domain architecture suggesting that the duplicates underwent specialization or that they were clustered together by a gene conversion event. Interestingly, the group distribution and the domain architecture of most of these duplicated pairs were conserved among various species, suggesting a common origin for this event (see Figure S2).

**Table 2:** Tandemly arrayed ATLs.

	tandemly arrayed ATLs	tandemly arrayed ATLs %^1^	tandem arrays	genes per tandem array	A	B	C	D	E	F	G	H	I
ppp	4	14	2	2,2							1		
smo	5	25	2	2,3	1								
bdi	27	23	8	5,5,4,3,2,2,4,2	2				4		1	1	
osa	22	25	9	3,4,3,2,2,2,2,2,2				1	4		1		
sti	13	13	5	3,2,3,2,3	1				2		1	1	
zma	12	9	6	2,2,2,2,2,2				1	4		1		
sbi	45	36	15	2,2,3,2,9,2,2,2,2,2,5,2,2,4,4	4		1	1	5		3	1	
aco	10	18	4	2,3,2,3		1				1	1		1
mgu	9	13	3	2,5,2						1			1
vvi	2	6.8	1	2								1	
egr	16	22	7	3,2,2,2,2,3,2	1		1			1	1		2
ccl	9	15	3	2,3,4			2						1
csi	6	10	3	2,2,2			2					1	
cpa	10	22	4	2,2,2,4			2			1			
ath	20	23	8	4,2,2,2,2,3,2,3	2		2			2	2		
aly	14	15	6	3,2,2,2,3,2	2		2				2		
ppe	15	23	7	2,2,3,2,2,2,2		1					1	1	3
csa	4	3	2	2,2							1		
gma	14	21	7	2,2,2,2,2,2,2				2			1		1
mtr	2	4	1	2									1
pop	11	12	5	2,2,3,2,2			2					1	2
rcu	2	3	1	2		1							
mes	4	4	1	4								1	
Total	276		110										

1 Percentage of tandemly arrayed ATLs over the total number of ATLs in that specie. Locus names of tandemly arrayed ATLs are displayed in Table S4. A to I correspond to the 9 ATL groups, as in Figure 3. Thirteen pairs that did not group together were not included; they are shown in Figure S2. mdo was not analyzed.

### Potential Regions in ATLs Mediating Protein-Protein Interactions

**Figure 7:**
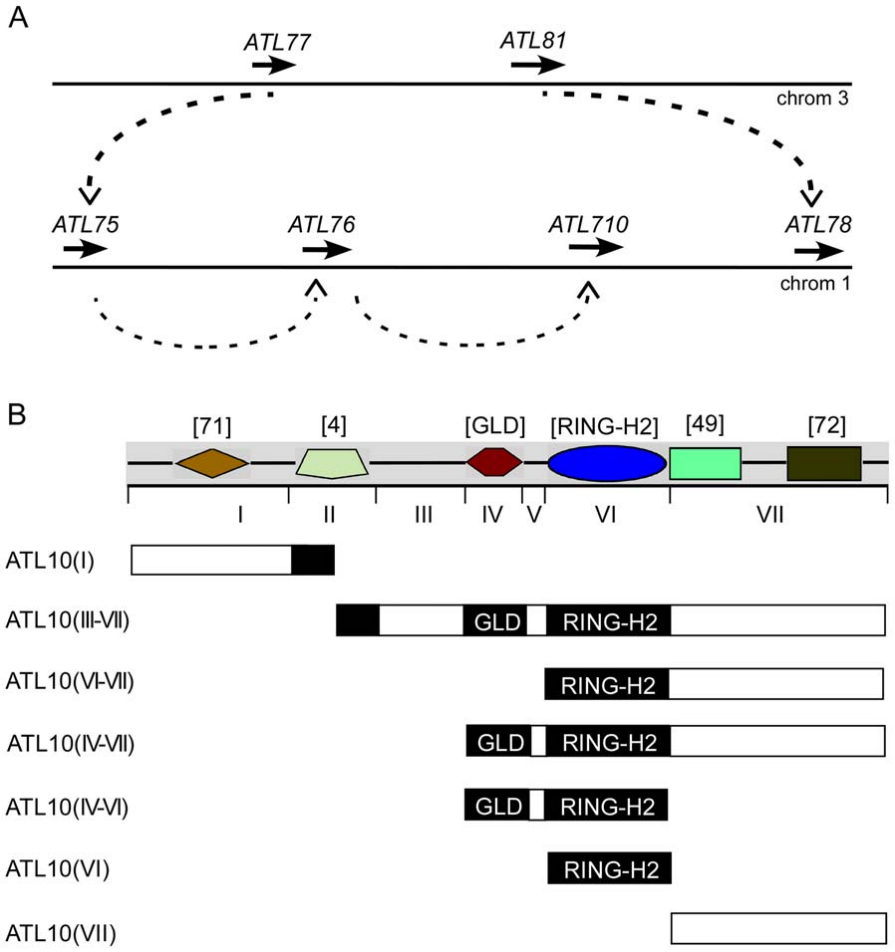
Inferred evolutionary history and organization of *ATL10*. (A) The inferred evolutionary history of *ATL10* is depicted. A segmental duplication event involving chromosomes 1 and 3 generated *ATL78* and *ATL75*, from *ATL81* and *ATL77*, respectively; *ATL10* was then generated by tandem duplication events from *ATL75*. (B) The upper diagram depicts the modular organization of ATL10 based on sequence LOGOs mapped to it. Below is the schematic representation of seven clones encompassing different regions of ATL10 that were used in yeast two-hybrid assays.

Protein-protein interactions are necessary for the functioning of ubiquitin-ligases. RING finger E3s are modular proteins encoding at least the RING domain that binds to the E2 ubiquitin-conjugating enzyme, and a domain for substrate recognition; additional associated domains may be important to modulate their function. Our study on ATL-generated sequence LOGOs suggested that ATLs encoded possible conserved domains (see Figures 5 and Table S3). We presume that some of these domains might be involved in substrate recognition or interaction with additional proteins that may assist in the function of the E3. To unravel possible interacting domains in ATLs, we performed yeast two-hybrid screens with *A. thaliana* ATL10. According to our classification, ATL10 belongs to a cluster of duplicated genes within group C. This cluster consisted of two tandem gene arrays, one located on chromosome 1, containing four genes (ATL75, ATL76, ATL10 and ATL78) and another on chromosome three, containing two genes (ATL77 and ATL81). Based on the established *A. thaliana* established genome duplication events, we inferred that these sets of genes were generated first by a segmental duplication event during the emergence of the brassicaceas, such that ATL81 and ATL77 on chromosome 3 generated ATL78 and ATL75 on chromosome 1, respectively. Then, ATL76 and ATL10 were generated by tandem duplication events (see Figure 7A).

We searched for interactive clones using as baits distinct segments of ATL10: ATL10(I) that contained the amino-terminus end, ATL10(III–VII) that included a region beginning at the boundary of the transmembrane helix and finishing after the carboxy-terminus, and ATL10(VI–VII) that contained the RING-H2 domain and the carboxy-terminus (see schematic of clones in Figure 7B) (we did not use as bait interactive clones containing the transmembrane helix because we assumed that it did not have a role associated with ubiquitin-ligase activity, since the function of this putative sequence would be to target the protein to membranes).

One of the recovered interactive clones from the yeast two-hybrid screen with the amino-terminus bait ATL10(I) was a truncated cDNA coding DSK2a, a protein that belongs to a family of Ubiquitin-like/Ubiquitin-associated (UBL/UBA) conserved among eukaryotic organisms [41], [42]. The *S*. *cerevisiae* and the human orthologues of DSK2a are DOMINANT SUPPRESSOR OF KAR2 (DSK2) and ubiquilin, respectively [43], [44]. Few interacting clones were isolated using ATL10(III–VII) as bait whereas the yeast two-hybrid screen using ATL10(VI–VII), that lacks the III, IV and V regions, resulted in a collection of several putative interactive clones. We reasoned that the basis for the difference in the number of interactive clones was that a specific sequence in the ATL10(III–V) segment influenced the secondary structure of the ATL10(III–V) bait in a way that hampered the yeast two-hybrid assay. This segment may be related to the highly flexible random coil structure that complicates the determination of three-dimensional RING-H2 structure [36]. Under our selective conditions, we obtained four positive clones with the ATL10(III–VII) bait and twenty one positive clones with the ATL10(VI–VII) bait (see Table S5). We then mapped the segment mediating the interaction with the yeast two-hybrid prey, and found that for all of them region VII in ATL10 was responsible for the interaction; this region included sequence LOGOs 49 and 72 (see Table S5 and Figure 7B). Thus, our yeast two-hybrid screens suggest that at least two novel regions in ATL10 may mediate protein-protein interactions, one located at the amino-terminal end and one toward the carboxy-terminal end.

### Interaction of DSK2a/ubiquilin with ATLs

Since one function of the Dsk2a/ubiquilin proteins is to facilitate degradation by the proteasome [41], [42], we reasoned that they might play an important role in the activity of ATL10 or in ATLs in general. Dsk2a/ubiquilin is a multidomain protein that possesses two distinct domains and a central region, and we decided to determine which domain was mediating the interaction with ATL10(I). In Dsk2a/ubiquilin-like proteins the UBL (UBiquitin-Like) domain (that interacts with the RP component of the 26S proteasomes), is located at the amino-terminus, while the UBA (UBiquitin-Associated) domain, (that binds ubiquitin and multi-ubiquitin chains) is located at the carboxy-terminus. The central region of the protein possesses stress-inducible 1 (STI1)-like domains that were originally described in proteins that bind HSP70 [41], [42].

**Figure 8:**
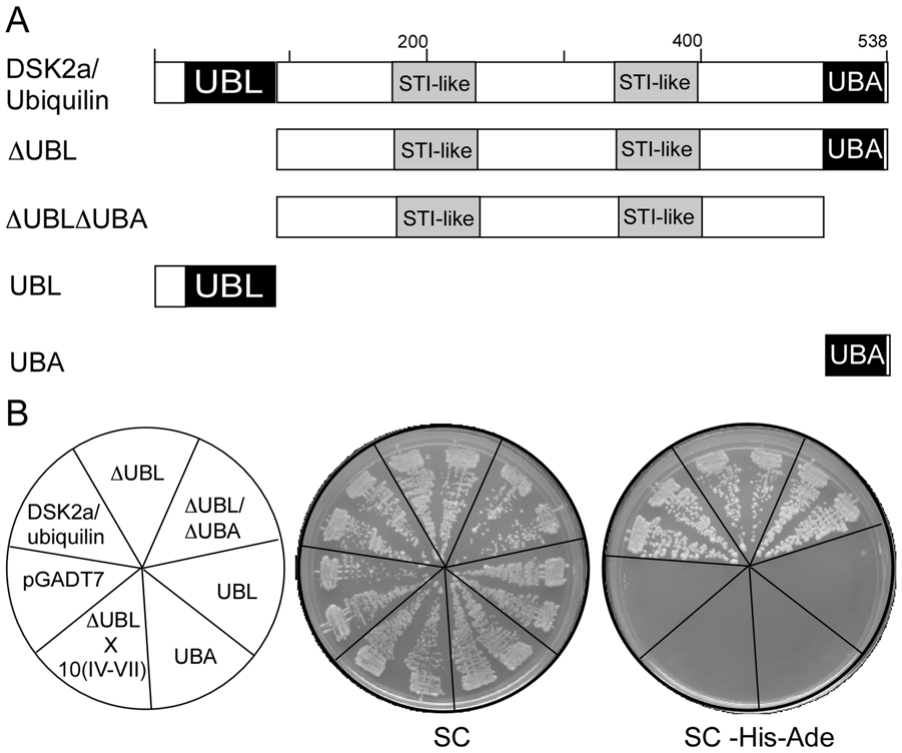
Interaction of DSK2a/ubiquilin with ATL10. (A) Schematic representation of DSK2a/ubiquilin showing UBL, UBA, and STI-like domains and derivative clones generated for the yeast two-hybrid assays; ΔUBL represents a clone obtained from the yeast two-hybrid screening. DSK2a/ubiquilin-containing fragments were ligated into the activation domain of pGADT7. (B) Representative plates showing yeast two-hybrid interactions between DSK2a/ubiquilin derivative clones and ATL10(I); the left panel shows the template of the plates. The yeast strain AH109 was cotransformed with pGBKT7 and pGADT7 derivatives, selecting for transformants on SC lacking Trp and Leu. Transformants were then streaked onto SC medium lacking Trp and Leu (SC) and onto SC medium lacking Trp, Leu, His and Ade (high-stringency selective conditions). The plates were incubated at 30°C for four days; growth is seen as dense streaks of yeast over background. The interaction of ATL10(IV–VII), a clone lacking region (I), with ΔUBL was also included as a negative control.

**Figure 9:**
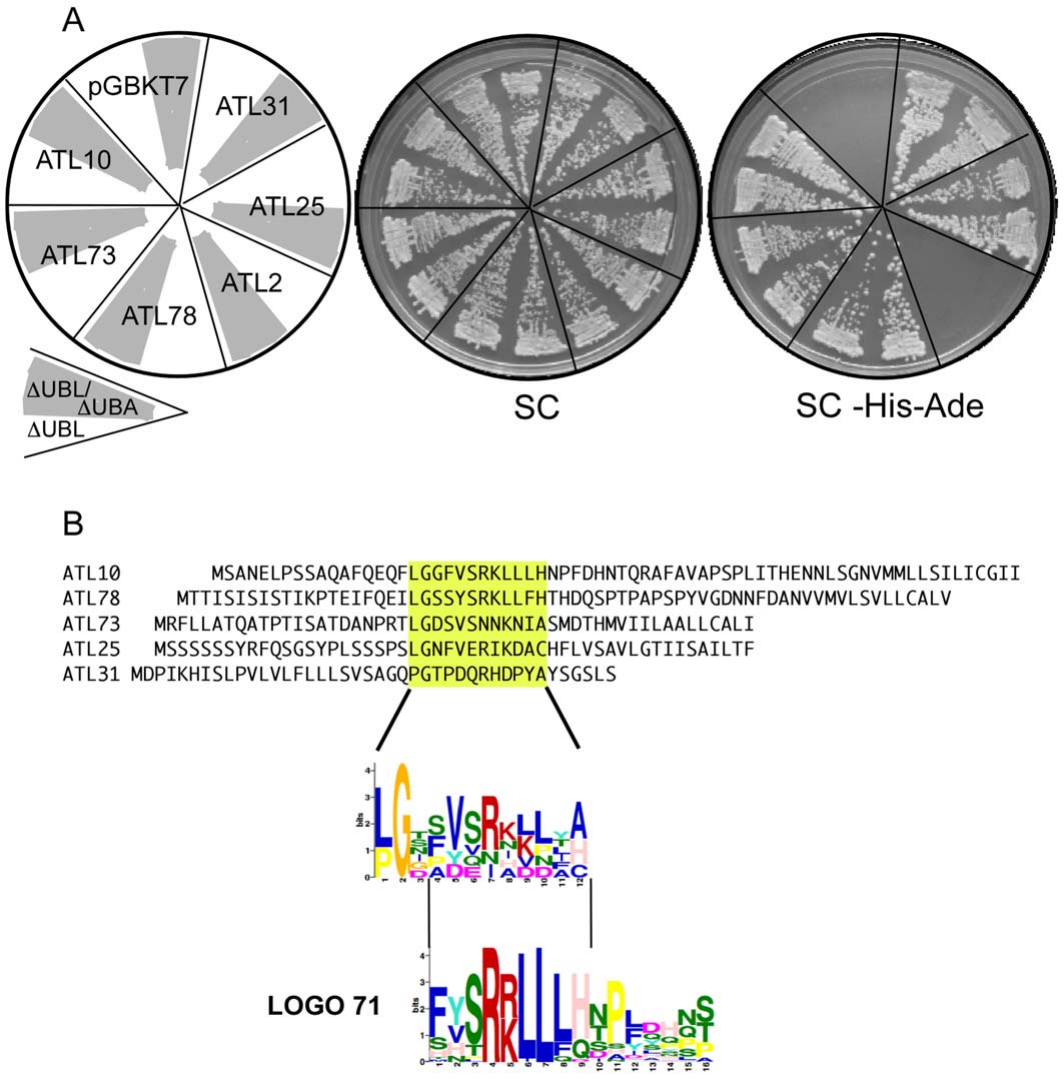
Interaction of DSK2a/ubiquilin with diverse ATLs. (A) Representative plates showing yeast two-hybrid interactions between DSK2a/ubiquilin and region I from six ATL genes; the left panel shows the template of the plates. The 6 ATL-containing fragments were ligated into the DNA-binding domain of pGBKT7, the two DSK2a/ubiquilin clones, ΔUBL and ΔUBLΔUBA (depicted in gray in the template plate), are as in Figure 8. The yeast two-hybrid assay was performed as in Figure 8. (B) A sequence LOGO in region I of ATLs. The MEME suite was used to generate sequence LOGOs from the 5 yeast two-hybrid clones shown in (A) and from the ATL2 clone. A sequence LOGO resulted from the region highlighted in yellow in the five clones; this region was not found in ATL2. As comparison, this sequence LOGO placed over the sequence LOGO 71 is shown below.

We tested the full-length DSK2a/ubiquilin as well as clones of UBL, UBA and a central region containing STI1-like sequences (see Figure 8A). We detected interaction with the central region and with a clone lacking UBL, which corresponds to the truncated cDNA identified as the interacting clone in the yeast two-hybrid screening. The full-length DSK2a/ubiquilin and the central region bind ATL10(I). Neither the UBL nor the UBA clones or ATL10(IV–VII), which does not include region ATL10(I), mediate yeast two-hybrid interactions (see Figure 8B). Next, we evaluated the interaction of DSK2a/ubiquilin with segments containing region I of five different ATLs: two phylogenetically related to ATL10 and three distantly related clones. We found that ATL73 and ATL78 (closely related ATLs from group C) and ATL25 and ATL31 (more distantly related from groups F and G, respectively) showed interaction with DSK2a/Ubiquilin, whereas ATL2 (also phylogenetically distant from group A) did not (see Figure 9A). In this assay we tested the interaction with the central region of DSK2a/Ubiquilin and the clone lacking UBL (see Figure 9A, clones ΔUBLΔUBA, and ΔUBL). We searched for a common motif in the ATLs that showed interaction with DSK2a/Ubiquilin and found that these ATLs share some residues of a sequence LOGO. This sequence is part of LOGO 71 that was specific to ATLs from group C (see Figure 9B; this sequence LOGO was not present in ATL2). Thus, related sequences to sequence LOGO 71 are also conserved in phylogenetically distant ATLs. Conservation and the ability of this region to interact with DSK2a/Ubiquilin suggest a widespread role for the DSK2a/Ubiquilin in ATL E3s.

### Interaction of the RING-H2 Domain of ATL10 with the Ubiquitin-conjugase UBC11

**Figure 10:**
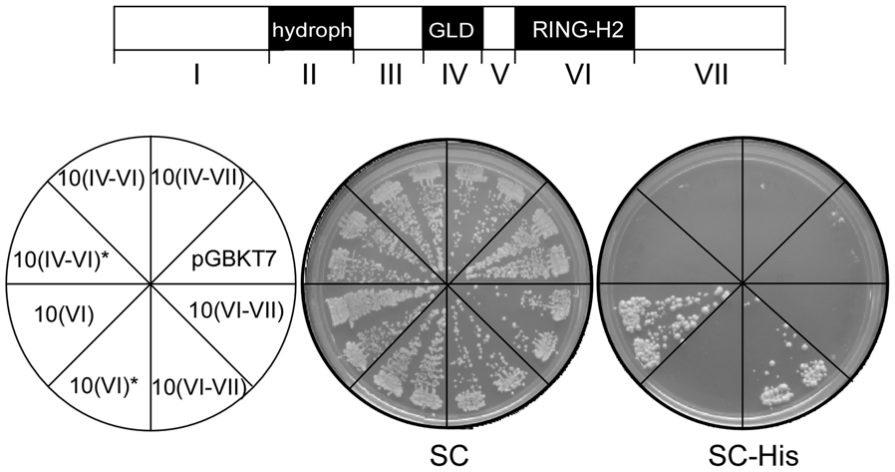
The ATL10 RING-H2 domain interacts with the ubiquitin-conjugase UBC11. Representative plates showing yeast two-hybrid interactions between UBC11 and ATL10 clones carrying the RING-H2 domain. A scheme of the seven regions in ATL10 is shown above as a guide. The left panel shows the template of the plates. ATL10-containing fragments were ligated into the DNA-binding domain vector pGBKT7 and UBC11 was recombined into the activation domain of pHOST pACT2 Lox vector; description ATL10 segments is shown in Figure 7, region IV corresponds to GLD, region VI to the RING-H2 domain and the asterisk to a mutated RING-H2 domain. The yeast two-hybrid assay was performed as in Figure 8, except that transformants were then streaked onto SC medium lacking Trp and Leu (SC), into SC medium lacking Trp, Leu, and His, and onto SC medium lacking Trp, Leu, His, and Ade. No growth was observed after 7 days of incubation on SC medium lacking Trp, Leu, His, and Ade; this plate is not shown.

Interactions between E2s and E3s are critical since they can determine the outcome of the substrate. Many of *A. thaliana* RING E3s, including ATLs function with the E2 AtUBC8, 10, 11, or 28 in auto-ubiquitination assays [5]. Likewise, an “*in vivo*” assay in yeast also showed that *A. thaliana* E2s, structurally related to the yeast *UBC4*, function with ATL2. Indeed, eight *A. thaliana* E2s, including AtUBC8, 10, 11, and 28 are related to the yeast *UBC4* [15]. We were intrigued by the fact that our yeast two-hybrid screens did not uncover any ubiquitin-conjugases. To ascertain that an interaction between E2s and the RING-H2 domain was noticeable we tested less stringent selective conditions in the yeast two-hybrid assay. As a negative control we generated and tested a RING-H2 domain carrying a point mutation in the conserved tryptophan required for the E2 binding. Under these moderate selective conditions we detected interaction between UBC11 and the RING-H2 domain but not between UBC11 and the mutated version of the RING finger (see Figure 10, compare RING and RING*). Thus, the canonical ATL RING-H2 domain and UBC11 showed moderate interaction in a yeast two-hybrid assay.

We then tested the effect of the adjoining regions of the RING-H2 domain in ATL10 (GLD and VII) on the interaction between the RING-H2 domain and UBC11. The carboxy-terminal end (region VII) has no noticeable effect on the interaction (see Figure 10, compare VI–VII and VI*–VII). Conversely, no interaction was detected between UBC11 and the RING-H2 domain that includes the GLD region, with or without region VII (see Figure X, IV–VII, IV–VI and IV–VI*). These observations suggest that GLD alters the structure of the RING-H2 bait in a manner that hampers the yeast two-hybrid assay.

## Discussion

In this study, we have continued our previous analysis on the ATL genes encoding a class of ubiquitin-ligases in plants incorporating the information from 24 complete genomes. The phylogenetic reconstruction of RING-H2 protein sequences resolved topologies with better support values than using complete protein sequences, indicating that the 42 residue RING-H2 domain harbors significant phylogenetically informative positions. Phylogenetic analysis and ATL-generated pHMM LOGOs were used in order to classify ATLs in 9 groups. The number of sequence LOGOs generated in basal species is scarce, suggesting that new motifs were acquired in superior plants. Indeed, about 44% of the sequence LOGOs were found to be group specific, suggesting that they expanded in different plant lineages. Our yeast two-hybrid analysis inferred potential domains in ATLs that correlate with sequence LOGOs. One of them mediated interaction with DSK2/ubiquilin, unraveling a possible role of this type of adapter protein in the function of ubiquitin-ligases. Our study provides instrumental information to assess a family of regulatory proteins which represent about 40% of the RING-H2 containing proteins in *A. thaliana* and do not encode previously known domains.

The ATL family is a particular class of transmembrane ubiquitin-ligases specialized in plants. A valuable feature to accurately collect ATLs from data bases was the precise and conserved structure of the RING-H2 domain, that is, the exact number of residues between the zinc ligands and specific conserved residues. Also another hallmark for this family is the presence of transmembrane helices and the GLD motif together with the RING-H2 domain in the same protein. This sequential arrangement of transmembrane and RING-H2 domains in ATL proteins suggests that they are evolutionarily related by homology. Structural analysis of protein families shows that similar domain architecture evolves from a common ancestor and tend to maintain the same functions [45], [46], [47].

Most ATLs contain a single transmembrane helix, but lineages with two or three helices are evident (see Figure 3). It is likely that depending on the type of transmembrane region an ATL protein could be targeted to a different cell compartments. RING1/ATL55, which contains a single helix, was localized to plasma membrane lipid raft subdomains [22]; ATL9, containing two transmembrane helices, was located to ER [20]; and NIP2/ATL25, with multiple transmembrane helices, was found bound to chloroplast thylakoid membranes [26]. The transmembrane region may also have evolved a subtle specialized targeting function, since 19 sequence LOGOs were generated from it that define particular groups (see Figure 5). These facts suggest that selective pressure throughout evolution has maintained such specific structures on the RING-H2 domain resulting in the specialization of a particular type of transmembrane ubiquitin-ligase in plants. Moreover a basic organization of a distinct protein domain and a modular architecture has allowed the diverse array of functions that is predicted for ATLs, such as responses to biotic stress (see Table 1).

Since their origin, the genome size of angiosperms has been diversified. Changes in environmental conditions are major selection pressures driving the variation of gene family size [48]. The availability of complete sequenced genomes from a wide range of plant species permits the comparison of the tendencies of gene distribution and expansion among them. A large variation in the number of *ATL* genes is observed among the 24 species examined in this work, being more severe in monocot than in eudicots (median, 119±16 and 64±25, respectively; data from Table S1). This variation is readily detected between the evolutionarily related species: *O. sativa* and *B. distachyon*, and *G.max* and *M. truncatula* that diverged 40–50 million years ago [49], [50], and differ by 31 and 82 ATLs, respectively.

Gene duplication events are widespread in plants, one or more whole genome duplication as well as segmental duplication events have taken place during the evolution of plant species [51], [52], [53]. Likewise, disperse gene duplication and tandem gene duplication are common events in plants [54]. Tandemly arrayed genes provide unique means to associate gene duplication with the expansion and divergence of a gene family [32]. Moreover, in plants, tandemly duplications, tend to consist of genes involved in stress response and genes coding membrane proteins [39], [40], both common features of ATLs. The number of genes per tandem array in all plant species is similar to the number previously reported for *A. thaliana*, where 70% consist of two genes, 16% of three genes, and 10% of more than three genes (see Table 2); the corresponding numbers of *A. thaliana* are 69%, 18% and 13% [55]. This observation suggests that a similar mechanism mediates this type of gene duplication event in all the analyzed plant species. Conversely, the percentages of tandemly arrayed ATLs were rather different, ranging from 3% to 36%; six species contained less than 10% (*V. vinifera*, *C. sinensis*, *C. sativus*, *M. truncatula*, *R. communis*, *M. esculenta*), and eight species more than 20% (*B. distachyon*, *O. sativa*, *S. bicolor*, *E. grandis*, *C. papaya*, *A. thaliana*, *P. persica*, *G. max*) (see Table 2). Although clusters of tandemly arrayed ATLs are present in several plant species, this observation suggests that specific sets of ATLs exhibit differential rates of expansion in different plant genomes. This observation and the fact that tandemly arrayed genes are thought to be a significant and effective niche for plant adaptation, are indicative of the mode of expansion and diversification of this gene family whose members may be involved in the regulation of stress sensing and/or the response to environmental cues [56].

Protein-protein interactions are critical for the mode of action of ubiquitin-ligases [3]. The yeast two-hybrid assay has been successfully explored to identify protein-protein interactions mediated by RING finger proteins [57], [58], [59]. Substrates of ubiquitin-ligases or interactors that are functionally associated or that stabilize the ubiquitin-ligase, but that are not necessarily substrates for the ubiquitin-ligase, have been isolated [60], [61]. The yeast two-hybrid assay was instrumental to identify interactors of ATLs and to unravel regions mediating such protein-protein interactions. Regions I and VII were identified as important regions to mediate protein-protein interactions (see Figure 7 and Table S5). These two regions showed differential sequence LOGO generation, ranging from absent or reduced to numerous in some groups. This distribution suggests that these two regions are important in conferring functional diversity to ATLs. Since a sequence LOGO represents a putative conserved motif these regions might be involved in target recognition or in mediating the interaction with components that may assist in the functioning of the ubiquitin-ligase.

Region I of several ATLs mediates the interaction with DSK2a/ubiquilin. This interaction occurs with several distantly related ATLs, some of which contain one, two or multiple hydrophobic helices; ATL10, ATL31, and ATL25, respectively. These results indicated that DSK2a/ubiquilin has a role in the mode of action of a broad number of ATLs. Interestingly, the interaction of the ATLs with DSK2a/ubiquilin seems to be mediated by the central region of the DSK2a/ubiquilin and not by UBA or UBL, the flanking domains of the protein. Similar type of interactions involving the central region of DSK2/ubiquilin have been reported. Ubiquilin-1 interacts with subunits of the neuronal nicotinic acetylcholine receptors (nAChR). Yeast two-hybrid mapping indicate that the interacting region of ubiquilin-1 comprised the STI-1-like motifs located at the central region of the protein; a role for ubiquilin-1 in regulating the assembly and trafficking of nAChR has been proposed [62]. The small hydrophobic (SH) protein of mumps virus (MuV) interacts with the ataxin-1 ubiquitin-like interacting protein (A1Up, ubiquilin-4), and this interaction is mediated by the central region of A1Up; the role of this interaction during MuV infection is unknown [63]. A complex that includes the interaction between erasin, a protein that prompts ERAD, and ubiquilin-1 has also been reported; this interaction is mediated by the central region of ubiquilin-1. The complex is localized to the ER and is suggested to participate in the regulation of ER stress [64]. The interaction between DSK2a/ubiquilin and ATLs represents a novel and important link to bring together ubiquitin-ligases, substrates, and the 26S proteasome. This interaction may impede other interactions of the UBA and UBL domains leaving them available to interact with ubiquitin (or ubiquitinylated substrates) or with the 26S proteasome, respectively. Thus in ATLs, DSK2a/ubiquilin may play a role in the regulation of protein turnover and/or as a shuttle complex for the proteasome. Region VII of ATL10 seems to mediate interaction with an array of proteins (see Table S5). Since this is the most variable region in size, ranging from few a residues to several hundreds, and shows the major number of sequence LOGO generation, it is likely that it is involved in specific substrate recognition.

The functional analysis of large gene families is difficult to approach in plants. For instance, gene redundancy and subtle phenotypic alterations resulting from mutant analysis restrict this type of analysis. The phylogenetic analysis with 24 species and the sequence LOGO generation of ATLs is the foundation to classify these types of ubiquitin-ligases and a pivotal tool for the functional characterization of this family. The functional analysis of the newly discovered protein interactors of ATLs and the pursuit of similar approaches on additional members of this family may be a way to discover the function of this type of ubiquitin-ligase.

## Materials and Methods

### Sequence Retrieval and ATL Identification

The peptide and coding sequences used in this study were retrieved from the viridiplantae genomes deposited in the Phytozome 6 database at http://www.phytozome.net/, as well as from the apple genome from the Genome Database for Rosaceae at http://www.rosaceae.org/. The genomes included two basal plants, five monocots, and seventeen eudicot plants. In the course of retrieving ATLs we detected sequences that were annotated as truncated peptides containing a RING-H2 domain of the ATL type. In some cases we were able to assemble open reading frames that showed ATL-like features; these genes were included in the list of genes to be analyzed. In other cases, genes that lacked or had truncated coding sequences for the RING finger domain were identified; these putative pseudogenes were not included in the list (data not shown).

An ATL RING-H2 HMM (Hidden Markov Model) was constructed and calibrated from 78 *A. thaliana* and 118 *O. sativa* ATL sequences. It consisted of a forty-two amino acid sequence that started at the first cysteine residue involved in metal ligation and ended at the eighth cysteine residue. It also showed a conserved distance between the eight residues involved in metal ligation, a feature that has been helpful in classifying RING finger E3s [5]. This model (CAVCLSEFEDGEKLRLLPKCGHAFHVECIDTWLRSHSTCPLC) was used as query in HMM searches to retrieve a total of 5232 non redundant sequences from 24 plant genomes [65]. From these sets we excluded 3100 sequences that did not show the canonical ATL distance conservation between metal ligands. We then predicted transmembrane helices in 1815 out of the remaining 2132 sequences by using the TMHMM Server v. 2.0, at http://www.cbs.dtu.dk/services/TMHMM/; these 1815 genes were considered ATLs. ATLs were named based on the KEGG Organism Code at http://www.genome.jp/kegg/kegg3.html (e.g. pop|POPTR_0010s24160, where pop, *Populus trichocarpa*, is the organism code and POPTR_0010s24160 is the locus name). In the case of *A. thaliana* and *O. sativa* the previous nomenclature was followed [13]. After reexamining the previously reported ATLs from *A. thaliana* and *O. sativa* [13] we realized that the prediction of gene structure for some of the members was modified. We found that nine rice proteins did not have a hydrophobic region toward the amino-terminal end and that four *O. sativa* and two *A. thaliana* proteins contained a RING-H2 domain that was not a canonical ATL RING-H2.

### Alignments and Model Test for Sequences

Protein alignments were generally performed by MUSCLE version 3.8.31 [66] and manually edited by BioEdit [67]. ProtTest was used to estimate the amino acid substitution model best fitting the ATL alignments [68]. The best model considers the relative rates of amino acid replacement and the evolutionary constraints imposed by conservation of protein structure and function.

### Architecture of ATL Proteins and Sequence Diversification

To have a comprehensive view of the members of the ATL family we divided a canonical ATL protein in seven modules. The transmembrane helix, the GLD motif and the RING-H2 domain we used as position reference. These modules are as followed: (I) from the amino-terminal end to the transmembrane helix, (II) the transmembrane helix or helices predicted by TMHMM, (III) between the transmembrane helix and the GLD motif, (IV) the GLD motif, (V) between the GLD motif and RING-H2 domain, (VI) the RING-H2 domain and (VII) from the RING-H2 domain to the carboxy-terminal end. To estimate sequence diversification in ATLs, for every protein the size of each module was determined.

### Phylogenetic analyses

The ProtTest output data file of the average parameters was used to construct NJ rooted trees with two programs: FastTree version 2.1.1 and MEGA 4; the FastTree is a maximum likelihood method while MEGA 4 is a distance matrix method [69], [70]. FastTree analysis was conducted with the default amino acid substitution matrix Jones-Taylor-Thornton (JTT), the number of rate categories of sites (gamma) was 20 and the local support values for each node was computed by resampling 1,000 times the site likelihoods and performing the Shimodaira Hasegawa test [71]. The MEGA analysis was conducted with 1000 bootstrap replicates using the JTT model and a 0.95 gamma parameter. Based on the facts that the alignment quality support may have an enormous impact on the final phylogenetic tree [38] and that in the ATL family the only region shared by all members is the RING-H2 domain, we generated trees using either complete protein sequences, concatenated motifs or the RING-H2 domain (for the RING-H2 trees the MEGA4 included the variable proportion sites equals to 0.19). The trees generated with only the RING-H2 domain or concatenated with GLD showed better resolution of plant species and more strongly supported branches (data not shown). The tree phylogenies were displayed and edited by iTOL (Interactive Tree Of Life) at http://itol.embl.de/ [72], [73]. We chose one color for each of the basal (blue), monocots (green) or eudicots (orange) plant tree branches.

### Generation of Sequence LOGOs

To search for conserved motifs in ATL proteins, MEME (Multiple EM for Motif Elicitation) version 4.4.0 was used with the following parameters: zero or one per sequence, 5 and 50 amino acids as minimum and maximum sizes of motifs. The e-value cutoff was less than e-10, due to the high variability between the ATL sequences. The MEME output analysis was loaded into MAST (Motif Alignment and Search tool) version 4.4.0 in order to remove motifs with a similarity greater than 0.75 and to search motifs into Swiss-Prot and Protein Data Bank Databases (http://www.ebi.ac.uk/uniprot and http://www.rcsb.org/pdb/home/home.do) [74]. To visualize simultaneously the phylogeny and the predicted MEME conserved motifs, we represented each ATL region with one shape symbol as follows: I, rhombus; II, down pointing pentagram; III, left pointing triangle; IV, horizontal hexagon; V, ellipse; and VI, rectangle; coloring with several different colors if there were more than one conserved motif in an ATL region. The sequence LOGOs generated were mapped to the ATL modules.

### Yeast Two-hybrid Screenings and Assays

Fragments encompassing distinct regions of ATL10|At1g49220 (regions I to VII and combinations of them, see Figure 7) were amplified by PCR from *A. thaliana* genomic DNA, and cloned into the pGBKT7 plasmid (Clontech, Mountain View, CA); these clones were verified by sequencing and used in yeast two-hybrid screenings and assays. The clones containing segments ATL10(I), ATL10(III–VII) and ATL10(VI–VII) were transformed into the *Saccharomyces cerevisiae* strain AH109 and used as bait in the yeast two-hybrid screenings (segments are diagrammatically represented in Figure 7). This assay was performed basically according to the manufacturer's instructions (MATCHMAKER GAL4 Two-Hybrid System 3, Clontech, Mountain View, CA). The AH109 strain harboring the bait clones was transformed with an A. thaliana Matchmaker cDNA library constructed in plasmid pGADT7 (Clontech, Mountain View, CA) and (-pACT library generated from mRNA isolated from 3 day-old etiolated seedlings (TAIR, stock: CD4–22). About 5x106 transformants were obtained from each experiment on medium lacking Trp and Leu; they were then selected and consecutively tested for His3 and Ade2 reporter gene activation. Plasmid DNA was recovered from His3, Ade2 positive yeast colonies and transformed into Escherichia coli strain DH5alpha. Plasmid DNA was then isolated and used to transform AH109 and AH109 harboring the bait clones. Inserts from clones that did not show self-activation and showed activation of His3 and Ade2 reporter genes were sequenced and were considered as positive clones.

To map regions in ATL10 mediating the interaction with the positive clones, they were cotransformed into AH109 with clones containing regions VI–VII and VII, along with empty vectors as controls. Likewise to assess the specificity of ATL10 interactions, clones containing region VII from ATL76 and ATL78 were cotransformed. In all cases, activation of His3 and Ade2 reporter genes was then assessed (high-stringency conditions).

To map the region in DSK2a/ubiquilin mediating the interaction with ATL10(I), fragments encompassing the UBA or UBL domains or the central region of the protein (clone ΔUBLΔUBA), as well as the full length coding region were amplified by PCR from clone G09422 (obtained from ABRC) and cloned in the pGADT7 vector (Clontech, Mountain View, CA); clones were verified by sequencing. Yeast two-hybrid interactions were assayed for His3 and Ade2 reporter gene activation on medium lacking Trp and Leu (high-stringency conditions). To evaluate the interaction between DSK2a/ubiquilin and different ATLs, a fragment including region I and a small part of region II from genes ATL78|At1g49230, ATL2|At3g16720, ATL25| At2g17730, ATL31|At5g27420, and ATL73|At5g05280 were obtained from *A. thaliana* genomic DNA. Fragments were then cloned into the pGBKT7 vector and the constructs were verified by sequencing. The resulting clones were cotransformed into AH109 harboring the ΔUBL or the ΔUBLΔUBA clone; activation of His3 and Ade2 reporter genes was then assessed (high-stringency conditions).

To determine the interaction between the RING finger domain and ubiquitin-conjugases, the AtUBC11 (At3g08690) clone U18004, was transferred by cre/lox mediated recombination into the pHOST pACT2 Lox vector which contains the GAL4 activation domain (U18004 and pACT2 Lox were obtained from ABRC) [75]. The resulting UBC11 clone was cotransformed into AH109 harboring pGBKT7 clones that contained ATL10(IV–VII), ATL10(VI–VII), ATL10(IV–VI), ATL10(VI), ATL10(IV–VII)*, ATL10(IV–VI)*, ATL10(VI)* (see Figure 7). Region VI corresponds to the RING-H2 finger domain, the asterisk indicates a mutated RING-H2 domain where the invariable tryptophan residue was replaced by an alanine residue. Mutagenesis was performed by the GeneTailor site directed mutagenesis system (Invitrogen, Carlsbad, CA). Activation of the His3 reporter gene was then assessed (moderate-stringency conditions), since under His3 and Ade2 selection (high-stringency conditions) no growth was observed, even after seven days of incubation at 30°C.

## Supporting Information

Figure S1 **Phylogenetic tree of ATLs from 24 genomes**. The phylogeny was built using FasTree, the likelihood-based local support values above 80% is depicted by dots and the domain architecture based on sequence LOGOs is depicted in each branch.
(PDF)

Figure S2 **Divergent Tandemly arrayed ATLs.** Chromosomal location and domain architecture based on sequence LOGOs of pairs of tandemly arrayed *ATLs* that are not in the same monophyletic group. A, pairs positioned in groups D and H; B, pairs positioned in groups D and G; C, a pair positioned in groups H and F.
(TIF)

Table S1 **Number of genes containing a RING-H2 domain or a canonical ATL RING-H2 domain in 24 plant genomes.** Classification, species name and the abbreviation for each of the 24 plant species used in this work is displayed.
(PDF)

Table S2 **Distribution in 9 groups of ATLs retrieved from 24 plant species.** tm, number of transmembrane helices predicted by the TMHMM Server v. 2.0.
(PDF)

Table S3 **Catalog of 75 sequence LOGOs generated from 1815 ATLs.**
(PDF)

Table S4 **Tandemly arrayed** ***ATL*** **genes.** Genes retrieved from 23 plant species. General information and motif architecture is displayed.
(PDF)

Table S5 **ATL10 interactors mapped to region VII.**
(PDF)
