# Supplementary figures and images for: Diversity in the Architecture of ATLs, a Family of Plant Ubiquitin-Ligases, Leads to Recognition and Targeting of Substrates in Different Cellular Environments
Source: PLoS One. 2011 Aug 24;6(8):e23934. doi: 10.1371/journal.pone.0023934 (PMC3161093; doi:10.1371/journal.pone.0023934)

# Figure S1

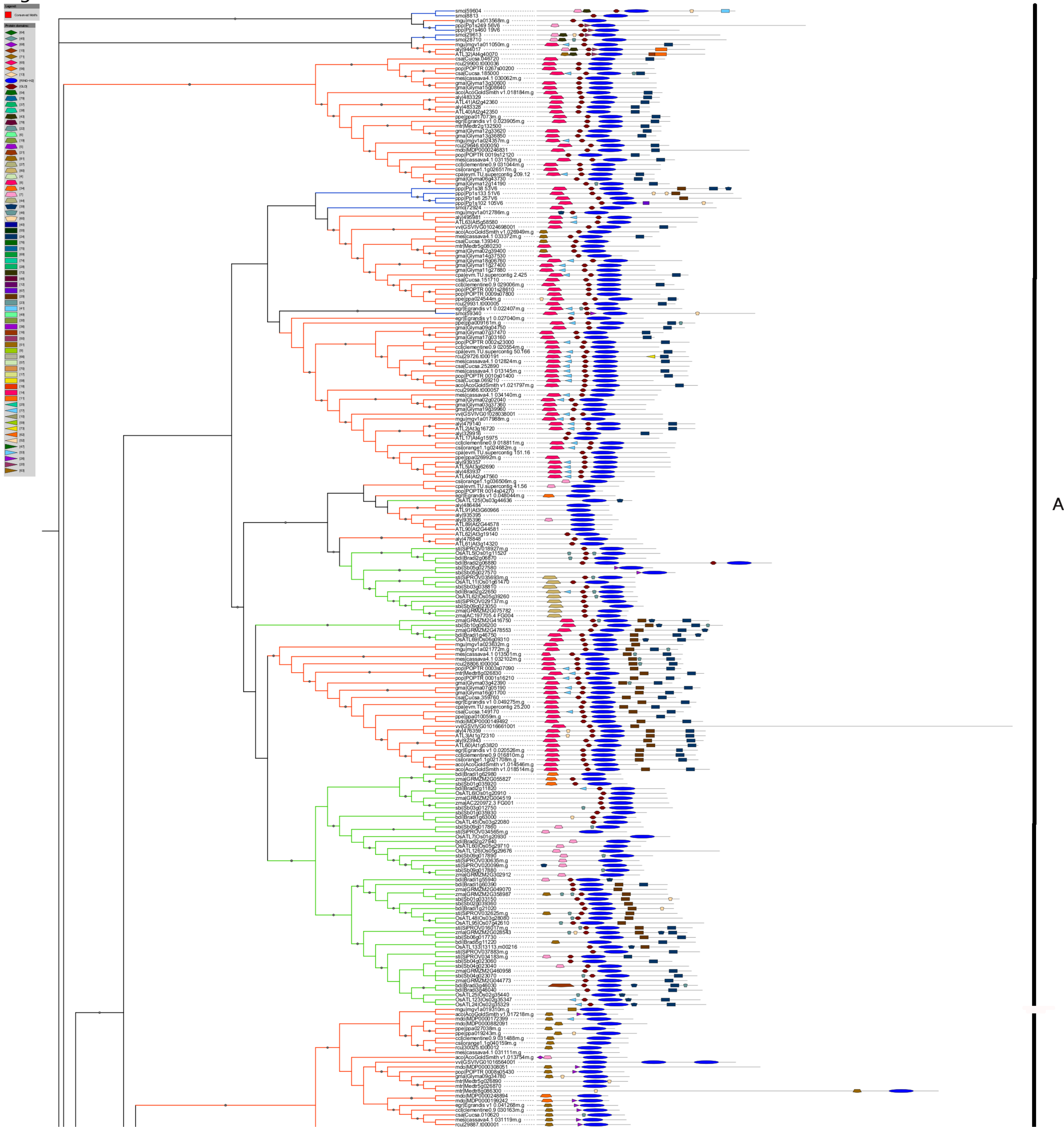

A

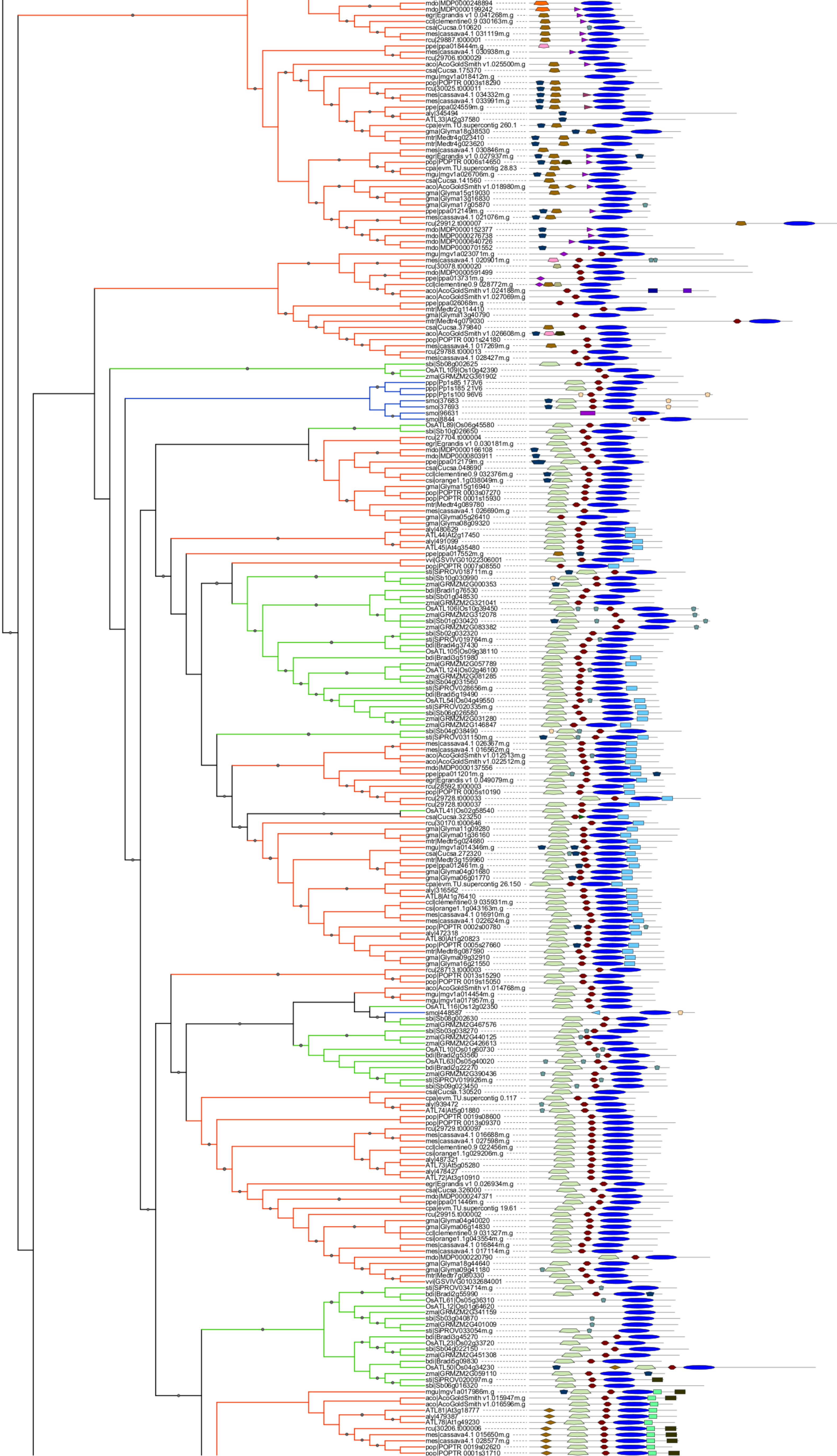

B

C

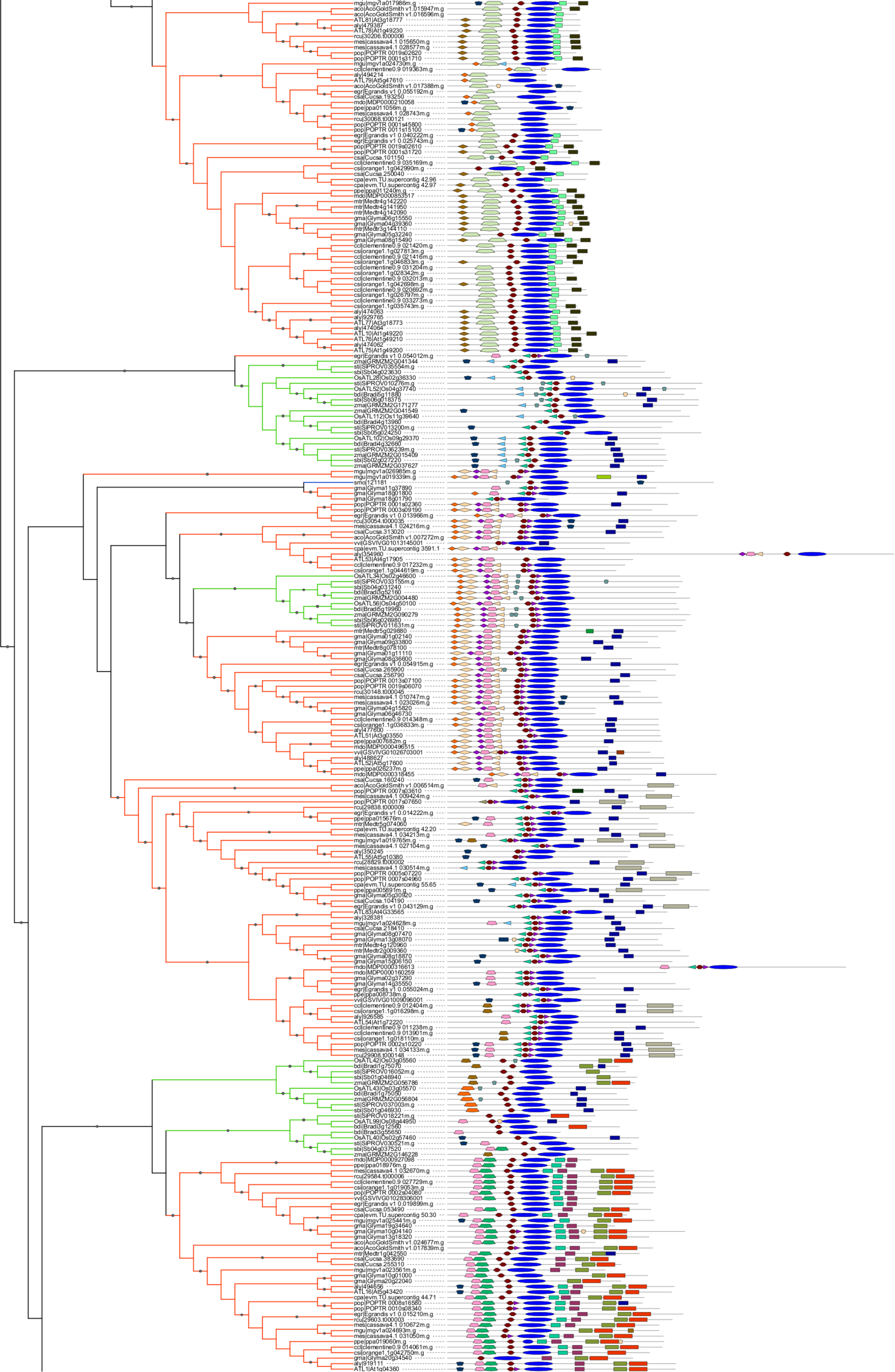

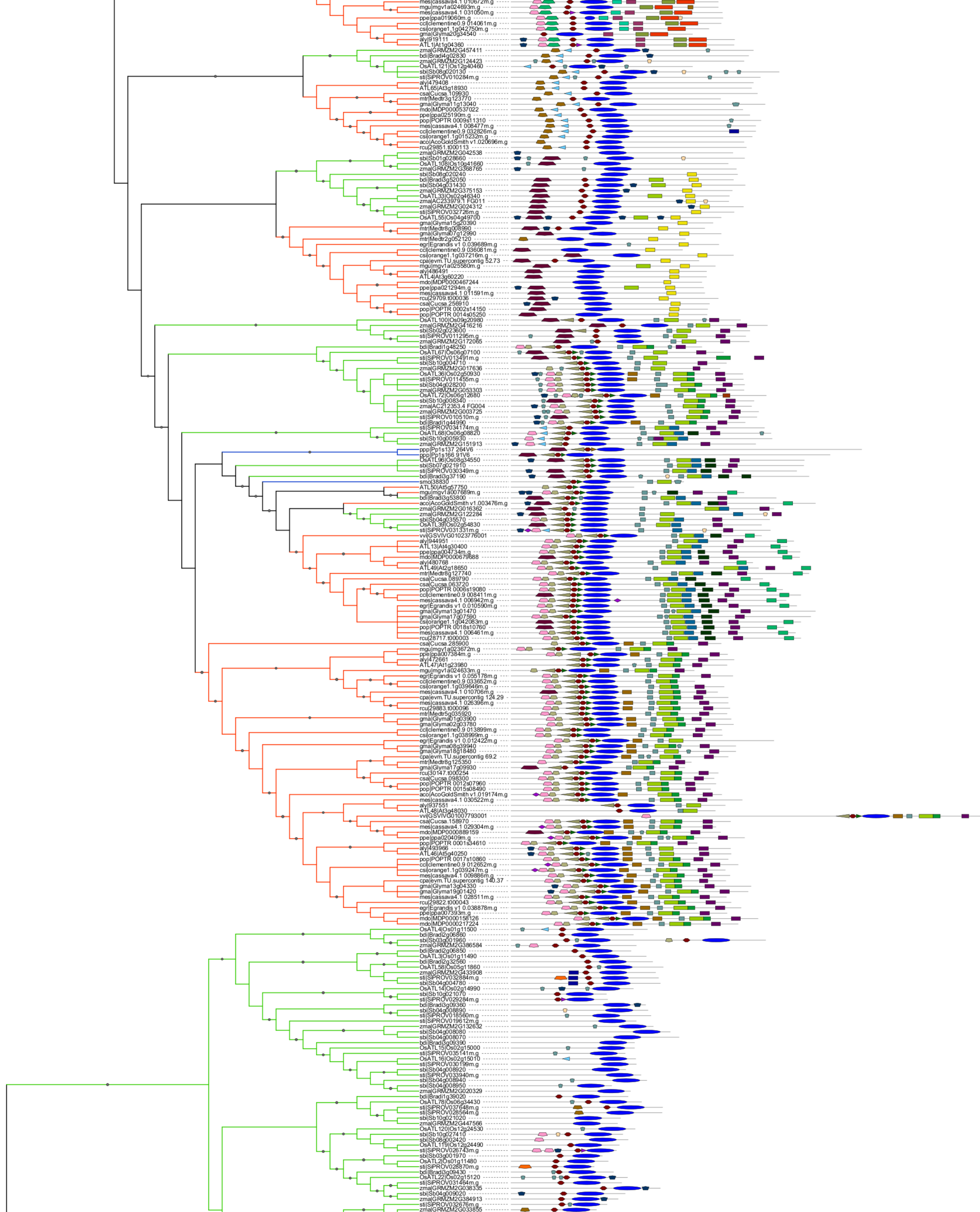

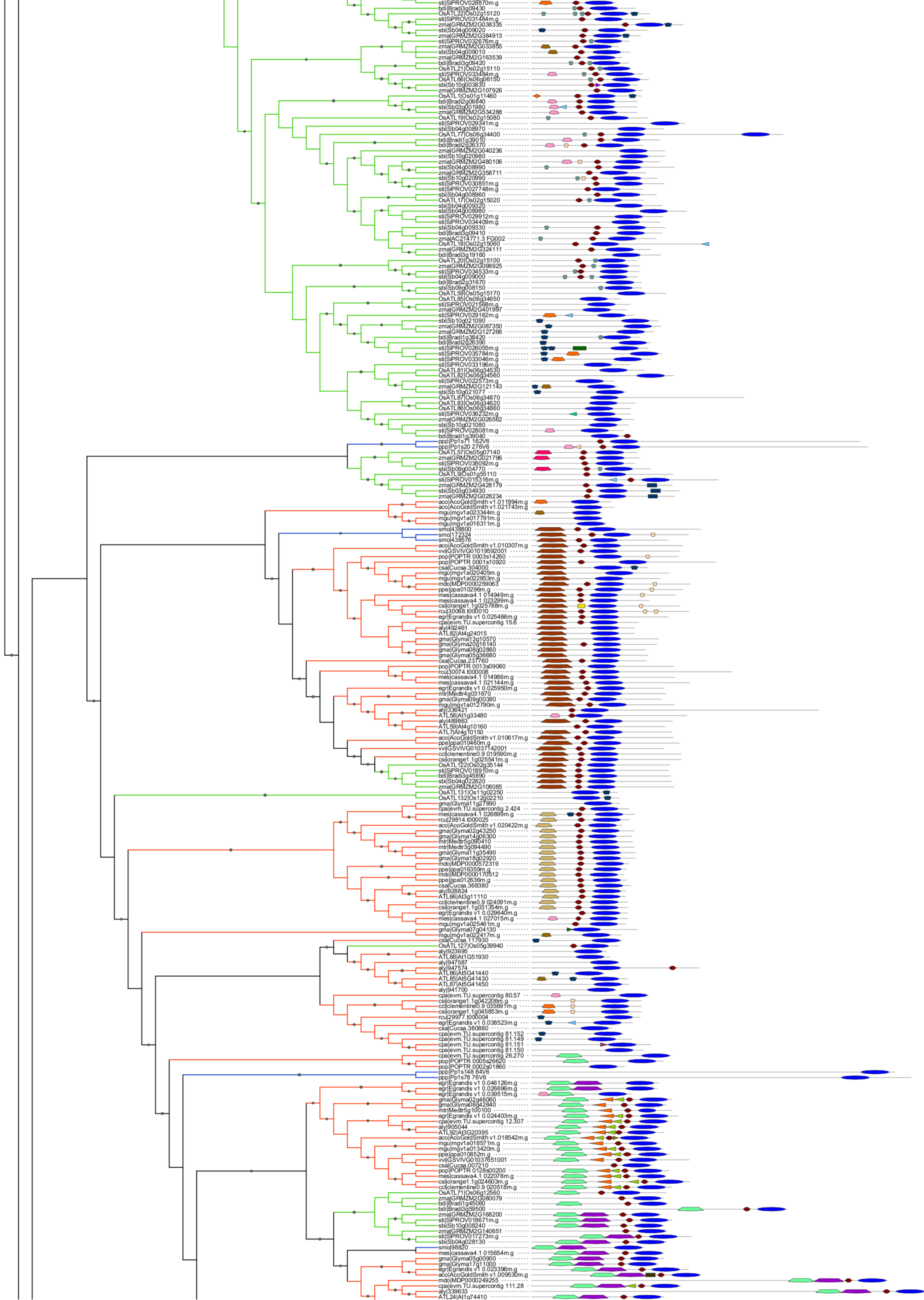

E

F

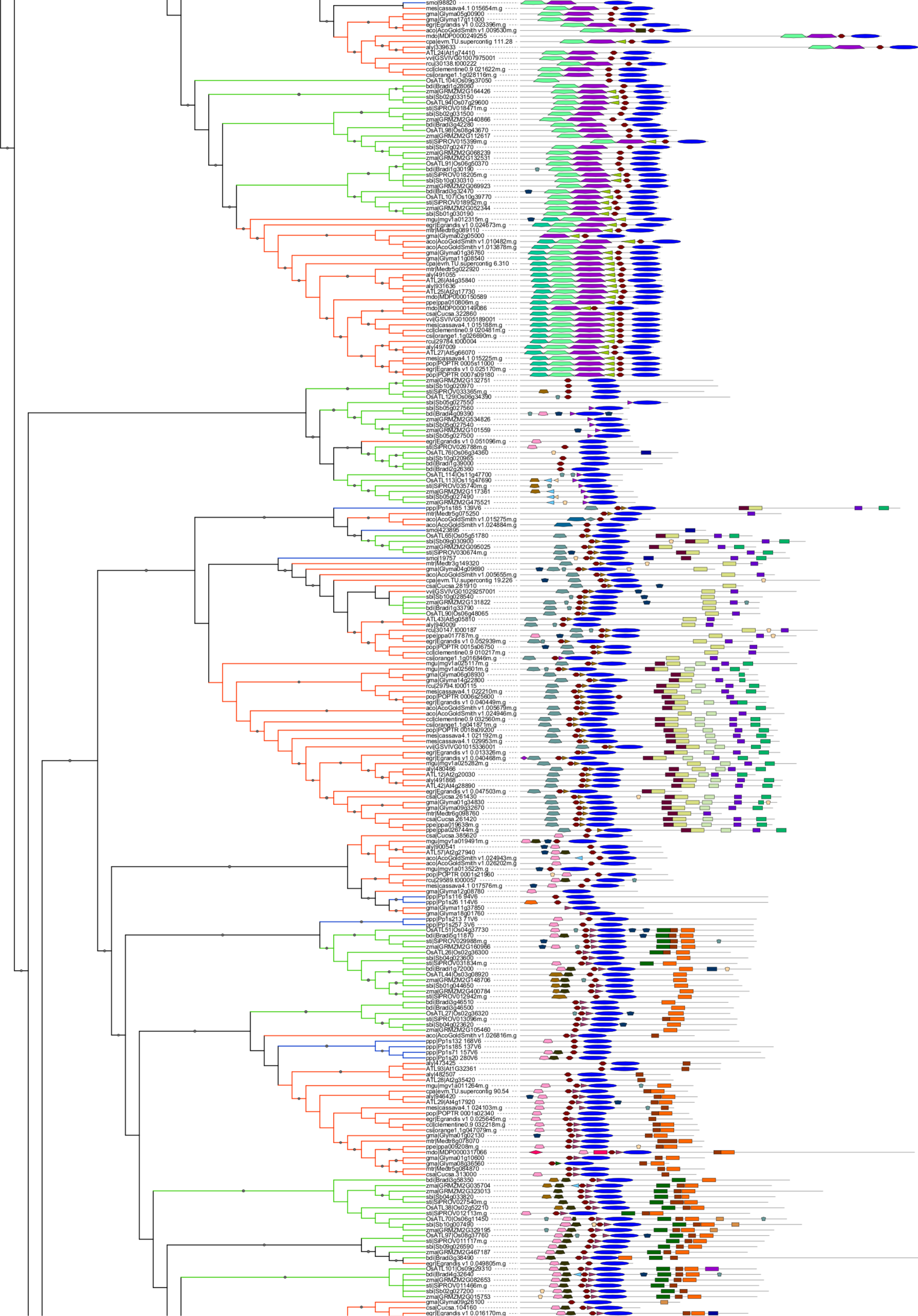

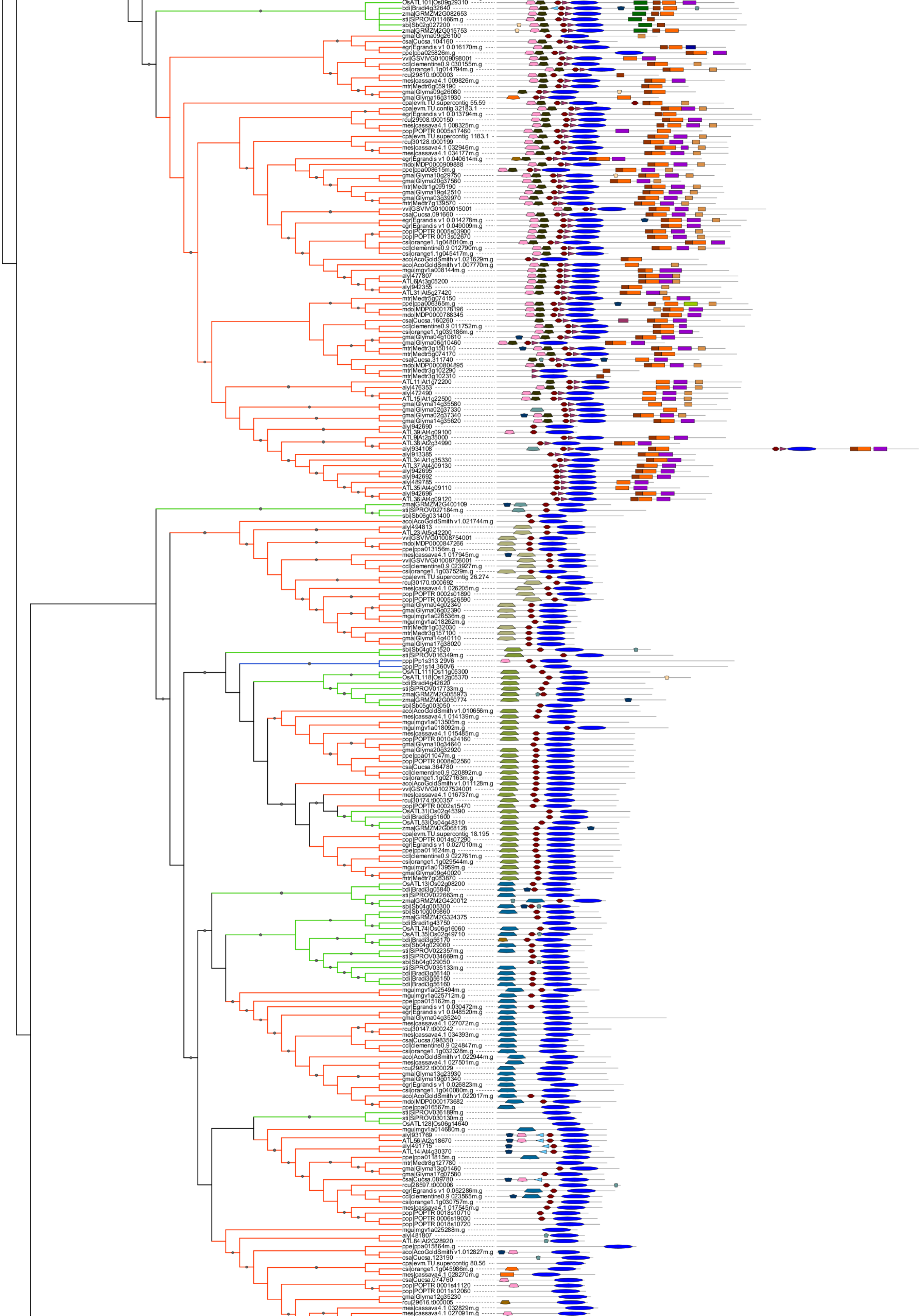

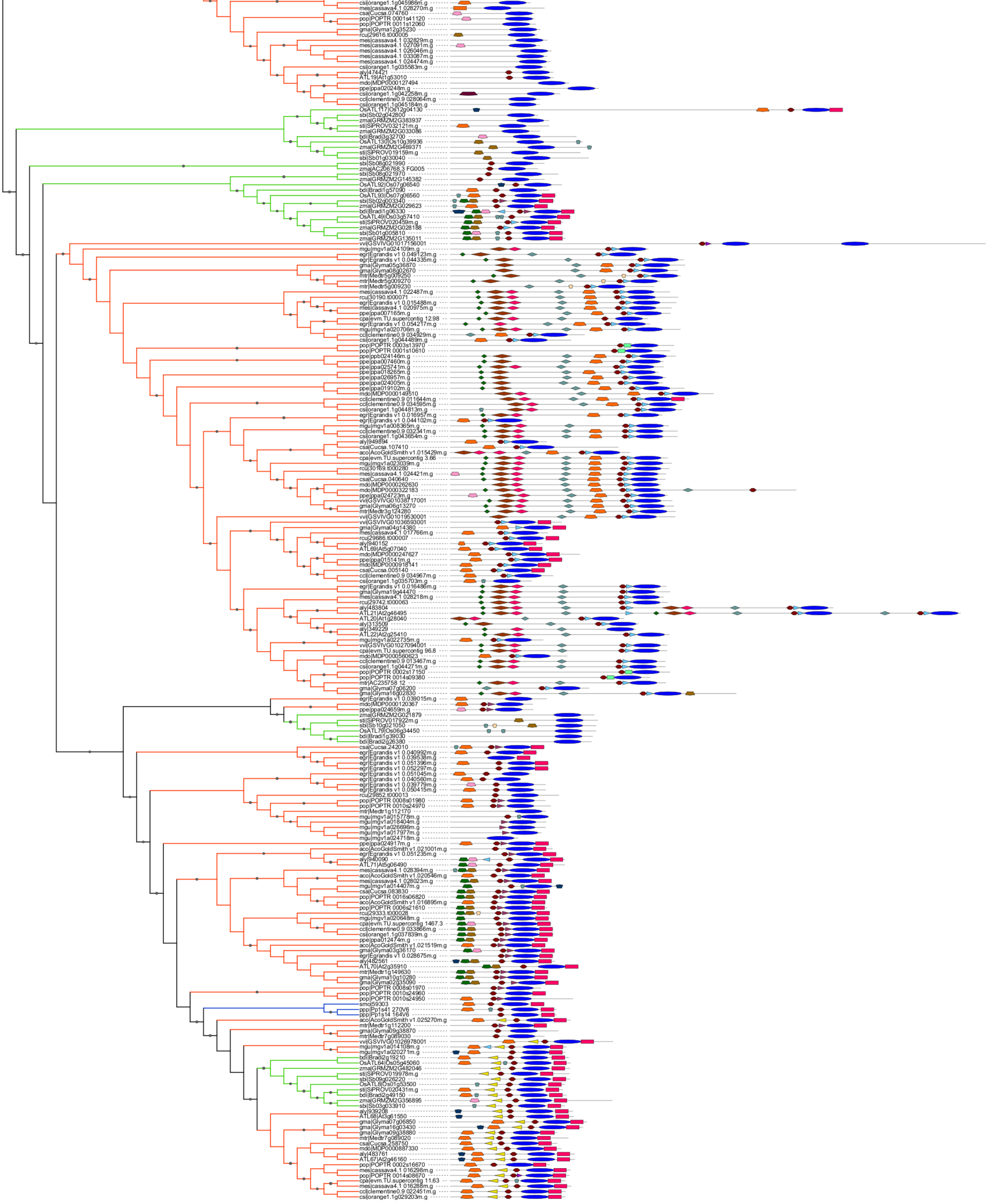

Supplement: Figure S1 — Phylogenetic tree of ATLs from 24 genomes. The phylogeny was built using FasTree, the likelihood-based local support values above 80% is depicted by dots and the domain architecture based on sequence LOGOs is depicted in each branch. (PDF) [file pone.0023934.s001.pdf]

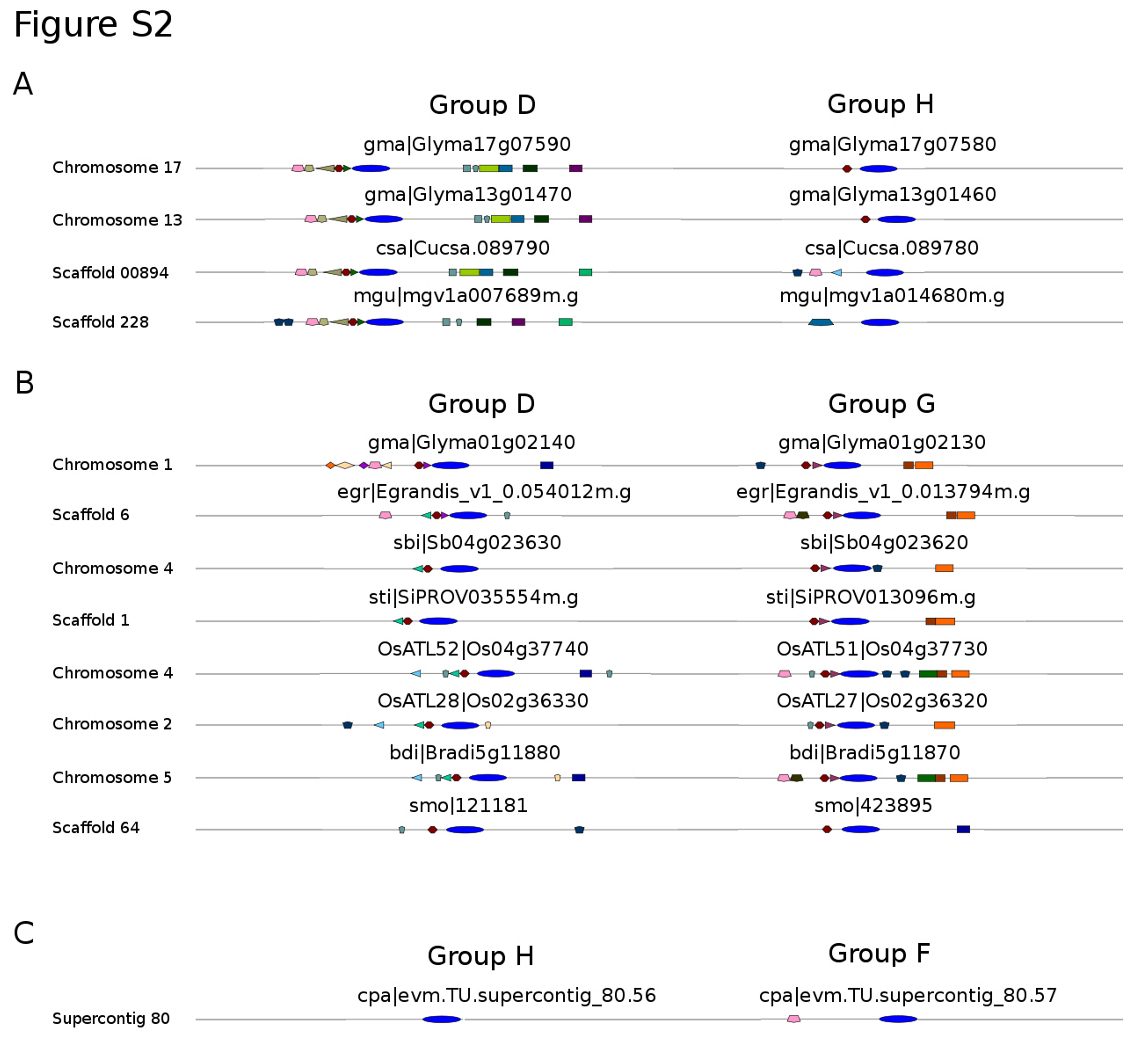

Supplement: Figure S2 — Divergent Tandemly arrayed ATLs. Chromosomal location and domain architecture based on sequence LOGOs of pairs of tandemly arrayed ATLs that are not in the same monophyletic group. A, pairs positioned in groups D and H; B, pairs positioned in groups D and G; C, a pair positioned in groups H and F. (TIF) [file pone.0023934.s002.tif]

**Table S3. Catalog of 75 sequence LOGOs generated from 1815 ATs.**

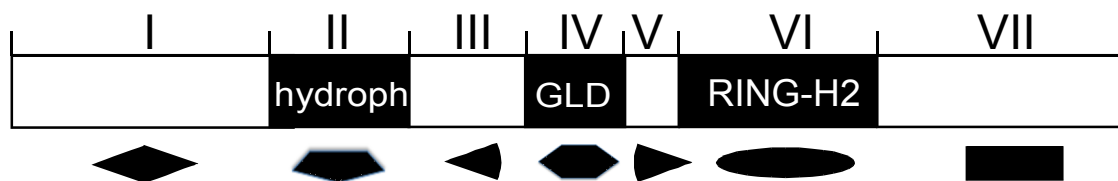

| LOGO number | Symbol | Sequence |
|-------------|--------|----------|
| [13]        |        |          |
| [15]        |        |          |
| [45]        |        |          |
| [56]        |        |          |
| [64]        |        |          |
| [65]        |        |          |
| [68]        |        |          |
| [71]        |        |          |
| [4]         |        |          |
| [5]         |        |          |
| [6]         |        |          |
| [7]         |        |          |
| [8]         |        |          |
| [19]        |        |          |
| [21]        |        |          |

|      |                                                                                     |                                                                                      |
|------|-------------------------------------------------------------------------------------|--------------------------------------------------------------------------------------|
| [22] | 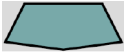   | 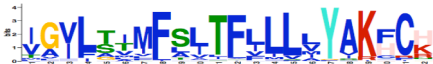   |
| [27] | 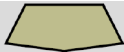   | 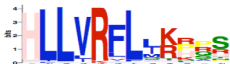   |
| [34] | 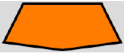   | 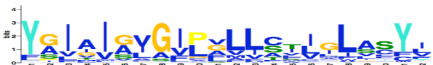   |
| [37] | 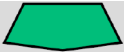   | 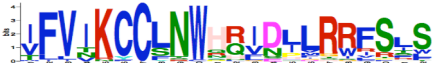   |
| [38] | 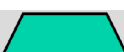   | 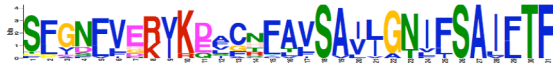   |
| [43] | 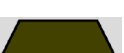   | 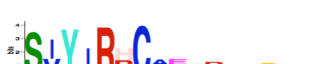   |
| [44] | 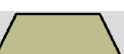   | 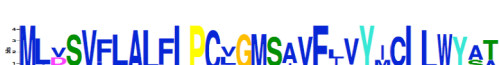   |
| [54] | 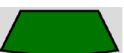   | 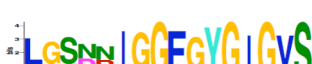   |
| [78] | 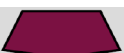   | 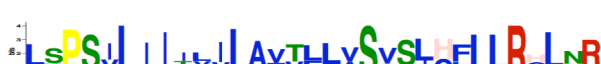   |
| [79] | 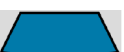  | 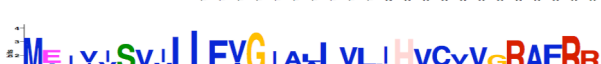  |
| [80] | 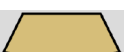 | 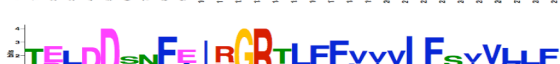 |
| [81] | 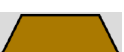 | 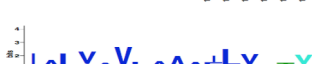 |
| [10] | 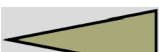 | 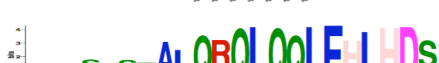 |
| [25] | 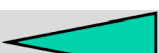 | 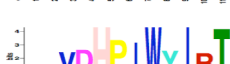 |
| [52] | 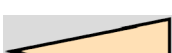 | 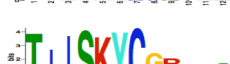 |
| [59] | 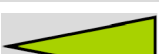 | 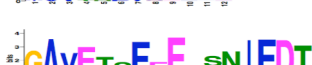 |
| [62] | 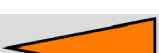 | 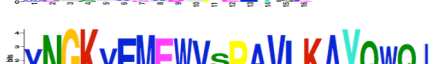 |
| [73] | 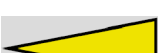 | 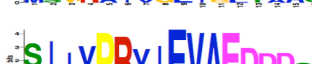 |
| [77] | 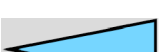 | 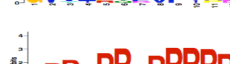 |
| [3]  | 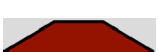 | 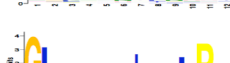 |
| [20] | 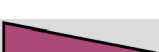 | 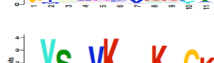 |
| [26] | 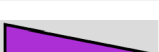 | 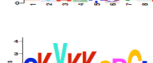  |

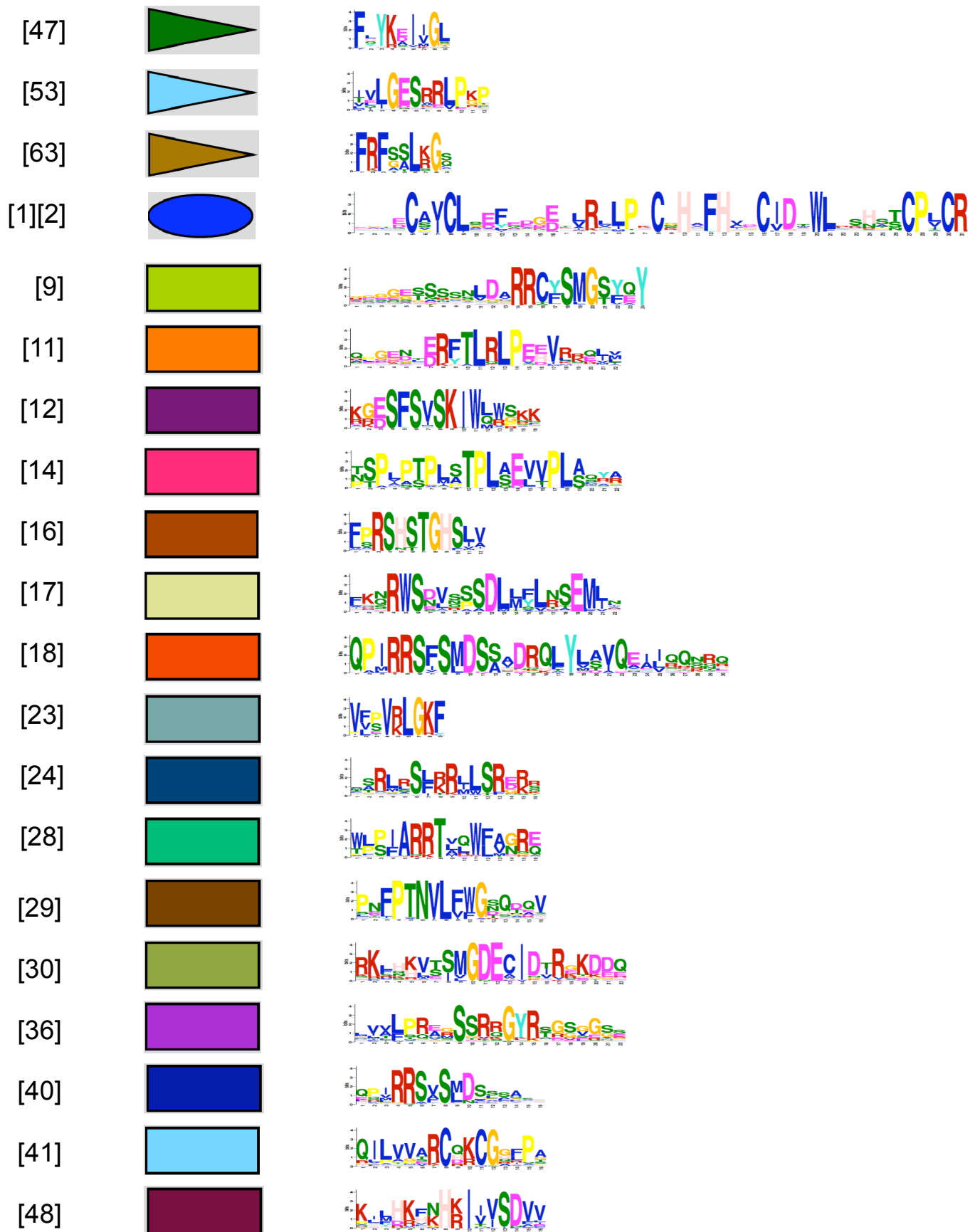

|      |                                                                                     |                                                                                      |
|------|-------------------------------------------------------------------------------------|--------------------------------------------------------------------------------------|
| [49] | 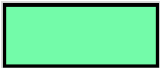   | 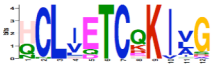    |
| [50] | 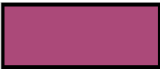   | 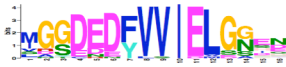   |
| [51] | 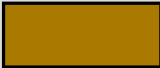   | 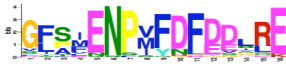   |
| [55] | 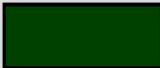   | 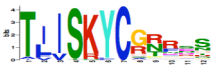    |
| [57] | 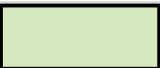   | 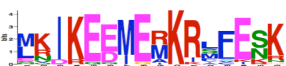   |
| [58] | 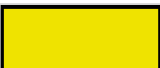   | 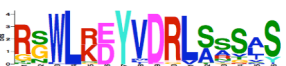   |
| [66] | 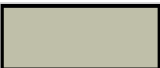   | 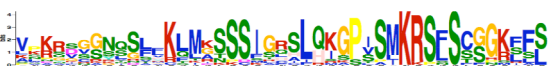   |
| [67] | 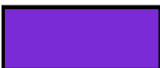   | 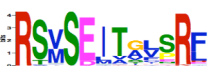    |
| [69] | 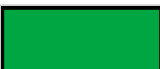   | 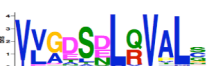    |
| [70] | 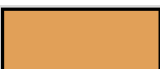 | 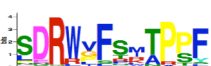  |
| [72] | 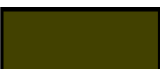 | 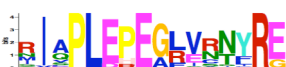 |
| [74] | 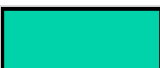 | 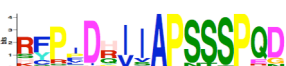 |
| [75] | 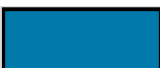 | 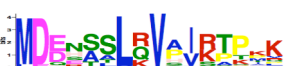 |
| [76] | 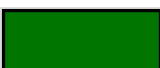 | 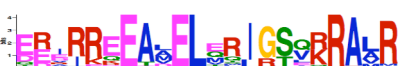 |
| [39] | 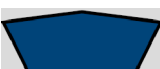 | 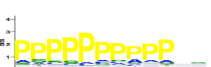  |
| [46] | 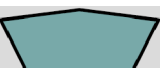 | 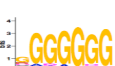  |
| [60] | 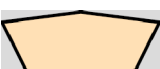 | 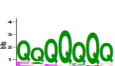  |

Supplement: Table S3 — Catalog of 75 sequence LOGOs generated from 1815 ATLs. (PDF) [file pone.0023934.s005.pdf]
