# Supplementary material for: Diversity in the Architecture of ATLs, a Family of Plant Ubiquitin-Ligases, Leads to Recognition and Targeting of Substrates in Different Cellular Environments
Source: PLoS One. 2011 Aug 24;6(8):e23934. doi: 10.1371/journal.pone.0023934 (PMC3161093; doi:10.1371/journal.pone.0023934)
Supplement: Table S1 — Number of genes containing a RING-H2 domain or a canonical ATL RING-H2 domain in 24 plant genomes. Classification, species name and the abbreviation for each of the 24 plant species used in this work is displayed. (PDF) [file pone.0023934.s003.pdf]

Table S1 Number of genes containing a RING-H2 domain or a canonical ATL RING-H2 domain in 24 plant genomes.

	Specie	Abbreviation	RING-H2*	Canonical ATL RING-H2**	Canonical ATL RING-H2/TM***
Bryophyta	*Physcomitrella patens* (moss)	ppp	87	31	28
Lycophyta	*Selaginella moellendorfii* (spikemoss)	smo	73	31	20
Monocots	*Oryza sativa* japonica (japanese rice)	osa	263	126	119
	Brachypodium distachyon (urple false brome)	bdi	281	97	88
	Setaria italica (foxtail millet)	sit	270	116	103
	Zea mays (maize)	zma	302	140	126
	Sorghum bicolor (sorghum)	sbi	254	125	124
Eudicots	*Aquilegia_coerulea*	aco	178	65	56
	Mimulus guttatus (monkey flower)	mgu	235	89	68
	Vitis vinifera (wine grape)	vvi	117	39	30
	Eucalyptus grandis (flooded Gum)	egr	272	99	74
	Citrus clementina (clementine)	ccl	214	74	59
	Citrus sinensis (sweet orange)	csi	250	89	60
	Carica papaya (papaya)	cpa	136	54	46
	Arabidopsis lyrata (lyrate rockcress)	aly	232	97	88
	Arabidopsis thaliana (thale cress)	ath	247	97	91
	Cucumis sativus (cucumber)	csa	154	67	63
	Medicago truncatula (barrel medic)	mtr	178	69	64
	Glycine max (soybean)	gma	351	162	146
	Prunus persica (peach)	ppe	180	78	66
	Malus x domestica (apple)	mdo	317	113	52
	Populus trichocarpa (black cottonwood)	pop	247	104	90
	Ricinus communis (castor bean)	rcu	156	66	60
	Manihot esculenta (cassava)	mes	238	104	94
		Number of genes	5232	2132	1815

*Number of genes encoding a RING-H2 domain
**Number of genes encoding a canonical ATL RING-H2 domain
***Number of genes encoding a canonical ATL RING-H2 domain and a transmembrane helix
