# Supplementary material for: Diversity in the Architecture of ATLs, a Family of Plant Ubiquitin-Ligases, Leads to Recognition and Targeting of Substrates in Different Cellular Environments
Source: PLoS One. 2011 Aug 24;6(8):e23934. doi: 10.1371/journal.pone.0023934 (PMC3161093; doi:10.1371/journal.pone.0023934)
Supplement: Table S2 — Distribution in 9 groups of ATLs retrieved from 24 plant species. tm, number of transmembrane helices predicted by the TMHMM Server v. 2.0. (PDF) [file pone.0023934.s004.pdf]

Table S2 Distribution in 9 groups of ATLs retrieved from 24 plant species

	Group																	
plant	A	tm	B	tm	C	tm	D	tm	E	tm	F	tm	G	tm	H	tm	I	tm
ppp	ppp|Pp1s102_105V6	1			ppp|Pp1s100_96V6	1	ppp|Pp1s137_264V6	1			ppp|Pp1s148_84V6	1	ppp|Pp1s116_94V6	1	ppp|Pp1s14_360V6	1	ppp|Pp1s14_164V6	1
	ppp|Pp1s133_51V6	1			ppp|Pp1s185_21V6	1	ppp|Pp1s166_91V6	1			ppp|Pp1s20_276V6	1	ppp|Pp1s132_168V6	1	ppp|Pp1s313_29V6	1	ppp|Pp1s41_270V6	1
	ppp|Pp1s249_56V6	1			ppp|Pp1s85_173V6	1					ppp|Pp1s71_162V6	1	ppp|Pp1s185_137V6	1				
	ppp|Pp1s38_53V6	1									ppp|Pp1s78_76V6	1	ppp|Pp1s20_280V6	1				
	ppp|Pp1s460_19V6	1											ppp|Pp1s213_71V6	1				
	ppp|Pp1s6_257V6	1											ppp|Pp1s257_3V6	1				
													ppp|Pp1s26_114V6	1				
													ppp|Pp1s71_157V6	1				
													ppp|Pp1s185_139V6	2				
smo	smo|28710	1			smo|37683	1	smo|121181	1			smo|438576	1	smo|19757	1			smo|59303	1
	smo|29613	1			smo|37693	1	smo|38830	1			smo|438800	1	smo|423895	1				
	smo|59340	1			smo|448587	1					smo|98820	1						
	smo|59604	1			smo|8844	1					smo|172324	2						
	smo|72924	1			smo|96631	1												
	smo|8813	1																
osa	OsATL11|Os01g61470	1			OsATL10|Os01g60730	1	OsATL100|Os09g20980	1	OsATL1|Os01g11460	1	OsATL107|Os10g39770	1	OsATL113|Os11g47690	1	OsATL111|Os11g05300	1	OsATL117|Os12g04130	1
	OsATL123|Os02g35347	1			OsATL105|Os09g38110	1	OsATL102|Os09g29370	1	OsATL14|Os02g14990	1	OsATL122|Os02g35144	1	OsATL114|Os11g47700	1	OsATL118|Os12g05370	1	OsATL49|Os03g57410	1
	OsATL125|Os03g44636	1			OsATL106|Os10g39450	1	OsATL108|Os10g41660	1	OsATL17|Os02g15020	1	OsATL127|Os05g39940	1	OsATL129|Os06g34390	1	OsATL128|Os06g14640	1	OsATL64|Os05g45060	1
	OsATL126|Os05g29676	1			OsATL109|Os10g42390	1	OsATL112|Os11g39640	1	OsATL18|Os02g15060	1	OsATL131|Os11g02250	1	OsATL65|Os05g51780	1	OsATL13|Os02g08200	1	OsATL8|Os01g53500	1
	OsATL133|13113.m00216	1			OsATL12|Os01g64620	1	OsATL121|Os12g40460	1	OsATL2|Os01g11480	1	OsATL132|Os12g02210	1	OsATL76|Os06g34360	1	OsATL31|Os02g45390	1	OsATL92|Os07g06540	1
	OsATL24|Os02g35329	1			OsATL124|Os02g46100	1	OsATL28|Os02g36330	1	OsATL21|Os02g15110	1	OsATL57|Os05g07140	1	OsATL90|Os06g48065	1	OsATL35|Os02g49710	1	OsATL93|Os07g06560	1
	OsATL25|Os02g35440	1			OsATL23|Os02g33720	1	OsATL33|Os02g46340	1	OsATL22|Os02g15120	1	OsATL71|Os06g12560	1	OsATL101|Os09g29310	2	OsATL53|Os04g48310	1	OsATL130|Os10g39936	2
	OsATL45|Os03g22080	1			OsATL41|Os02g58540	1	OsATL34|Os02g46600	1	OsATL3|Os01g11490	1	OsATL9|Os01g55110	1	OsATL26|Os02g36300	2	OsATL74|Os06g16060	1	OsATL79|Os06g34450	2
	OsATL48|Os03g28080	1			OsATL50|Os04g34230	1	OsATL36|Os02g50930	1	OsATL4|Os01g11500	1	OsATL91|Os06g50370	1	OsATL27|Os02g36320	2				
	OsATL5|Os01g11520	1			OsATL54|Os04g49550	1	OsATL39|Os02g54830	1	OsATL58|Os05g11860	1	OsATL94|Os07g29600	1	OsATL38|Os02g52210	2				
	OsATL6|Os01g20910	1			OsATL63|Os05g40020	1	OsATL40|Os02g57460	1	OsATL59|Os05g15170	1	OsATL98|Os08g43670	1	OsATL44|Os03g08920	2				
	OsATL60|Os05g29710	1			OsATL89|Os06g45580	1	OsATL42|Os03g05560	1	OsATL77|Os06g34400	1	OsATL104|Os09g37050	2	OsATL51|Os04g37730	2				
	OsATL62|Os05g39260	1			OsATL116|Os12g02350	2	OsATL43|Os03g05570	1	OsATL78|Os06g34430	1			OsATL70|Os06g11450	2				
	OsATL69|Os06g09310	1			OsATL61|Os05g36310	2	OsATL52|Os04g37740	1	OsATL81|Os06g34530	1			OsATL97|Os08g37760	2				
	OsATL7|Os01g20930	1					OsATL55|Os04g49700	1	OsATL82|Os06g34560	1								
	OsATL95|Os07g42610	2					OsATL56|Os04g50100	1	OsATL83|Os06g34620	1								
							OsATL67|Os06g07100	1	OsATL85|Os06g34650	1								
							OsATL68|Os06g08820	1	OsATL86|Os06g34860	1								
							OsATL72|Os06g12680	1	OsATL87|Os06g34870	1								
							OsATL96|Os08g34550	1	OsATL119|Os12g24490	2								
							OsATL99|Os08g44950	1	OsATL120|Os12g24530	2								
									OsATL15|Os02g15000	2								
									OsATL16|Os02g15010	2								
									OsATL19|Os02g15080	2								
									OsATL20|Os02g15100	2								
									OsATL66|Os06g06150	2								
bdi	bdi|Bradi1g21020	1			bdi|Bradi1g76530	1	bdi|Bradi1g44990	1	bdi|Bradi1g38420	1	bdi|Bradi1g28060	1	bdi|Bradi1g33790	1	bdi|Bradi1g43750	1	bdi|Bradi1g06330	1
	bdi|Bradi1g46750	1			bdi|Bradi2g22270	1	bdi|Bradi1g48250	1	bdi|Bradi1g39040	1	bdi|Bradi1g45060	1	bdi|Bradi1g39000	1	bdi|Bradi3g05840	1	bdi|Bradi1g57090	1
	bdi|Bradi1g55940	1			bdi|Bradi2g53560	1	bdi|Bradi1g75050	1	bdi|Bradi2g06840	1	bdi|Bradi3g32470	1	bdi|Bradi2g26360	1	bdi|Bradi3g51600	1	bdi|Bradi2g19210	1

	bdi|Bradi1g60390	1	bdi|Bradi2g55990	1	bdi|Bradi1g75070	1	bdi|Bradi2g06850	1	bdi|Bradi3g42280	1	bdi|Bradi3g46510	1	bdi|Bradi3g56140	1	bdi|Bradi2g49150	1
	bdi|Bradi1g62980	1	bdi|Bradi3g45270	1	bdi|Bradi3g12560	1	bdi|Bradi2g06860	1	bdi|Bradi3g45890	1	bdi|Bradi3g58350	1	bdi|Bradi3g56150	1	bdi|Bradi3g32700	1
	bdi|Bradi1g63000	1	bdi|Bradi3g51980	1	bdi|Bradi3g37190	1	bdi|Bradi2g26390	1	bdi|Bradi3g59500	1	bdi|Bradi4g09390	1	bdi|Bradi3g56160	1	bdi|Bradi1g39030	3
	bdi|Bradi2g06870	1	bdi|Bradi4g37430	1	bdi|Bradi3g52050	1	bdi|Bradi2g31670	1	bdi|Bradi1g30190	3	bdi|Bradi1g72000	2	bdi|Bradi3g56170	1	bdi|Bradi2g26380	3
	bdi|Bradi2g11820	1	bdi|Bradi5g09830	1	bdi|Bradi3g52160	1	bdi|Bradi2g32560	1			bdi|Bradi3g38490	2	bdi|Bradi4g42620	1		
	bdi|Bradi2g22650	1	bdi|Bradi5g19490	1	bdi|Bradi3g53800	1	bdi|Bradi3g09410	1			bdi|Bradi3g46500	2				
	bdi|Bradi2g27940	1			bdi|Bradi3g55650	1	bdi|Bradi3g09420	1			bdi|Bradi4g32640	2				
	bdi|Bradi3g46030	1			bdi|Bradi4g13960	1	bdi|Bradi3g09430	1			bdi|Bradi5g11870	2				
	bdi|Bradi3g46040	1			bdi|Bradi4g32660	1	bdi|Bradi1g39010	2								
	bdi|Bradi5g11220	1			bdi|Bradi5g11880	1	bdi|Bradi1g39020	2								
	bdi|Bradi2g06880	2			bdi|Bradi5g19960	1	bdi|Bradi2g26370	2								
					bdi|Bradi4g02830	2	bdi|Bradi3g09360	2								
							bdi|Bradi3g09390	2								
							bdi|Bradi3g19160	2								
sti	sti|SiPROV016017m.g	1	sti|SiPROV018711m.g	1	sti|SiPROV010276m.g	1	sti|SiPROV021568m.g	1	sti|SiPROV015316m.g	1	sti|SiPROV012113m.g	1	sti|SiPROV016349m.g	1	sti|SiPROV019159m.g	1
	sti|SiPROV018927m.g	1	sti|SiPROV019764m.g	1	sti|SiPROV010284m.g	1	sti|SiPROV022573m.g	1	sti|SiPROV015399m.g	1	sti|SiPROV012942m.g	1	sti|SiPROV017733m.g	1	sti|SiPROV019978m.g	1
	sti|SiPROV020099m.g	1	sti|SiPROV019926m.g	1	sti|SiPROV010510m.g	1	sti|SiPROV026055m.g	1	sti|SiPROV018471m.g	1	sti|SiPROV013096m.g	1	sti|SiPROV022357m.g	1	sti|SiPROV020431m.g	1
	sti|SiPROV029137m.g	1	sti|SiPROV020097m.g	1	sti|SiPROV011295m.g	1	sti|SiPROV027748m.g	1	sti|SiPROV018671m.g	1	sti|SiPROV026788m.g	1	sti|SiPROV022663m.g	1	sti|SiPROV020459m.g	1
	sti|SiPROV030635m.g	1	sti|SiPROV020335m.g	1	sti|SiPROV011455m.g	1	sti|SiPROV028081m.g	1	sti|SiPROV018910m.g	1	sti|SiPROV033365m.g	1	sti|SiPROV027184m.g	1	sti|SiPROV032121m.g	1
	sti|SiPROV032625m.g	1	sti|SiPROV028656m.g	1	sti|SiPROV011631m.g	1	sti|SiPROV028564m.g	1	sti|SiPROV018952m.g	1	sti|SiPROV035740m.g	1	sti|SiPROV030130m.g	1	sti|SiPROV017922m.g	3
	sti|SiPROV034183m.g	1	sti|SiPROV031150m.g	1	sti|SiPROV013200m.g	1	sti|SiPROV028870m.g	1	sti|SiPROV038092m.g	1	sti|SiPROV011117m.g	2	sti|SiPROV034669m.g	1		
	sti|SiPROV034565m.g	1	sti|SiPROV033054m.g	1	sti|SiPROV013491m.g	1	sti|SiPROV029162m.g	1	sti|SiPROV017273m.g	2	sti|SiPROV011466m.g	2	sti|SiPROV035133m.g	1		
	sti|SiPROV035693m.g	1	sti|SiPROV034714m.g	1	sti|SiPROV016052m.g	1	sti|SiPROV029284m.g	1	sti|SiPROV018205m.g	3	sti|SiPROV027540m.g	2	sti|SiPROV036189m.g	1		
	sti|SiPROV037883m.g	1			sti|SiPROV018221m.g	1	sti|SiPROV031464m.g	1			sti|SiPROV029988m.g	2				
					sti|SiPROV030349m.g	1	sti|SiPROV032676m.g	1			sti|SiPROV030674m.g	2				
					sti|SiPROV030521m.g	1	sti|SiPROV032884m.g	1			sti|SiPROV031834m.g	2				
					sti|SiPROV031331m.g	1	sti|SiPROV033046m.g	1								
					sti|SiPROV032726m.g	1	sti|SiPROV033196m.g	1								
					sti|SiPROV033155m.g	1	sti|SiPROV033484m.g	1								
					sti|SiPROV034174m.g	1	sti|SiPROV034533m.g	1								
					sti|SiPROV035554m.g	1	sti|SiPROV035784m.g	1								
					sti|SiPROV036239m.g	1	sti|SiPROV036232m.g	1								
					sti|SiPROV037003m.g	1	sti|SiPROV037648m.g	1								
							sti|SiPROV018560m.g	2								
							sti|SiPROV019612m.g	2								
							sti|SiPROV026743m.g	2								
							sti|SiPROV029341m.g	2								
							sti|SiPROV029912m.g	2								
							sti|SiPROV030851m.g	2								
							sti|SiPROV033940m.g	2								
							sti|SiPROV034409m.g	2								
							sti|SiPROV035141m.g	2								
							sti|SiPROV030199m.g	3								
zma	zma|AC197705.4_FG004	1	zma|GRMZM2G000353	1	zma|AC212353.4_FG004	1	zma|AC214771.3_FG002	1	zma|GRMZM2G021796	1	zma|GRMZM2G095025	1	zma|GRMZM2G050774	1	zma|AC206768.3_FG005	1
	zma|AC220972.3_FG001	1	zma|GRMZM2G031280	1	zma|AC233979.1_FG011	1	zma|GRMZM2G026562	1	zma|GRMZM2G028234	1	zma|GRMZM2G101559	1	zma|GRMZM2G055973	1	zma|GRMZM2G028188	1
	zma|GRMZM2G004519	1	zma|GRMZM2G057789	1	zma|GRMZM2G003725	1	zma|GRMZM2G033855	1	zma|GRMZM2G052344	1	zma|GRMZM2G117361	1	zma|GRMZM2G068128	1	zma|GRMZM2G029623	1
	zma|GRMZM2G028543	1	zma|GRMZM2G059110	1	zma|GRMZM2G004480	1	zma|GRMZM2G038335	1	zma|GRMZM2G068239	1	zma|GRMZM2G131822	1	zma|GRMZM2G324375	1	zma|GRMZM2G033086	1
	zma|GRMZM2G044773	1	zma|GRMZM2G081285	1	zma|GRMZM2G015409	1	zma|GRMZM2G087350	1	zma|GRMZM2G080079	1	zma|GRMZM2G132751	1	zma|GRMZM2G400109	1	zma|GRMZM2G135011	1
	zma|GRMZM2G049070	1	zma|GRMZM2G083382	1	zma|GRMZM2G016362	1	zma|GRMZM2G098925	1	zma|GRMZM2G108085	1	zma|GRMZM2G329195	1	zma|GRMZM2G420012	1	zma|GRMZM2G145382	1
	zma|GRMZM2G055827	1	zma|GRMZM2G146847	1	zma|GRMZM2G017636	1	zma|GRMZM2G107926	1	zma|GRMZM2G112617	1	zma|GRMZM2G475521	1			zma|GRMZM2G356895	1

	zma|GRMZM2G075782	1			zma|GRMZM2G312078	1	zma|GRMZM2G024312	1	zma|GRMZM2G121143	1	zma|GRMZM2G132531	1	zma|GRMZM2G534826	1			zma|GRMZM2G383937	1
	zma|GRMZM2G302912	1			zma|GRMZM2G321041	1	zma|GRMZM2G037627	1	zma|GRMZM2G127266	1	zma|GRMZM2G140651	1	zma|GRMZM2G015753	2			zma|GRMZM2G482046	1
	zma|GRMZM2G358987	1			zma|GRMZM2G341159	1	zma|GRMZM2G041344	1	zma|GRMZM2G163539	1	zma|GRMZM2G164426	1	zma|GRMZM2G035704	2			zma|GRMZM2G469371	2
	zma|GRMZM2G416750	1			zma|GRMZM2G361902	1	zma|GRMZM2G041549	1	zma|GRMZM2G358711	1	zma|GRMZM2G168200	1	zma|GRMZM2G082653	2			zma|GRMZM2G021879	3
	zma|GRMZM2G460958	1			zma|GRMZM2G390436	1	zma|GRMZM2G042538	1	zma|GRMZM2G384913	1	zma|GRMZM2G428179	1	zma|GRMZM2G105460	2				
	zma|GRMZM2G478553	1			zma|GRMZM2G401009	1	zma|GRMZM2G053303	1	zma|GRMZM2G386584	1	zma|GRMZM2G440866	1	zma|GRMZM2G148706	2				
					zma|GRMZM2G426613	1	zma|GRMZM2G056786	1	zma|GRMZM2G401997	1	zma|GRMZM2G069923	3	zma|GRMZM2G323013	2				
					zma|GRMZM2G440125	1	zma|GRMZM2G056804	1	zma|GRMZM2G433908	1			zma|GRMZM2G400784	2				
					zma|GRMZM2G451308	1	zma|GRMZM2G090279	1	zma|GRMZM2G534288	1			zma|GRMZM2G467187	2				
					zma|GRMZM2G467576	2	zma|GRMZM2G122284	1	zma|GRMZM2G020329	2			zma|GRMZM2G160966	3				
							zma|GRMZM2G124423	1	zma|GRMZM2G040236	2								
							zma|GRMZM2G146228	1	zma|GRMZM2G132632	2								
							zma|GRMZM2G151913	1	zma|GRMZM2G324111	2								
							zma|GRMZM2G171277	1	zma|GRMZM2G480106	2								
							zma|GRMZM2G172065	1	zma|GRMZM2G447566	3								
							zma|GRMZM2G368765	1										
							zma|GRMZM2G375153	1										
							zma|GRMZM2G416216	1										
							zma|GRMZM2G457411	1										
sbi	sbi|Sb01g033150	1			sbi|Sb01g030420	1	sbi|Sb01g028660	1	sbi|Sb03g001970	1	sbi|Sb01g030190	1	sbi|Sb04g023620	1	sbi|Sb04g005300	1	sbi|Sb01g005810	1
	sbi|Sb01g035920	1			sbi|Sb01g048530	1	sbi|Sb01g046930	1	sbi|Sb03g001980	1	sbi|Sb02g031500	1	sbi|Sb05g027490	1	sbi|Sb04g021520	1	sbi|Sb01g030040	1
	sbi|Sb01g035930	1			sbi|Sb02g032320	1	sbi|Sb01g046940	1	sbi|Sb04g004780	1	sbi|Sb02g033150	1	sbi|Sb05g027500	1	sbi|Sb04g029050	1	sbi|Sb02g003340	1
	sbi|Sb02g039360	1			sbi|Sb03g038270	1	sbi|Sb02g023600	1	sbi|Sb04g009000	1	sbi|Sb03g034930	1	sbi|Sb05g027540	1	sbi|Sb04g029060	1	sbi|Sb02g042800	1
	sbi|Sb03g012750	1			sbi|Sb03g040870	1	sbi|Sb02g027220	1	sbi|Sb04g009010	1	sbi|Sb04g022820	1	sbi|Sb05g027550	1	sbi|Sb05g003050	1	sbi|Sb03g033910	1
	sbi|Sb03g038810	1			sbi|Sb04g022150	1	sbi|Sb04g023630	1	sbi|Sb04g009020	1	sbi|Sb07g024770	1	sbi|Sb05g027560	1	sbi|Sb06g031400	1	sbi|Sb08g021970	1
	sbi|Sb04g023040	1			sbi|Sb04g031560	1	sbi|Sb04g028200	1	sbi|Sb04g009330	1	sbi|Sb09g004770	1	sbi|Sb09g026590	1	sbi|Sb10g009860	1	sbi|Sb08g021990	1
	sbi|Sb04g023060	1			sbi|Sb04g038490	1	sbi|Sb04g031240	1	sbi|Sb08g002420	1	sbi|Sb10g008240	1	sbi|Sb09g030900	1			sbi|Sb09g026220	1
	sbi|Sb04g023070	1			sbi|Sb06g016320	1	sbi|Sb04g031430	1	sbi|Sb09g008150	1	sbi|Sb04g028130	2	sbi|Sb10g020965	1			sbi|Sb10g021050	3
	sbi|Sb05g027570	1			sbi|Sb06g026580	1	sbi|Sb04g035570	1	sbi|Sb10g003830	1	sbi|Sb10g030310	3	sbi|Sb10g020970	1				
	sbi|Sb05g027580	1			sbi|Sb08g002625	1	sbi|Sb04g037520	1	sbi|Sb10g021077	1			sbi|Sb10g028540	1				
	sbi|Sb06g017730	1			sbi|Sb09g023450	1	sbi|Sb05g024250	1	sbi|Sb10g021080	1			sbi|Sb01g044650	2				
	sbi|Sb09g017860	1			sbi|Sb10g026650	1	sbi|Sb06g018375	1	sbi|Sb10g021090	1			sbi|Sb02g027200	2				
	sbi|Sb09g017880	1			sbi|Sb10g030990	1	sbi|Sb06g026980	1	sbi|Sb03g001960	2			sbi|Sb04g023600	2				
	sbi|Sb09g017890	1			sbi|Sb08g002630	2	sbi|Sb07g021910	1	sbi|Sb04g008070	2			sbi|Sb04g033820	2				
	sbi|Sb09g023050	1					sbi|Sb08g020130	1	sbi|Sb04g008080	2			sbi|Sb10g007490	2				
	sbi|Sb10g006200	1					sbi|Sb08g020240	1	sbi|Sb04g008890	2								
							sbi|Sb10g004710	1	sbi|Sb04g008920	2								
							sbi|Sb10g005930	1	sbi|Sb04g008940	2								
							sbi|Sb10g008340	1	sbi|Sb04g008950	2								
									sbi|Sb04g008960	2								
									sbi|Sb04g008970	2								
									sbi|Sb04g008980	2								
									sbi|Sb04g008990	2								
									sbi|Sb04g009320	2								
									sbi|Sb10g020980	2								
									sbi|Sb10g020990	2								
									sbi|Sb10g021020	2								
									sbi|Sb10g021070	2								
									sbi|Sb10g027410	2								
aco	aco|AcoGoldSmith_v1.014546m.g	1	aco|AcoGoldSmith_v1.013754m.g	1	aco|AcoGoldSmith_v1.012513m.g	1	aco|AcoGoldSmith_v1.003476m.g	1			aco|AcoGoldSmith_v1.009530m.g	1	aco|AcoGoldSmith_v1.005679m.g	1	aco|AcoGoldSmith_v1.010656m.g	1	aco|AcoGoldSmith_v1.015429m.g	1

	aco|AcoGoldSmith_v1.018184m.g	1	aco|AcoGoldSmith_v1.017218m.g	1	aco|AcoGoldSmith_v1.014768m.g	1	aco|AcoGoldSmith_v1.006514m.g	1	aco|AcoGoldSmith_v1.010307m.g	1	aco|AcoGoldSmith_v1.015275m.g	1	aco|AcoGoldSmith_v1.011128m.g	1	aco|AcoGoldSmith_v1.016895m.g	1
	aco|AcoGoldSmith_v1.018514m.g	1	aco|AcoGoldSmith_v1.018980m.g	1	aco|AcoGoldSmith_v1.015947m.g	1	aco|AcoGoldSmith_v1.007272m.g	1	aco|AcoGoldSmith_v1.010617m.g	1	aco|AcoGoldSmith_v1.021629m.g	1	aco|AcoGoldSmith_v1.012827m.g	1	aco|AcoGoldSmith_v1.020546m.g	1
	aco|AcoGoldSmith_v1.021797m.g	1	aco|AcoGoldSmith_v1.025500m.g	1	aco|AcoGoldSmith_v1.016596m.g	1	aco|AcoGoldSmith_v1.017839m.g	1	aco|AcoGoldSmith_v1.011994m.g	1	aco|AcoGoldSmith_v1.024884m.g	1	aco|AcoGoldSmith_v1.021744m.g	1	aco|AcoGoldSmith_v1.021001m.g	1
	aco|AcoGoldSmith_v1.026949m.g	1			aco|AcoGoldSmith_v1.017388m.g	1	aco|AcoGoldSmith_v1.019174m.g	1	aco|AcoGoldSmith_v1.020422m.g	1	aco|AcoGoldSmith_v1.024943m.g	1	aco|AcoGoldSmith_v1.022017m.g	1	aco|AcoGoldSmith_v1.021519m.g	1
					aco|AcoGoldSmith_v1.022512m.g	1	aco|AcoGoldSmith_v1.020696m.g	1	aco|AcoGoldSmith_v1.021743m.g	1	aco|AcoGoldSmith_v1.026202m.g	1	aco|AcoGoldSmith_v1.022944m.g	1	aco|AcoGoldSmith_v1.025270m.g	1
					aco|AcoGoldSmith_v1.024188m.g	1	aco|AcoGoldSmith_v1.024677m.g	1	aco|AcoGoldSmith_v1.013878m.g	2	aco|AcoGoldSmith_v1.026816m.g	1				
					aco|AcoGoldSmith_v1.026608m.g	1			aco|AcoGoldSmith_v1.018542m.g	2	aco|AcoGoldSmith_v1.005655m.g	2				
					aco|AcoGoldSmith_v1.027069m.g	1			aco|AcoGoldSmith_v1.010482m.g	3	aco|AcoGoldSmith_v1.007770m.g	2				
											aco|AcoGoldSmith_v1.024946m.g	2				
mgu	mgu|mgv1a011050m.g	1	mgu|mgv1a018412m.g	1	mgu|mgv1a014346m.g	1	mgu|mgv1a007689m.g	1	mgu|mgv1a012315m.g	1	mgu|mgv1a011264m.g	1	mgu|mgv1a013505m.g	1	mgu|mgv1a008365m.g	1
	mgu|mgv1a013568m.g	1	mgu|mgv1a019310m.g	1	mgu|mgv1a014454m.g	1	mgu|mgv1a019339m.g	1	mgu|mgv1a012790m.g	1	mgu|mgv1a013522m.g	1	mgu|mgv1a013959m.g	1	mgu|mgv1a014108m.g	1
	mgu|mgv1a017988m.g	1	mgu|mgv1a026706m.g	1	mgu|mgv1a017957m.g	1	mgu|mgv1a019765m.g	1	mgu|mgv1a016311m.g	1	mgu|mgv1a019491m.g	1	mgu|mgv1a014680m.g	1	mgu|mgv1a014407m.g	1
	mgu|mgv1a021772m.g	1			mgu|mgv1a017986m.g	1	mgu|mgv1a023561m.g	1	mgu|mgv1a017791m.g	1	mgu|mgv1a025282m.g	1	mgu|mgv1a018262m.g	1	mgu|mgv1a015778m.g	1
	mgu|mgv1a023632m.g	1			mgu|mgv1a023071m.g	1	mgu|mgv1a023672m.g	1	mgu|mgv1a020405m.g	1	mgu|mgv1a025601m.g	1	mgu|mgv1a025288m.g	1	mgu|mgv1a017977m.g	1
	mgu|mgv1a024357m.g	1			mgu|mgv1a024730m.g	1	mgu|mgv1a024628m.g	1	mgu|mgv1a022417m.g	1	mgu|mgv1a008144m.g	2	mgu|mgv1a025494m.g	1	mgu|mgv1a018404m.g	1
	mgu|mgv1a012786m.g	2					mgu|mgv1a024633m.g	1	mgu|mgv1a022853m.g	1	mgu|mgv1a025117m.g	2	mgu|mgv1a025712m.g	1	mgu|mgv1a020271m.g	1
							mgu|mgv1a024693m.g	1	mgu|mgv1a023344m.g	1			mgu|mgv1a026536m.g	1	mgu|mgv1a020648m.g	1
							mgu|mgv1a025441m.g	1	mgu|mgv1a025461m.g	1			mgu|mgv1a018092m.g	2	mgu|mgv1a020706m.g	1
							mgu|mgv1a025580m.g	1	mgu|mgv1a013420m.g	2					mgu|mgv1a022735m.g	1
							mgu|mgv1a026985m.g	2	mgu|mgv1a018571m.g	2					mgu|mgv1a024109m.g	1
															mgu|mgv1a024718m.g	1
															mgu|mgv1a026696m.g	1
															mgu|mgv1a023039m.g	2
vvi	vvi|GSVIVG01016661001	1	vvi|GSVIVG01016564001	1	vvi|GSVIVG01022306001	1	vvi|GSVIVG01007793001	1	vvi|GSVIVG01005189001	1	vvi|GSVIVG01015336001	1	vvi|GSVIVG01008754001	1	vvi|GSVIVG01019530001	1
	vvi|GSVIVG01024698001	1			vvi|GSVIVG01032684001	1	vvi|GSVIVG01009096001	1	vvi|GSVIVG01007975001	1	vvi|GSVIVG01000015001	2	vvi|GSVIVG01008756001	1	vvi|GSVIVG01026978001	1
	vvi|GSVIVG01028038001	1					vvi|GSVIVG01013145001	1	vvi|GSVIVG01019592001	1	vvi|GSVIVG01009098001	2	vvi|GSVIVG01027524001	1	vvi|GSVIVG01027094001	1
							vvi|GSVIVG01023776001	1	vvi|GSVIVG01037142001	1	vvi|GSVIVG01029257001	3			vvi|GSVIVG01036593001	1
							vvi|GSVIVG01026703001	1	vvi|GSVIVG01037651001	2					vvi|GSVIVG01038717001	1
							vvi|GSVIVG01028306001	1							vvi|GSVIVG01017156001	11
egr	egr|Egrandis_v1_0.020526m.g	1	egr|Egrandis_v1_0.027937m.g	1	egr|Egrandis_v1_0.025743m.g	1	egr|Egrandis_v1_0.010590m.g	1	egr|Egrandis_v1_0.024403m.g	1	egr|Egrandis_v1_0.013794m.g	1	egr|Egrandis_v1_0.026823m.g	1	egr|Egrandis_v1_0.015488m.g	1
	egr|Egrandis_v1_0.022407m.g	1	egr|Egrandis_v1_0.041268m.g	1	egr|Egrandis_v1_0.026934m.g	1	egr|Egrandis_v1_0.012422m.g	1	egr|Egrandis_v1_0.025170m.g	1	egr|Egrandis_v1_0.025645m.g	1	egr|Egrandis_v1_0.027010m.g	1	egr|Egrandis_v1_0.016486m.g	1
	egr|Egrandis_v1_0.023905m.g	1			egr|Egrandis_v1_0.030181m.g	1	egr|Egrandis_v1_0.013966m.g	1	egr|Egrandis_v1_0.025466m.g	1	egr|Egrandis_v1_0.040449m.g	1	egr|Egrandis_v1_0.030472m.g	1	egr|Egrandis_v1_0.016957m.g	1
	egr|Egrandis_v1_0.027040m.g	1			egr|Egrandis_v1_0.040222m.g	1	egr|Egrandis_v1_0.014222m.g	1	egr|Egrandis_v1_0.025950m.g	1	egr|Egrandis_v1_0.040468m.g	1	egr|Egrandis_v1_0.048520m.g	1	egr|Egrandis_v1_0.028675m.g	1
	egr|Egrandis_v1_0.048044m.g	1			egr|Egrandis_v1_0.049079m.g	1	egr|Egrandis_v1_0.015210m.g	1	egr|Egrandis_v1_0.026696m.g	1	egr|Egrandis_v1_0.040614m.g	1	egr|Egrandis_v1_0.052286m.g	1	egr|Egrandis_v1_0.039015m.g	1
	egr|Egrandis_v1_0.049275m.g	1			egr|Egrandis_v1_0.055192m.g	1	egr|Egrandis_v1_0.019899m.g	1	egr|Egrandis_v1_0.029640m.g	1	egr|Egrandis_v1_0.047503m.g	1			egr|Egrandis_v1_0.039538m.g	1
							egr|Egrandis_v1_0.038878m.g	1	egr|Egrandis_v1_0.038523m.g	1	egr|Egrandis_v1_0.051096m.g	1			egr|Egrandis_v1_0.039779m.g	1
							egr|Egrandis_v1_0.039689m.g	1	egr|Egrandis_v1_0.023396m.g	2	egr|Egrandis_v1_0.052939m.g	1			egr|Egrandis_v1_0.040560m.g	1
							egr|Egrandis_v1_0.043129m.g	1	egr|Egrandis_v1_0.039515m.g	2	egr|Egrandis_v1_0.013326m.g	2			egr|Egrandis_v1_0.040992m.g	1
							egr|Egrandis_v1_0.054012m.g	1	egr|Egrandis_v1_0.046126m.g	2	egr|Egrandis_v1_0.014278m.g	2			egr|Egrandis_v1_0.044102m.g	1
							egr|Egrandis_v1_0.054915m.g	1	egr|Egrandis_v1_0.024673m.g	3	egr|Egrandis_v1_0.049009m.g	2			egr|Egrandis_v1_0.044335m.g	1
							egr|Egrandis_v1_0.055024m.g	1			egr|Egrandis_v1_0.049805m.g	2			egr|Egrandis_v1_0.049123m.g	1
							egr|Egrandis_v1_0.055178m.g	1			egr|Egrandis_v1_0.016170m.g	3			egr|Egrandis_v1_0.050415m.g	1
															egr|Egrandis_v1_0.051045m.g	1
															egr|Egrandis_v1_0.051235m.g	1
															egr|Egrandis_v1_0.051396m.g	1
															egr|Egrandis_v1_0.052297m.g	1
															egr|Egrandis_v1_0.054217m.g	1
ccl	ccl|clementine0.9_016810m.g	1	ccl|clementine0.9_030163m.g	1	ccl|clementine0.9_020692m.g	1	ccl|clementine0.9_008411m.g	1	ccl|clementine0.9_019590m.g	1	ccl|clementine0.9_032218m.g	1	ccl|clementine0.9_020892m.g	1	ccl|clementine0.9_011644m.g	1
	ccl|clementine0.9_018811m.g	1	ccl|clementine0.9_031488m.g	1	ccl|clementine0.9_021416m.g	1	ccl|clementine0.9_011238m.g	1	ccl|clementine0.9_020481m.g	1	ccl|clementine0.9_032560m.g	1	ccl|clementine0.9_022761m.g	1	ccl|clementine0.9_013467m.g	1
	ccl|clementine0.9_020554m.g	1			ccl|clementine0.9_021420m.g	1	ccl|clementine0.9_012404m.g	1	ccl|clementine0.9_021622m.g	1	ccl|clementine0.9_010217m.g	2	ccl|clementine0.9_023565m.g	1	ccl|clementine0.9_022451m.g	1

	ccl|clementine0.9_029006m.g	1			ccl|clementine0.9_022456m.g	1	ccl|clementine0.9_012652m.g	1			ccl|clementine0.9_024091m.g	1	ccl|clementine0.9_011752m.g	2	ccl|clementine0.9_023927m.g	1	ccl|clementine0.9_032341m.g	1
	ccl|clementine0.9_031044m.g	1			ccl|clementine0.9_028772m.g	1	ccl|clementine0.9_013899m.g	1			ccl|clementine0.9_035691m.g	1	ccl|clementine0.9_012790m.g	2	ccl|clementine0.9_024847m.g	1	ccl|clementine0.9_033866m.g	1
					ccl|clementine0.9_031204m.g	1	ccl|clementine0.9_013901m.g	1			ccl|clementine0.9_020518m.g	2	ccl|clementine0.9_030155m.g	2	ccl|clementine0.9_028064m.g	1	ccl|clementine0.9_034929m.g	1
					ccl|clementine0.9_031327m.g	1	ccl|clementine0.9_014061m.g	1									ccl|clementine0.9_034967m.g	1
					ccl|clementine0.9_032013m.g	1	ccl|clementine0.9_014348m.g	1									ccl|clementine0.9_034595m.g	2
					ccl|clementine0.9_032376m.g	1	ccl|clementine0.9_017232m.g	1										
					ccl|clementine0.9_033273m.g	1	ccl|clementine0.9_027729m.g	1										
					ccl|clementine0.9_035169m.g	1	ccl|clementine0.9_032826m.g	1										
					ccl|clementine0.9_035931m.g	1	ccl|clementine0.9_033652m.g	1										
					ccl|clementine0.9_019363m.g	2	ccl|clementine0.9_036081m.g	1										
csi	csi|orange1.1g021708m.g	1	csi|orange1.1g040159m.g	1	csi|orange1.1g026797m.g	1	csi|orange1.1g015232m.g	1			csi|orange1.1g025541m.g	1	csi|orange1.1g016846m.g	1	csi|orange1.1g027163m.g	1	csi|orange1.1g029203m.g	1
	csi|orange1.1g024682m.g	1			csi|orange1.1g027813m.g	1	csi|orange1.1g016298m.g	1			csi|orange1.1g025788m.g	1	csi|orange1.1g041871m.g	1	csi|orange1.1g029544m.g	1	csi|orange1.1g035703m.g	1
	csi|orange1.1g026517m.g	1			csi|orange1.1g028342m.g	1	csi|orange1.1g018110m.g	1			csi|orange1.1g026690m.g	1	csi|orange1.1g047079m.g	1	csi|orange1.1g030757m.g	1	csi|orange1.1g037839m.g	1
	csi|orange1.1g036506m.g	1			csi|orange1.1g029206m.g	1	csi|orange1.1g019053m.g	1			csi|orange1.1g028116m.g	1	csi|orange1.1g048010m.g	1	csi|orange1.1g032328m.g	1	csi|orange1.1g043654m.g	1
					csi|orange1.1g035743m.g	1	csi|orange1.1g036833m.g	1			csi|orange1.1g031354m.g	1	csi|orange1.1g014794m.g	2	csi|orange1.1g035583m.g	1	csi|orange1.1g044271m.g	1
					csi|orange1.1g038049m.g	1	csi|orange1.1g037216m.g	1			csi|orange1.1g042206m.g	1	csi|orange1.1g039186m.g	2	csi|orange1.1g037529m.g	1	csi|orange1.1g044489m.g	2
					csi|orange1.1g042698m.g	1	csi|orange1.1g038999m.g	1			csi|orange1.1g045853m.g	1	csi|orange1.1g045417m.g	2	csi|orange1.1g040080m.g	1	csi|orange1.1g044813m.g	2
					csi|orange1.1g042990m.g	1	csi|orange1.1g039247m.g	1			csi|orange1.1g024603m.g	2			csi|orange1.1g042258m.g	1		
					csi|orange1.1g043163m.g	1	csi|orange1.1g039646m.g	1							csi|orange1.1g045184m.g	1		
					csi|orange1.1g043554m.g	1	csi|orange1.1g042083m.g	1							csi|orange1.1g045986m.g	1		
					csi|orange1.1g046833m.g	1	csi|orange1.1g042750m.g	1										
							csi|orange1.1g044619m.g	1										
cpa	cpa|evm.TU.supercontig_151.16	1	cpa|evm.TU.supercontig_260.1	1	cpa|evm.TU.supercontig_0.117	1	cpa|evm.TU.supercontig_124.29	1			cpa|evm.TU.supercontig_111.28	1	cpa|evm.TU.contig_32183.1	1	cpa|evm.TU.supercontig_18.195	1	cpa|evm.TU.supercontig_11.63	1
	cpa|evm.TU.supercontig_2.425	1	cpa|evm.TU.supercontig_28.83	1	cpa|evm.TU.supercontig_19.61	1	cpa|evm.TU.supercontig_140.37	1			cpa|evm.TU.supercontig_15.6	1	cpa|evm.TU.supercontig_55.59	1	cpa|evm.TU.supercontig_26.274	1	cpa|evm.TU.supercontig_12.98	1
	cpa|evm.TU.supercontig_209.12	1			cpa|evm.TU.supercontig_26.150	1	cpa|evm.TU.supercontig_3591.1	1			cpa|evm.TU.supercontig_2.424	1	cpa|evm.TU.supercontig_90.54	1	cpa|evm.TU.supercontig_80.56	1	cpa|evm.TU.supercontig_1467.3	1
	cpa|evm.TU.supercontig_25.200	1			cpa|evm.TU.supercontig_42.96	1	cpa|evm.TU.supercontig_42.20	1			cpa|evm.TU.supercontig_26.270	1	cpa|evm.TU.supercontig_1183.1	2			cpa|evm.TU.supercontig_3.66	1
	cpa|evm.TU.supercontig_41.56	1			cpa|evm.TU.supercontig_42.97	1	cpa|evm.TU.supercontig_44.71	1			cpa|evm.TU.supercontig_6.310	1	cpa|evm.TU.supercontig_19.226	2			cpa|evm.TU.supercontig_96.8	1
	cpa|evm.TU.supercontig_50.166	1					cpa|evm.TU.supercontig_50.30	1			cpa|evm.TU.supercontig_81.149	1						
							cpa|evm.TU.supercontig_52.73	1			cpa|evm.TU.supercontig_81.150	1						
							cpa|evm.TU.supercontig_55.65	1			cpa|evm.TU.supercontig_81.151	1						
							cpa|evm.TU.supercontig_69.2	1			cpa|evm.TU.supercontig_81.152	1						
											cpa|evm.TU.supercontig_12.307	2						
											cpa|evm.TU.supercontig_80.57	2						
aly	aly|329916	1	aly|345494	1	aly|316562	1	aly|328381	1			aly|336421	1	aly|473425	1	aly|474421	1	aly|313509	1
	aly|476359	1			aly|472318	1	aly|350245	1			aly|489863	1	aly|482507	1	aly|481807	1	aly|482561	1
	aly|478848	1			aly|474062	1	aly|472661	1			aly|492461	1	aly|489785	1	aly|491715	1	aly|483761	1
	aly|483328	1			aly|474063	1	aly|477600	1			aly|923695	1	aly|491868	1	aly|494813	1	aly|939208	1
	aly|483329	1			aly|474064	1	aly|479408	1			aly|928824	1	aly|900541	1	aly|931769	1	aly|940090	1
	aly|483937	1			aly|478427	1	aly|480768	1			aly|931636	1	aly|940009	1			aly|940152	1
	aly|486484	1			aly|479387	1	aly|486491	1			aly|941700	1	aly|942690	1			aly|949894	1
	aly|495981	1			aly|480629	1	aly|488627	1			aly|947587	1	aly|942695	1			aly|349229	2
	aly|923943	1			aly|487321	1	aly|493966	1			aly|339633	2	aly|946420	1			aly|483804	2
	aly|935395	1			aly|491099	1	aly|494656	1			aly|947574	2	aly|472490	2				
	aly|935396	1			aly|494214	1	aly|919111	1			aly|491055	3	aly|476353	2				
	aly|939357	1			aly|929765	1	aly|926585	1			aly|497009	3	aly|477807	2				
	aly|479140	2			aly|939472	1	aly|944951	1			aly|905044	3	aly|480466	2				
	aly|944017	2					aly|937551	3					aly|913385	2				
							aly|354960	13					aly|942355	2				
													aly|942692	2				
													aly|942696	2				

													aly|934108	3				
ath	ATL17|At4g15975	1	ATL33|At2g37580	1	ATL10|At1g49220	1	ATL1|At1g04360	1			ATL25|At2g17730	1	ATL28|At2g35420	1	ATL14|At4g30370	1	ATL20|At1g28040	1
	ATL3|At1g72310	1			ATL44|At2g17450	1	ATL13|At4g30400	1			ATL58|At1g33480	1	ATL29|At4g17920	1	ATL19|At1g53010	1	ATL67|At2g46160	1
	ATL40|At2g42350	1			ATL45|At4g35480	1	ATL16|At5g43420	1			ATL59|At4g10160	1	ATL35|At4g09110	1	ATL23|At5g42200	1	ATL68|At3g61550	1
	ATL41|At2g42360	1			ATL72|At3g10910	1	ATL4|At3g60220	1			ATL66|At3g11110	1	ATL38|At2g34990	1	ATL56|At2g18670	1	ATL69|At5g07040	1
	ATL5|At3g62690	1			ATL73|At5g05280	1	ATL46|At5g40250	1			ATL7|At4g10150	1	ATL39|At4g09100	1	ATL84|AT2G28920	1	ATL70|At2g35910	1
	ATL60|At1g53820	1			ATL74|At5g01880	1	ATL47|At1g23980	1			ATL82|At4g24015	1	ATL42|At4g28890	1			ATL71|At5g06490	1
	ATL61|At3g14320	1			ATL75|At1g49200	1	ATL49|At2g18650	1			ATL85|AT5G41430	1	ATL43|At5g05810	1			ATL21|At2g46495	2
	ATL62|At3g19140	1			ATL76|At1g49210	1	ATL50|At5g57750	1			ATL86|AT5G41440	1	ATL57|At2g27940	1			ATL22|At2g25410	2
	ATL63|At5g58580	1			ATL77|At3g18773	1	ATL51|At3g03550	1			ATL87|AT5G41450	1	ATL93|AT1G32361	1				
	ATL64|At2g47560	1			ATL78|At1g49230	1	ATL52|At5g17600	1			ATL88|AT1G51930	1	ATL11|At1g72200	2				
	ATL89|AT2G44578	1			ATL79|At5g47610	1	ATL53|At4g17905	1			ATL24|At1g74410	2	ATL12|At2g20030	2				
	ATL90|AT2G44581	1			ATL8|At1g76410	1	ATL54|At1g72220	1			ATL26|At4g35840	3	ATL15|At1g22500	2				
	ATL91|AT3G60966	1			ATL80|At1g20823	1	ATL55|At5g10380	1			ATL27|At5g66070	3	ATL31|At5g27420	2				
	ATL2|At3g16720	2			ATL81|At3g18777	1	ATL65|At3g18930	1			ATL92|AT3G20395	3	ATL34|At1g35330	2				
	ATL32|At4g40070	2					ATL83|AT4G33565	2					ATL36|At4g09120	2				
							ATL48|At3g48030	3					ATL37|At4g09130	2				
													ATL6|At3g05200	2				
													ATL9|At2g35000	2				
csa	csa|Cucsa.046720	1	csa|Cucsa.010620	1	csa|Cucsa.048690	1	csa|Cucsa.053490	1			csa|Cucsa.117930	1	csa|Cucsa.311740	1	csa|Cucsa.074760	1	csa|Cucsa.005140	1
	csa|Cucsa.069210	1	csa|Cucsa.141560	1	csa|Cucsa.101150	1	csa|Cucsa.063720	1			csa|Cucsa.237760	1	csa|Cucsa.313000	1	csa|Cucsa.089780	1	csa|Cucsa.040640	1
	csa|Cucsa.139340	1	csa|Cucsa.175370	1	csa|Cucsa.130520	1	csa|Cucsa.089790	1			csa|Cucsa.304000	1	csa|Cucsa.385620	1	csa|Cucsa.098350	1	csa|Cucsa.083830	1
	csa|Cucsa.149170	1			csa|Cucsa.193250	1	csa|Cucsa.098300	1			csa|Cucsa.322860	1	csa|Cucsa.091660	2	csa|Cucsa.123190	1	csa|Cucsa.107410	1
	csa|Cucsa.151710	1			csa|Cucsa.250040	1	csa|Cucsa.104190	1			csa|Cucsa.368380	1	csa|Cucsa.104160	2	csa|Cucsa.364780	1	csa|Cucsa.242010	1
	csa|Cucsa.185000	1			csa|Cucsa.272320	1	csa|Cucsa.109930	1			csa|Cucsa.380880	1	csa|Cucsa.160260	2			csa|Cucsa.258750	1
	csa|Cucsa.252890	1			csa|Cucsa.323250	1	csa|Cucsa.158970	1			csa|Cucsa.007210	2	csa|Cucsa.261420	2				
	csa|Cucsa.359760	1			csa|Cucsa.326000	1	csa|Cucsa.160240	1					csa|Cucsa.261430	2				
					csa|Cucsa.379840	1	csa|Cucsa.218410	1					csa|Cucsa.281910	2				
							csa|Cucsa.255310	1										
							csa|Cucsa.256790	1										
							csa|Cucsa.256910	1										
							csa|Cucsa.265900	1										
							csa|Cucsa.285900	1										
							csa|Cucsa.313020	1										
							csa|Cucsa.383690	1										
mtr	mtr|Medtr2g132500	1	mtr|Medtr4g023410	1	mtr|Medtr2g114410	1	mtr|Medtr1g042550	1			mtr|Medtr3g094490	1	mtr|Medtr3g102290	1	mtr|Medtr1g032030	1	mtr|AC235758_12	1
	mtr|Medtr5g080230	1	mtr|Medtr4g023620	1	mtr|Medtr3g144110	1	mtr|Medtr2g009360	1			mtr|Medtr4g031670	1	mtr|Medtr3g102310	1	mtr|Medtr3g157100	1	mtr|Medtr1g112170	1
	mtr|Medtr8g026830	1	mtr|Medtr5g026870	1	mtr|Medtr3g159960	1	mtr|Medtr2g052120	1			mtr|Medtr5g090410	1	mtr|Medtr5g084870	1	mtr|Medtr7g083870	1	mtr|Medtr1g112200	1
			mtr|Medtr5g026890	1	mtr|Medtr4g079030	1	mtr|Medtr3g123770	1			mtr|Medtr5g100100	1	mtr|Medtr8g078070	1	mtr|Medtr8g127780	1	mtr|Medtr1g149630	1
			mtr|Medtr8g086300	1	mtr|Medtr4g089780	1	mtr|Medtr4g120960	1			mtr|Medtr5g022920	3	mtr|Medtr1g099190	2			mtr|Medtr3g124280	1
					mtr|Medtr4g141950	1	mtr|Medtr5g029880	1			mtr|Medtr8g089110	3	mtr|Medtr3g149320	2			mtr|Medtr5g009230	1
					mtr|Medtr4g142090	1	mtr|Medtr5g035920	1					mtr|Medtr3g150140	2			mtr|Medtr5g009250	1
					mtr|Medtr4g142220	1	mtr|Medtr5g074060	1					mtr|Medtr5g074150	2			mtr|Medtr5g009270	1
					mtr|Medtr5g024680	1	mtr|Medtr8g008990	1					mtr|Medtr5g074170	2			mtr|Medtr7g089030	1
					mtr|Medtr7g080330	1	mtr|Medtr8g078100	1					mtr|Medtr5g075250	2			mtr|Medtr7g089020	2
					mtr|Medtr8g087590	1	mtr|Medtr8g125350	1					mtr|Medtr6g059190	2				
							mtr|Medtr8g127740	1					mtr|Medtr6g098760	2				
													mtr|Medtr7g139570	2				
gma	gma|Glyma02g02040	1	gma|Glyma09g34780	1	gma|Glyma01g36160	1	gma|Glyma01g02140	1			gma|Glyma02g43250	1	gma|Glyma01g02130	1	gma|Glyma04g02340	1	gma|Glyma02g35090	1
	gma|Glyma02g39400	1	gma|Glyma13g16830	1	gma|Glyma04g01680	1	gma|Glyma01g03900	1			gma|Glyma02g46060	1	gma|Glyma01g10600	1	gma|Glyma06g02390	1	gma|Glyma03g36170	1

	gma|Glyma03g37360	1	gma|Glyma15g19030	1	gma|Glyma04g39360	1	gma|Glyma01g11110	1	gma|Glyma05g00900	1	gma|Glyma01g34830	1	gma|Glyma09g40020	1	gma|Glyma04g14380	1
	gma|Glyma03g42390	1	gma|Glyma17g05870	1	gma|Glyma04g40020	1	gma|Glyma02g03780	1	gma|Glyma05g36680	1	gma|Glyma06g08930	1	gma|Glyma10g34640	1	gma|Glyma05g36870	1
	gma|Glyma06g43730	1	gma|Glyma18g38530	2	gma|Glyma05g26410	1	gma|Glyma02g37290	1	gma|Glyma08g02860	1	gma|Glyma06g10460	1	gma|Glyma12g35230	1	gma|Glyma06g13270	1
	gma|Glyma07g05190	1			gma|Glyma05g32240	1	gma|Glyma04g15820	1	gma|Glyma08g42840	1	gma|Glyma08g36560	1	gma|Glyma13g01460	1	gma|Glyma07g06200	1
	gma|Glyma07g37470	1			gma|Glyma06g01770	1	gma|Glyma05g30920	1	gma|Glyma09g00380	1	gma|Glyma09g26080	1	gma|Glyma13g23930	1	gma|Glyma08g02670	1
	gma|Glyma09g04750	1			gma|Glyma06g14830	1	gma|Glyma06g46730	1	gma|Glyma11g27890	1	gma|Glyma09g26100	1	gma|Glyma14g40110	1	gma|Glyma09g38870	1
	gma|Glyma11g27400	1			gma|Glyma06g15550	1	gma|Glyma07g12990	1	gma|Glyma11g35490	1	gma|Glyma11g37850	1	gma|Glyma17g07580	1	gma|Glyma09g38880	1
	gma|Glyma11g27880	1			gma|Glyma08g09320	1	gma|Glyma08g07470	1	gma|Glyma13g10570	1	gma|Glyma12g08780	1	gma|Glyma17g38020	1	gma|Glyma10g10280	1
	gma|Glyma12g14190	1			gma|Glyma08g15490	1	gma|Glyma08g18870	1	gma|Glyma14g06300	1	gma|Glyma14g22800	1	gma|Glyma19g01340	1	gma|Glyma16g03430	1
	gma|Glyma12g33620	1			gma|Glyma09g32910	1	gma|Glyma08g36600	1	gma|Glyma17g11000	1	gma|Glyma14g35620	1	gma|Glyma20g32920	1	gma|Glyma19g44470	1
	gma|Glyma13g30600	1			gma|Glyma09g41180	1	gma|Glyma08g39940	1	gma|Glyma18g02920	1	gma|Glyma16g31930	1	gma|Glyma04g35240	4	gma|Glyma07g06850	2
	gma|Glyma13g36850	1			gma|Glyma11g09280	1	gma|Glyma09g33800	1	gma|Glyma20g16140	1	gma|Glyma18g01760	1			gma|Glyma16g02830	2
	gma|Glyma14g37530	1			gma|Glyma13g40790	1	gma|Glyma10g01000	1	gma|Glyma07g04130	2	gma|Glyma02g37330	2				
	gma|Glyma15g08640	1			gma|Glyma15g16940	1	gma|Glyma10g04140	1	gma|Glyma01g36760	3	gma|Glyma02g37340	2				
	gma|Glyma16g01700	1			gma|Glyma16g21550	1	gma|Glyma11g13040	1	gma|Glyma02g05000	3	gma|Glyma03g39970	2				
	gma|Glyma17g03160	1			gma|Glyma18g44640	1	gma|Glyma11g37890	1	gma|Glyma11g08540	3	gma|Glyma04g09690	2				
	gma|Glyma18g06760	1					gma|Glyma13g01470	1			gma|Glyma04g10610	2				
	gma|Glyma19g39960	1					gma|Glyma13g04330	1			gma|Glyma09g32670	2				
							gma|Glyma13g08070	1			gma|Glyma10g29750	2				
							gma|Glyma13g18320	1			gma|Glyma14g35580	2				
							gma|Glyma14g35550	1			gma|Glyma19g42510	2				
							gma|Glyma15g06150	1			gma|Glyma20g37560	2				
							gma|Glyma15g20390	1								
							gma|Glyma17g07590	1								
							gma|Glyma17g09930	1								
							gma|Glyma18g01790	1								
							gma|Glyma18g01800	1								
							gma|Glyma18g18480	1								
							gma|Glyma19g01420	1								
							gma|Glyma19g34640	1								
							gma|Glyma20g22040	1								
							gma|Glyma20g34540	1								
ppe	ppe|ppa009161m.g	1	ppe|ppa012149m.g	1	ppe|ppa011056m.g	1	ppe|ppa004734m.g	1	ppe|ppa010296m.g	1	ppe|ppa008615m.g	1	ppe|ppa011047m.g	1	ppe|ppa007460m.g	1
	ppe|ppa010059m.g	1	ppe|ppa018444m.g	1	ppe|ppa011201m.g	1	ppe|ppa005891m.g	1	ppe|ppa010460m.g	1	ppe|ppa009208m.g	1	ppe|ppa011624m.g	1	ppe|ppa012474m.g	1
	ppe|ppa017073m.g	1	ppe|ppa019243m.g	1	ppe|ppa011240m.g	1	ppe|ppa007384m.g	1	ppe|ppa010806m.g	1	ppe|ppa006365m.g	2	ppe|ppa011815m.g	1	ppe|ppa015141m.g	1
	ppe|ppa024544m.g	1	ppe|ppa024559m.g	1	ppe|ppa011446m.g	1	ppe|ppa007393m.g	1	ppe|ppa012636m.g	1	ppe|ppa017787m.g	2	ppe|ppa013156m.g	1	ppe|ppa018265m.g	1
	ppe|ppa026992m.g	1	ppe|ppa027038m.g	1	ppe|ppa012179m.g	1	ppe|ppa007682m.g	1	ppe|ppa016359m.g	1	ppe|ppa019638m.g	2	ppe|ppa015162m.g	1	ppe|ppa024005m.g	1
					ppe|ppa012461m.g	1	ppe|ppa008738m.g	1	ppe|ppa010852m.g	2	ppe|ppa025826m.g	2	ppe|ppa016567m.g	1	ppe|ppa024659m.g	1
					ppe|ppa013731m.g	1	ppe|ppa015676m.g	1			ppe|ppa026744m.g	2	ppe|ppa020248m.g	1	ppe|ppa024917m.g	1
					ppe|ppa017552m.g	1	ppe|ppa018976m.g	1					ppe|ppa015864m.g	2	ppe|ppa025741m.g	1
					ppe|ppa026068m.g	1	ppe|ppa019060m.g	1							ppe|ppa007165m.g	2
							ppe|ppa020409m.g	1							ppe|ppa019102m.g	2
							ppe|ppa021294m.g	1							ppe|ppa024723m.g	2
							ppe|ppa025190m.g	1							ppe|ppa026957m.g	2
							ppe|ppa026237m.g	1							ppe|ppb024146m.g	2
mdo	mdo|MDP0000149492	1	mdo|MDP0000152377	1	mdo|MDP0000137556	1	mdo|MDP0000158126	1	mdo|MDP0000149086	1	mdo|MDP0000317066	1	mdo|MDP0000127494	1	mdo|MDP0000120367	1
	mdo|MDP0000246831	1	mdo|MDP0000172399	1	mdo|MDP0000166108	1	mdo|MDP0000160259	1	mdo|MDP0000150589	1	mdo|MDP0000178196	2	mdo|MDP0000173682	1	mdo|MDP0000149510	1
			mdo|MDP0000199242	1	mdo|MDP0000210058	1	mdo|MDP0000217224	1	mdo|MDP0000170512	1	mdo|MDP0000788345	2	mdo|MDP0000847266	1	mdo|MDP0000247627	1
			mdo|MDP0000248894	1	mdo|MDP0000247371	1	mdo|MDP0000316613	1	mdo|MDP0000259063	1	mdo|MDP0000804895	2			mdo|MDP0000262630	1
			mdo|MDP0000276738	1	mdo|MDP0000591499	1	mdo|MDP0000318455	1	mdo|MDP0000572319	1	mdo|MDP0000909888	2			mdo|MDP0000560623	1

			mdo|MDP0000640726	1	mdo|MDP0000803911	1	mdo|MDP0000467244	1			mdo|MDP0000249255	2					mdo|MDP0000887330	1
			mdo|MDP0000701552	1	mdo|MDP0000853517	1	mdo|MDP0000496515	1									mdo|MDP0000918141	1
			mdo|MDP0000882091	1	mdo|MDP0000220790	2	mdo|MDP0000537022	1									mdo|MDP0000322183	2
			mdo|MDP0000308051	2			mdo|MDP0000679688	1										
							mdo|MDP0000889159	1										
							mdo|MDP0000927098	1										
pop	pop|POPTR_0001s16210	1	pop|POPTR_0003s18290	1	pop|POPTR_0001s15930	1	pop|POPTR_0001s02360	1			pop|POPTR_0001s10920	1	pop|POPTR_0001s02340	1	pop|POPTR_0001s41120	1	pop|POPTR_0001s10610	1
	pop|POPTR_0001s28610	1	pop|POPTR_0006s14650	1	pop|POPTR_0001s24180	1	pop|POPTR_0001s34610	1			pop|POPTR_0002s01860	1	pop|POPTR_0001s21960	1	pop|POPTR_0002s01890	1	pop|POPTR_0002s16670	1
	pop|POPTR_0002s23000	1	pop|POPTR_0008s05430	1	pop|POPTR_0001s31710	1	pop|POPTR_0002s04080	1			pop|POPTR_0003s14260	1	pop|POPTR_0005s17460	1	pop|POPTR_0002s15470	1	pop|POPTR_0002s17150	1
	pop|POPTR_0003s07090	1			pop|POPTR_0001s31720	1	pop|POPTR_0002s10220	1			pop|POPTR_0005s11000	1	pop|POPTR_0018s09200	1	pop|POPTR_0005s26590	1	pop|POPTR_0003s13970	1
	pop|POPTR_0009s07800	1			pop|POPTR_0001s45800	1	pop|POPTR_0002s14150	1			pop|POPTR_0013s09080	1	pop|POPTR_0005s03900	2	pop|POPTR_0006s19030	1	pop|POPTR_0006s21610	1
	pop|POPTR_0010s01400	1			pop|POPTR_0002s00780	1	pop|POPTR_0003s09190	1			pop|POPTR_0128s00200	1	pop|POPTR_0006s25600	2	pop|POPTR_0008s02560	1	pop|POPTR_0008s01970	1
	pop|POPTR_0014s04270	1			pop|POPTR_0003s07270	1	pop|POPTR_0005s07220	1			pop|POPTR_0005s26620	2	pop|POPTR_0013s02670	2	pop|POPTR_0010s24160	1	pop|POPTR_0008s01980	1
	pop|POPTR_0019s12120	1			pop|POPTR_0005s10190	1	pop|POPTR_0006s19080	1			pop|POPTR_0007s09180	3	pop|POPTR_0015s06750	2	pop|POPTR_0011s12060	1	pop|POPTR_0010s24950	1
	pop|POPTR_0267s00200	1			pop|POPTR_0005s27660	1	pop|POPTR_0007s03610	1							pop|POPTR_0014s07290	1	pop|POPTR_0010s24960	1
					pop|POPTR_0007s08550	1	pop|POPTR_0007s04960	1							pop|POPTR_0018s10710	1	pop|POPTR_0010s24970	1
					pop|POPTR_0011s15100	1	pop|POPTR_0008s16580	1							pop|POPTR_0018s10720	1	pop|POPTR_0014s08670	1
					pop|POPTR_0013s09370	1	pop|POPTR_0009s11310	1									pop|POPTR_0014s09380	1
					pop|POPTR_0013s15290	1	pop|POPTR_0010s08340	1									pop|POPTR_0016s06820	1
					pop|POPTR_0019s02610	1	pop|POPTR_0012s07960	1										
					pop|POPTR_0019s02620	1	pop|POPTR_0013s07100	1										
					pop|POPTR_0019s15050	1	pop|POPTR_0014s05250	1										
					pop|POPTR_0019s08600	2	pop|POPTR_0015s08490	1										
							pop|POPTR_0017s07650	1										
							pop|POPTR_0017s10860	1										
							pop|POPTR_0018s10760	1										
							pop|POPTR_0019s06070	1										
rcu	rcu|28806.t000004	1	rcu|29706.t000029	1	rcu|27704.t000004	1	rcu|28717.t000003	1			rcu|29784.t000004	1	rcu|29589.t000057	1	rcu|28597.t000006	1	rcu|29333.t000028	1
	rcu|29646.t000050	1	rcu|29887.t000001	1	rcu|28592.t000003	1	rcu|28829.t000002	1			rcu|29814.t000025	1	rcu|29794.t000115	2	rcu|29616.t000005	1	rcu|29686.t000007	1
	rcu|29726.t000191	1	rcu|29912.t000007	1	rcu|28713.t000003	1	rcu|29584.t000006	1			rcu|29977.t000004	1	rcu|29810.t000003	2	rcu|29822.t000029	1	rcu|29742.t000063	1
	rcu|29900.t000036	1	rcu|30025.t000011	1	rcu|29728.t000033	1	rcu|29603.t000003	1			rcu|30068.t000010	1	rcu|29908.t000150	2	rcu|30147.t000242	1	rcu|29852.t000013	1
	rcu|29931.t000005	1	rcu|30025.t000012	1	rcu|29728.t000037	1	rcu|29709.t000036	1			rcu|30074.t000008	1	rcu|30128.t000199	2	rcu|30170.t000692	1	rcu|30169.t000280	1
	rcu|29986.t000057	1			rcu|29729.t000097	1	rcu|29822.t000043	1			rcu|30138.t000222	1	rcu|30147.t000187	2	rcu|30174.t000357	1	rcu|30190.t000071	1
					rcu|29788.t000013	1	rcu|29838.t000009	1										
					rcu|29915.t000002	1	rcu|29851.t000113	1										
					rcu|30068.t000121	1	rcu|29883.t000096	1										
					rcu|30078.t000020	1	rcu|29908.t000148	1										
					rcu|30170.t000646	1	rcu|30054.t000035	1										
					rcu|30206.t000006	1	rcu|30147.t000254	1										
							rcu|30148.t000045	1										
mes	mes|cassava4.1_012824m.g	1	mes|cassava4.1_021076m.g	1	mes|cassava4.1_015650m.g	1	mes|cassava4.1_006461m.g	1			mes|cassava4.1_014949m.g	1	mes|cassava4.1_017576m.g	1	mes|cassava4.1_014139m.g	1	mes|cassava4.1_016288m.g	1
	mes|cassava4.1_013145m.g	1	mes|cassava4.1_030846m.g	1	mes|cassava4.1_016562m.g	1	mes|cassava4.1_006942m.g	1			mes|cassava4.1_014986m.g	1	mes|cassava4.1_024103m.g	1	mes|cassava4.1_015485m.g	1	mes|cassava4.1_016298m.g	1
	mes|cassava4.1_013501m.g	1	mes|cassava4.1_030938m.g	1	mes|cassava4.1_016688m.g	1	mes|cassava4.1_008477m.g	1			mes|cassava4.1_015188m.g	1	mes|cassava4.1_029953m.g	1	mes|cassava4.1_016737m.g	1	mes|cassava4.1_017766m.g	1
	mes|cassava4.1_030062m.g	1	mes|cassava4.1_031111m.g	1	mes|cassava4.1_016844m.g	1	mes|cassava4.1_009424m.g	1			mes|cassava4.1_015225m.g	1	mes|cassava4.1_034177m.g	1	mes|cassava4.1_017545m.g	1	mes|cassava4.1_020975m.g	1
	mes|cassava4.1_031150m.g	1	mes|cassava4.1_031119m.g	1	mes|cassava4.1_016910m.g	1	mes|cassava4.1_009886m.g	1			mes|cassava4.1_015654m.g	1	mes|cassava4.1_008325m.g	2	mes|cassava4.1_017945m.g	1	mes|cassava4.1_022487m.g	1
	mes|cassava4.1_032102m.g	1	mes|cassava4.1_033991m.g	1	mes|cassava4.1_017114m.g	1	mes|cassava4.1_010672m.g	1			mes|cassava4.1_021144m.g	1	mes|cassava4.1_009826m.g	2	mes|cassava4.1_024474m.g	1	mes|cassava4.1_024421m.g	1
	mes|cassava4.1_033372m.g	1	mes|cassava4.1_034332m.g	1	mes|cassava4.1_017269m.g	1	mes|cassava4.1_010706m.g	1			mes|cassava4.1_023299m.g	1	mes|cassava4.1_021192m.g	2	mes|cassava4.1_026046m.g	1	mes|cassava4.1_028023m.g	1
	mes|cassava4.1_034140m.g	1			mes|cassava4.1_020901m.g	1	mes|cassava4.1_010747m.g	1			mes|cassava4.1_026899m.g	1	mes|cassava4.1_022210m.g	2	mes|cassava4.1_026205m.g	1	mes|cassava4.1_028218m.g	1
					mes|cassava4.1_022624m.g	1	mes|cassava4.1_011591m.g	1			mes|cassava4.1_027015m.g	1	mes|cassava4.1_032946m.g	2	mes|cassava4.1_027072m.g	1	mes|cassava4.1_028394m.g	1
					mes|cassava4.1_026367m.g	1	mes|cassava4.1_023026m.g	1			mes|cassava4.1_022078m.g	3			mes|cassava4.1_027091m.g	1		

				mes|cassava4.1_026690m.g	1	mes|cassava4.1_024216m.g	1							mes|cassava4.1_027501m.g	1		
				mes|cassava4.1_027598m.g	1	mes|cassava4.1_026396m.g	1							mes|cassava4.1_028270m.g	1		
				mes|cassava4.1_028427m.g	1	mes|cassava4.1_027104m.g	1							mes|cassava4.1_032829m.g	1		
				mes|cassava4.1_028577m.g	1	mes|cassava4.1_028511m.g	1							mes|cassava4.1_033087m.g	1		
				mes|cassava4.1_028743m.g	1	mes|cassava4.1_029304m.g	1							mes|cassava4.1_034393m.g	1		
						mes|cassava4.1_030514m.g	1										
						mes|cassava4.1_030522m.g	1										
						mes|cassava4.1_031050m.g	1										
						mes|cassava4.1_032670m.g	1										
						mes|cassava4.1_034133m.g	1										
						mes|cassava4.1_034213m.g	1										
