# Supplementary material for: Diversity in the Architecture of ATLs, a Family of Plant Ubiquitin-Ligases, Leads to Recognition and Targeting of Substrates in Different Cellular Environments
Source: PLoS One. 2011 Aug 24;6(8):e23934. doi: 10.1371/journal.pone.0023934 (PMC3161093; doi:10.1371/journal.pone.0023934)
Supplement: Table S4 — Tandemly arrayed ATL genes. Genes retrieved from 23 plant species. General information and motif architecture is displayed. (PDF) [file pone.0023934.s006.pdf]

# Table S4. Tandem Arrayed Genes and Expansion of *ATL* genes.

<table>
<tr><th>Specie</th><th>ATL Locus</th><th>Scaffold or Chromosome</th><th>Chain</th><th>Star ORF</th><th>End ORF</th><th>Group</th><th>Size</th><th>Motifs architecture</th></tr>
<tr><td>Physcomitrella patens</td><td>ppp|Pp1s185_137V6</td><td>scaffold_185</td><td>1</td><td>881831</td><td>879975</td><td>G</td><td>420</td><td>84_[GLD]_9_[RING-H2]_268</td></tr>
<tr><td></td><td>ppp|Pp1s185_139V6</td><td>scaffold_185</td><td>1</td><td>888221</td><td>885598</td><td>G</td><td>630</td><td>107_[22]_27_[GLD]_[63]_1_[RING-H2]_138_[48]_[17]_154_[67]_28_[28]_19</td></tr>
<tr><td></td><td>ppp|Pp1s20_276V6</td><td>scaffold_20</td><td>-1</td><td>1843959</td><td>1839833</td><td>F</td><td>566</td><td>55_[7]_[52]_31_[GLD]_10_[RING-H2]_383</td></tr>
<tr><td></td><td>ppp|Pp1s20_280V6</td><td>scaffold_20</td><td>-1</td><td>1850727</td><td>1849243</td><td>G</td><td>406</td><td>32_[7]_[43]_12_[GLD]_9_[RING-H2]_262</td></tr>
<tr><td>Selaginella moellendorffii</td><td>smo|8813</td><td>scaffold_64</td><td>1</td><td>177074</td><td>176841</td><td>A</td><td>310</td><td>103_[GLD]_13_[RING-H2]_135</td></tr>
<tr><td></td><td>smo|121181</td><td>scaffold_64</td><td>1</td><td>179492</td><td>178872</td><td>D</td><td>445</td><td>97_[46]_29_[GLD]_11_[RING-H2]_114_[39]_116</td></tr>
<tr><td></td><td>smo|423895</td><td>scaffold_64</td><td>1</td><td>182449</td><td>181523</td><td>G</td><td>308</td><td>105_[GLD]_13_[RING-H2]_97_[40]_18</td></tr>
<tr><td></td><td>smo|28710</td><td>scaffold_65</td><td>1</td><td>579041</td><td>578784</td><td>A</td><td>364</td><td>25_[7]_[43]_11_[46]_11_[GLD]_27_[RING-H2]_192</td></tr>
<tr><td></td><td>smo|29613</td><td>scaffold_65</td><td>-1</td><td>577197</td><td>576973</td><td>A</td><td>352</td><td>27_[7]_[43]_10_[60]_4_[GLD]_1_[20]_[RING-H2]_200</td></tr>
<tr><td>Brachypodium distachyon</td><td>bdi|Bradi1g39000</td><td>Bd1</td><td>1</td><td>35436561</td><td>35434210</td><td>G</td><td>236</td><td>61_[GLD]_22_[RING-H2]_94</td></tr>
<tr><td></td><td>bdi|Bradi1g39010</td><td>Bd1</td><td>1</td><td>35447411</td><td>35446652</td><td>E</td><td>216</td><td>52_[7]_43_[GLD]_13_[RING-H2]_33</td></tr>
<tr><td></td><td>bdi|Bradi1g39020</td><td>Bd1</td><td>-1</td><td>35448948</td><td>35448150</td><td>E</td><td>200</td><td>76_[GLD]_17_[RING-H2]_48</td></tr>
<tr><td></td><td>bdi|Bradi1g39030</td><td>Bd1</td><td>1</td><td>35466765</td><td>35465816</td><td>I</td><td>245</td><td>180_[RING-H2]_18</td></tr>
<tr><td></td><td>bdi|Bradi1g39040</td><td>Bd1</td><td>1</td><td>35470384</td><td>35469620</td><td>E</td><td>167</td><td>100_[RING-H2]_8_[GLD]</td></tr>
<tr><td></td><td>bdi|Bradi2g06840</td><td>Bd2</td><td>-1</td><td>5269600</td><td>5268784</td><td>E</td><td>178</td><td>27_[7]_32_[GLD]_7_[RING-H2]_37</td></tr>
<tr><td></td><td>bdi|Bradi2g06850</td><td>Bd2</td><td>-1</td><td>5275896</td><td>5275279</td><td>E</td><td>205</td><td>103_[GLD]_11_[RING-H2]_32</td></tr>
<tr><td></td><td>bdi|Bradi2g06860</td><td>Bd2</td><td>-1</td><td>5277884</td><td>5277255</td><td>E</td><td>209</td><td>81_[GLD]_10_[RING-H2]_59</td></tr>
<tr><td></td><td>bdi|Bradi2g06870</td><td>Bd2</td><td>-1</td><td>5281804</td><td>5280891</td><td>A</td><td>210</td><td>61_[46]_20_[GLD]_10_[RING-H2]_53</td></tr>
<tr><td></td><td>bdi|Bradi2g06880</td><td>Bd2</td><td>-1</td><td>5286107</td><td>5283528</td><td>A</td><td>451</td><td>82_[GLD]_17_[RING-H2]_175_[GLD]_20_[RING-H2]_39</td></tr>
<tr><td></td><td>bdi|Bradi2g26360</td><td>Bd2</td><td>1</td><td>24832768</td><td>24831368</td><td>G</td><td>203</td><td>61_[GLD]_22_[RING-H2]_61</td></tr>
<tr><td></td><td>bdi|Bradi2g26370</td><td>Bd2</td><td>1</td><td>24845206</td><td>24844640</td><td>E</td><td>188</td><td>23_[7]_16_[60]_21_[GLD]_13_[RING-H2]_33</td></tr>
<tr><td></td><td>bdi|Bradi2g26380</td><td>Bd2</td><td>1</td><td>24852538</td><td>24851807</td><td>I</td><td>243</td><td>178_[RING-H2]_18</td></tr>
<tr><td></td><td>bdi|Bradi2g26390</td><td>Bd2</td><td>1</td><td>24867227</td><td>24866622</td><td>E</td><td>201</td><td>14_[39]_112_[RING-H2]_16</td></tr>
<tr><td></td><td>bdi|Bradi3g09410</td><td>Bd3</td><td>-1</td><td>7527863</td><td>7527204</td><td>E</td><td>219</td><td>103_[GLD]_28_[RING-H2]_29</td></tr>
<tr><td></td><td>bdi|Bradi3g09420</td><td>Bd3</td><td>-1</td><td>7543152</td><td>7542478</td><td>E</td><td>163</td><td>70_[46]_9[GLD]_4_[46]_[RING-H2]_14</td></tr>
<tr><td></td><td>bdi|Bradi3g09430</td><td>Bd3</td><td>1</td><td>7548210</td><td>7547683</td><td>E</td><td>175</td><td>69_[46]_[GLD],EL|98|145|#0000FF|[RING-H2]</td></tr>
<tr><td></td><td>bdi|Bradi3g46030</td><td>Bd3</td><td>-1</td><td>47960985</td><td>47959411</td><td>A</td><td>309</td><td>22_[21]_13_[GLD]_13_[RING-H2]_19_[39]_64_[24]_9_[46]_20</td></tr>
<tr><td></td><td>bdi|Bradi3g46040</td><td>Bd3</td><td>-1</td><td>47967122</td><td>47965964</td><td>A</td><td>318</td><td>91_[GLD]_14_[RING-H2]_89_[24]_49</td></tr>
<tr><td></td><td>bdi|Bradi3g46500</td><td>Bd3</td><td>-1</td><td>48331583</td><td>48330333</td><td>G</td><td>354</td><td>89_[GLD]_1_[20]_4_[RING-H2]_86_[11]_81</td></tr>
<tr><td></td><td>bdi|Bradi3g46510</td><td>Bd3</td><td>-1</td><td>48336003</td><td>48334966</td><td>G</td><td>345</td><td>86_[GLD]_1_[20]_5_[RING-H2]_72_[11]_88</td></tr>
<tr><td></td><td>bdi|Bradi3g56140</td><td>Bd3</td><td>-1</td><td>56168389</td><td>56167943</td><td>H</td><td>148</td><td>1_[79]_23_[GLD]_10_[RING-H2]_24</td></tr>
<tr><td></td><td>bdi|Bradi3g56150</td><td>Bd3</td><td>-1</td><td>56170878</td><td>56170357</td><td>H</td><td>153</td><td>1_[79]_22_[GLD]_13_[RING-H2]_27</td></tr>
<tr><td></td><td>bdi|Bradi3g56160</td><td>Bd3</td><td>-1</td><td>56172688</td><td>56171956</td><td>H</td><td>148</td><td>1_[79]_24_[GLD]_13_[RING-H2]_20</td></tr>
<tr><td></td><td>bdi|Bradi3g56170</td><td>Bd3</td><td>-1</td><td>56174014</td><td>56173428</td><td>H</td><td>147</td><td>2_[81]_[GLD]_7_[RING-H2]_37</td></tr>
<tr><td></td><td>bdi|Bradi5g11870</td><td>Bd5</td><td>1</td><td>15362823</td><td>15361625</td><td>G</td><td>387</td><td>49_[7]_[43]_21_[GLD]_1_[20]_[RING-H2]_24_[39]_15_[76]_[16]_6_[11]_100</td></tr>
<tr><td></td><td>bdi|Bradi5g11880</td><td>Bd5</td><td>-1</td><td>15372139</td><td>15370592</td><td>D</td><td>396</td><td>115_[77]_18_[46]_[25]_1_[GLD]_10_[RING-H2]_60_[60]_15_[40]_64</td></tr>
<tr><td>Oryza sativa</td><td>OsATL2|Os01g11480</td><td>Chr1</td><td>-1</td><td>6182625</td><td>6182125</td><td>E</td><td>166</td><td>71_[GLD]_13_[RING-H2]_23</td></tr>
<tr><td></td><td>OsATL3|Os01g11490</td><td>Chr1</td><td>-1</td><td>6185317</td><td>6184622</td><td>E</td><td>231</td><td>116_[GLD]_10_[RING-H2]_46</td></tr>
<tr><td></td><td>OsATL4|Os01g11500</td><td>Chr1</td><td>-1</td><td>6188219</td><td>6187518</td><td>E</td><td>233</td><td>16_[46]_29_[77]_18_[GLD]_18_[RING-H2]_74</td></tr>
<tr><td></td><td>OsATL14|Os02g14990</td><td>Chr2</td><td>-1</td><td>8363903</td><td>8363274</td><td>E</td><td>209</td><td>50_[46]_24_[39]_34_[46]_5_[RING-H2]_23</td></tr>
<tr><td></td><td>OsATL15|Os02g15000</td><td>Chr2</td><td>-1</td><td>8368327</td><td>8367221</td><td>E</td><td>211</td><td>94_[46]_47_[RING-H2]_16</td></tr>
<tr><td></td><td>OsATL16|Os02g15010</td><td>Chr2</td><td>-1</td><td>8375983</td><td>8375342</td><td>E</td><td>213</td><td>88_[77]_51_[RING-H2]_14</td></tr>
<tr><td></td><td>OsATL17|Os02g15020</td><td>Chr2</td><td>-1</td><td>8381109</td><td>8380540</td><td>E</td><td>189</td><td>74_[GLD]_8_[46]_4_[RING-H2]_36</td></tr>
<tr><td></td><td>OsATL20|Os02g15100</td><td>Chr2</td><td>-1</td><td>8411966</td><td>8411433</td><td>E</td><td>177</td><td>73_[GLD]_19_[46]_[RING-H2]_19</td></tr>
<tr><td></td><td>OsATL21|Os02g15110</td><td>Chr2</td><td>-1</td><td>8417584</td><td>8417084</td><td>E</td><td>166</td><td>95_[GLD]_5_[46]_[RING-H2]_14</td></tr>
<tr><td></td><td>OsATL22|Os02g15120</td><td>Chr2</td><td>1</td><td>8423853</td><td>8422788</td><td>E</td><td>199</td><td>17_[46]_46_[46]_5_[46]_[GLD]_14_[RING-H2]_5_[39]_20</td></tr>
<tr><td></td><td>OsATL27|Os02g36320</td><td>Chr2</td><td>-1</td><td>21918094</td><td>21917048</td><td>G</td><td>348</td><td>87_[46]_2_[GLD]_1_[20]_3_[RING-H2]_6_[39]_57_[11]_80</td></tr>
<tr><td></td><td>OsATL28|Os02g36330</td><td>Chr2</td><td>-1</td><td>21923050</td><td>21921698</td><td>D</td><td>373</td><td>28_[39]_27_[77]_38_[25]_1_[GLD]_10_[RING-H2]_26_[46]_21_[60]_113</td></tr>
<tr><td></td><td>OsATL42|Os03g05560</td><td>Chr3</td><td>-1</td><td>2771430</td><td>2770183</td><td>D</td><td>311</td><td>23_[81]_48_[GLD]_12_[46]_5_[RING-H2]_84_[30]_4_[18]_1</td></tr>
<tr><td></td><td>OsATL43|Os03g05570</td><td>Chr3</td><td>-1</td><td>2776765</td><td>2775608</td><td>D</td><td>300</td><td>23_[34]_8_[46]_33[GLD]_12_[RING-H2]_53_[40]_67</td></tr>
<tr><td></td><td>OsATL51|Os04g37730</td><td>Chr4</td><td>1</td><td>22251753</td><td>22249944</td><td>G</td><td>387</td><td>50_[7]_23_[46]_7_[GLD]_1_[20]_[RING-H2]_5_[39]_12_[39]_13_[76]_[16]_6_[11]_96</td></tr>
<tr><td></td><td>OsATL52|Os04g37740</td><td>Chr4</td><td>-1</td><td>22258590</td><td>22256837</td><td>D</td><td>415</td><td>114_[77]_28_[46]_1_[25]_1_[GLD]_10_[RING-H2]_82_[40]_19_[46]_47</td></tr>
<tr><td></td><td>OsATL129|Os06g34390</td><td>Chr6</td><td>1</td><td>20011039</td><td>20009531</td><td>G</td><td>348</td><td>59_[46]_7_[GLD]_32_[RING-H2]_184</td></tr>
<tr><td></td><td>OsATL77|Os06g34400</td><td>Chr6</td><td>1</td><td>20020320</td><td>20018175</td><td>E</td><td>423</td><td>86_[46]_18_[GLD]_20_[RING-H2]_163_[RING-H2]_23</td></tr>
<tr><td></td><td>OsATL86|Os06g34860</td><td>Chr6</td><td>-1</td><td>20270150</td><td>20269647</td><td>E</td><td>167</td><td>90_[RING-H2]_30</td></tr>
<tr><td></td><td>OsATL87|Os06g34870</td><td>Chr6</td><td>-1</td><td>20280210</td><td>20275504</td><td>E</td><td>357</td><td>99_[RING-H2]_211</td></tr>
<tr><td></td><td>OsATL113|Os11g47690</td><td>Chr11</td><td>1</td><td>28261441</td><td>28260929</td><td>G</td><td>170</td><td>16_[81]_8_[77]_4_[60]_24_[26]_[RING-H2]_27</td></tr>
<tr><td></td><td>OsATL114|Os11g47700</td><td>Chr11</td><td>1</td><td>28262566</td><td>28262054</td><td>G</td><td>170</td><td>66_[46]_11_[46]_13_[26]_[RING-H2]_10</td></tr>
<tr><td>Setaria italica</td><td>sti|SiPROV031834m.g</td><td>scaffold_1</td><td>1</td><td>28328824</td><td>28327250</td><td>G</td><td>360</td><td>52_[7]_18_[GLD]_1_[20]_[RING-H2]_31_[76]_16_[11]_101</td></tr>
<tr><td></td><td>sti|SiPROV013096m.g</td><td>scaffold_1</td><td>-1</td><td>28333002</td><td>28331875</td><td>G</td><td>360</td><td>90_[GLD]_1_[20]_3_[RING-H2]_71_[16]_[11]_90</td></tr>
<tr><td></td><td>sti|SiPROV035554m.g</td><td>scaffold_1</td><td>-1</td><td>28337455</td><td>28335992</td><td>D</td><td>323</td><td>92_[25]_1_[GLD]_9_[RING-H2]_150</td></tr>
<tr><td></td><td>sti|SiPROV034183m.g</td><td>scaffold_1</td><td>-1</td><td>27848852</td><td>27847255</td><td>A</td><td>217</td><td>11_[7]_32_[GLD]_14_[RING-H2]_35_[24]_34</td></tr>
<tr><td></td><td>sti|SiPROV037883m.g</td><td>scaffold_1</td><td>-1</td><td>27851114</td><td>27849828</td><td>A</td><td>313</td><td>102_[GLD]_15_[RING-H2]_78_[24]_43</td></tr>
<tr><td></td><td>sti|SiPROV031464m.g</td><td>scaffold_1</td><td>-1</td><td>1446601</td><td>1445190</td><td>E</td><td>189</td><td>79_[GLD]_15_[RING-H2]_36</td></tr>
<tr><td></td><td>sti|SiPROV032676m.g</td><td>scaffold_1</td><td>1</td><td>1451936</td><td>1451445</td><td>E</td><td>164</td><td>81_[GLD]_4_[46]_1_[RING-H2]_12</td></tr>
<tr><td></td><td>sti|SiPROV034533m.g</td><td>scaffold_1</td><td>1</td><td>1458277</td><td>1457735</td><td>E</td><td>181</td><td>70_[46]_[GLD]_26_[RING-H2]_19</td></tr>
<tr><td></td><td>sti|SiPROV035141m.g</td><td>scaffold_1</td><td>1</td><td>1601776</td><td>1601081</td><td>E</td><td>195</td><td>72_[46]_56_[RING-H2]_13</td></tr>
<tr><td></td><td>sti|SiPROV019612m.g</td><td>scaffold_1</td><td>-1</td><td>1612152</td><td>1611472</td><td>E</td><td>214</td><td>149_[RING-H2]_18</td></tr>
<tr><td></td><td>sti|SiPROV022357m.g</td><td>scaffold_1</td><td>1</td><td>37004299</td><td>37003431</td><td>H</td><td>145</td><td>[79]_20_[GLD]_8_[RING-H2]_27</td></tr>
<tr><td></td><td>sti|SiPROV034669m.g</td><td>scaffold_1</td><td>1</td><td>37010250</td><td>37009858</td><td>H</td><td>122</td><td>54_[GLD]_7_[RING-H2]_2</td></tr>
<tr><td></td><td>sti|SiPROV035133m.g</td><td>scaffold_1</td><td>1</td><td>37023071</td><td>37022622</td><td>H</td><td>150</td><td>1_[79]_42_[RING-H2]_29</td></tr>
<tr><td>Zea mays</td><td>zma|GRMZM2G056786</td><td>1</td><td>-1</td><td>10972234</td><td>10970657</td><td>D</td><td>313</td><td>35_[81]_24_[46]_18_[GLD]_21_[RING-H2]_70_[30]_3_[18]_8</td></tr>
<tr><td></td><td>zma|GRMZM2G056804</td><td>1</td><td>-1</td><td>10978867</td><td>10977780</td><td>D</td><td>302</td><td>22_[34]_8_[46]_33_[GLD]_9_[RING-H2]_57_[40]_69</td></tr>
<tr><td></td><td>zma|GRMZM2G475521</td><td>4</td><td>-1</td><td>1220490</td><td>1219751</td><td>G</td><td>191</td><td>44_[77]_15_[60]_26_[26]_[RING-H2]_31</td></tr>
<tr><td></td><td>zma|GRMZM2G534826</td><td>4</td><td>-1</td><td>1189722</td><td>1189186</td><td>G</td><td>178</td><td>112_[26]_[RING-H2]_10</td></tr>
<tr><td></td><td>zma|GRMZM2G020329</td><td>5</td><td>1</td><td>143167355</td><td>143166651</td><td>E</td><td>204</td><td>148_[RING-H2]_9</td></tr>
<tr><td></td><td>zma|GRMZM2G324111</td><td>5</td><td>1</td><td>143164629</td><td>143163780</td><td>E</td><td>201</td><td>95_[GLD]_18_[RING-H2]_29</td></tr>
<tr><td></td><td>zma|GRMZM2G098925</td><td>5</td><td>1</td><td>143010117</td><td>143009480</td><td>E</td><td>182</td><td>80_[GLD]_9_[46]_9_[RING-H2]_18</td></tr>
<tr><td></td><td>zma|GRMZM2G358711</td><td>5</td><td>1</td><td>143082367</td><td>143081646</td><td>E</td><td>192</td><td>91_[GLD]_13_[RING-H2]_29</td></tr>
<tr><td></td><td>zma|GRMZM2G384913</td><td>5</td><td>-1</td><td>142790793</td><td>142790195</td><td>E</td><td>183</td><td>80_[GLD]_12_[RING-H2]_2_[39]_18</td></tr>
<tr><td></td><td>zma|GRMZM2G163539</td><td>5</td><td>1</td><td>142855057</td><td>142854259</td><td>E</td><td>152</td><td>71_[GLD]_11_[RING-H2]_11</td></tr>
<tr><td></td><td>zma|GRMZM2G480106</td><td>9</td><td>-1</td><td>63126946</td><td>63126104</td><td>E</td><td>216</td><td>51_[7]_[60]_20_[GLD]_24_[RING-H2]_29</td></tr>
<tr><td></td><td>zma|GRMZM2G040236</td><td>9</td><td>-1</td><td>63170056</td><td>63169104</td><td>E</td><td>224</td><td>148_[RING-H2]_29</td></tr>
<tr><td>Sorghum bicolor</td><td>Sbi|Sb01g035920</td><td>chromosome_1</td><td>-1</td><td>59552581</td><td>59552057</td><td>A</td><td>174</td><td>17_[34]_29_[GLD]_17_[RING-H2]_30</td></tr>
<tr><td></td><td>Sbi|Sb01g035930</td><td>chromosome_1</td><td>-1</td><td>59570991</td><td>59570356</td><td>A</td><td>211</td><td>110_[GLD]_21_[RING-H2]_21</td></tr>
<tr><td></td><td>Sbi|Sb01g046930</td><td>chromosome_1</td><td>1</td><td>70026585</td><td>70025632</td><td>D</td><td>317</td><td>31_[34]_48_[GLD]_12_[RING-H2]_56_[40]_73</td></tr>
<tr><td></td><td>Sbi|Sb01g046940</td><td>chromosome_1</td><td>1</td><td>70034908</td><td>70033744</td><td>D</td><td>317</td><td>34_[81]_49_[GLD]_18_[RING-H2]_74_[30]_45</td></tr>
<tr><td></td><td>Sbi|Sb03g001960</td><td>chromosome_3</td><td>1</td><td>1816882</td><td>1813140</td><td>E</td><td>435</td><td>89_[GLD]_8_[46]_7_[RING-H2]_94_[27]_18_[GLD]_21_[RING-H2]_61</td></tr>
<tr><td></td><td>Sbi|Sb03g001970</td><td>chromosome_3</td><td>1</td><td>1821676</td><td>1821206</td><td>E</td><td>156</td><td>78_[GLD]_15_[RING-H2]_4</td></tr>
<tr><td></td><td>Sbi|Sb03g001980</td><td>chromosome_3</td><td>1</td><td>1833897</td><td>1833361</td><td>E</td><td>178</td><td>30_[7]_1_[77]_15_[GLD]_7_[RING-H2]_38</td></tr>
<tr><td></td><td>Sbi|Sb04g008070</td><td>chromosome_4</td><td>1</td><td>8797821</td><td>8796813</td><td>E</td><td>287</td><td>175_[RING-H2]_65</td></tr>
<tr><td></td><td>Sbi|Sb04g008080</td><td>chromosome_4</td><td>1</td><td>8800441</td><td>8799623</td><td>E</td><td>272</td><td>208_[RING-H2]_17</td></tr>
<tr><td></td><td>Sbi|Sb04g008940</td><td>chromosome_4</td><td>-1</td><td>10646097</td><td>10645399</td><td>E</td><td>232</td><td>101_[46]_65_[RING-H2]_12</td></tr>
<tr><td></td><td>Sbi|Sb04g008950</td><td>chromosome_4</td><td>-1</td><td>10671829</td><td>10671311</td><td>E</td><td>172</td><td>81_[46]_25_[RING-H2]_12</td></tr>
<tr><td></td><td>Sbi|Sb04g008960</td><td>chromosome_4</td><td>-1</td><td>10701703</td><td>10701214</td><td>E</td><td>210</td><td>105_[GLD]_19_[RING-H2]_27</td></tr>
<tr><td></td><td>Sbi|Sb04g008970</td><td>chromosome_4</td><td>-1</td><td>10703617</td><td>10702946</td><td>E</td><td>223</td><td>145_[RING-H2]_13</td></tr>
<tr><td></td><td>Sbi|Sb04g008980</td><td>chromosome_4</td><td>-1</td><td>10719737</td><td>10718952</td><td>E</td><td>261</td><td>182_[RING-H2]_32</td></tr>
<tr><td></td><td>Sbi|Sb04g008990</td><td>chromosome_4</td><td>-1</td><td>10727615</td><td>10726750</td><td>E</td><td>240</td><td>22_[46]_78_[GLD]_18_[RING-H2]_56</td></tr>
<tr><td></td><td>Sbi|Sb04g009000</td><td>chromosome_4</td><td>-1</td><td>10756331</td><td>10755792</td><td>E</td><td>179</td><td>53_[46]_17_[GLD]_9_[46]_9_[RING-H2]_18</td></tr>
<tr><td></td><td>Sbi|Sb04g009010</td><td>chromosome_4</td><td>-1</td><td>10785783</td><td>10784112</td><td>E</td><td>166</td><td>28_[81]_34_[GLD]_13_[RING-H2]_16</td></tr>
<tr><td></td><td>Sbi|Sb04g009020</td><td>chromosome_4</td><td>1</td><td>10801171</td><td>10800584</td><td>E</td><td>195</td><td>12_[39]_60_[GLD]_16_[RING-H2]_34</td></tr>
<tr><td></td><td>Sbi|Sb04g009320</td><td>chromosome_4</td><td>1</td><td>11612860</td><td>11612198</td><td>E</td><td>220</td><td>140_[RING-H2]_33</td></tr>
<tr><td></td><td>Sbi|Sb04g009330</td><td>chromosome_4</td><td>1</td><td>11618866</td><td>11618189</td><td>E</td><td>225</td><td>22_[46]_76_[GLD]_28_[RING-H2]_33</td></tr>
<tr><td></td><td>Sbi|Sb04g023060</td><td>chromosome_4</td><td>-1</td><td>52725976</td><td>52724917</td><td>A</td><td>260</td><td>59_[GLD]_16_[RING-H2]_82_[24]_27</td></tr>
<tr><td></td><td>Sbi|Sb04g023070</td><td>chromosome_4</td><td>-1</td><td>52730600</td><td>52729239</td><td>A</td><td>308</td><td>86_[46]_8_[GLD]_18_[RING-H2]_86_[24]_28</td></tr>
<tr><td></td><td>Sbi|Sb04g023620</td><td>chromosome_4</td><td>-1</td><td>53293107</td><td>53292028</td><td>G</td><td>359</td><td>87_[GLD]_1_[20]_4_[RING-H2]_6_[39]_67_[11]_89</td></tr>
<tr><td></td><td>Sbi|Sb04g023630</td><td>chromosome_4</td><td>-1</td><td>53297996</td><td>53296238</td><td>D</td><td>363</td><td>112_[25]_1_[GLD]_10_[RING-H2]_169</td></tr>
<tr><td></td><td>Sbi|Sb04g029050</td><td>chromosome_4</td><td>-1</td><td>59083770</td><td>59083336</td><td>H</td><td>144</td><td>51_[GLD]_3_[46]_[RING-H2]_24</td></tr>
<tr><td></td><td>Sbi|Sb04g029060</td><td>chromosome_4</td><td>-1</td><td>59089281</td><td>59088808</td><td>H</td><td>157</td><td>1_[79]_20_[GLD]_12_[RING-H2]_34</td></tr>
<tr><td></td><td>Sbi|Sb05g027490</td><td>chromosome_5</td><td>1</td><td>61870974</td><td>61870096</td><td>G</td><td>195</td><td>44_[77]_[60]_43_[26]_[RING-H2]_33</td></tr>
<tr><td></td><td>Sbi|Sb05g027500</td><td>chromosome_5</td><td>1</td><td>61874379</td><td>61873828</td><td>G</td><td>183</td><td>112_[26]_1_[RING-H2]_14</td></tr>
<tr><td></td><td>Sbi|Sb05g027540</td><td>chromosome_5</td><td>1</td><td>61960210</td><td>61959662</td><td>G</td><td>182</td><td>115_[26]_1_[RING-H2]_10</td></tr>
<tr><td></td><td>Sbi|Sb05g027550</td><td>chromosome_5</td><td>1</td><td>61972867</td><td>61972130</td><td>G</td><td>245</td><td>178_[26]_[RING-H2]_11</td></tr>
<tr><td></td><td>Sbi|Sb05g027560</td><td>chromosome_5</td><td>1</td><td>61974969</td><td>61974424</td><td>G</td><td>181</td><td>114_[26]_1_[RING-H2]_10</td></tr>
<tr><td></td><td>Sbi|Sb05g027570</td><td>chromosome_5</td><td>1</td><td>62003461</td><td>62002661</td><td>A</td><td>266</td><td>192_[26]_[RING-H2]_18</td></tr>
<tr><td></td><td>Sbi|Sb05g027580</td><td>chromosome_5</td><td>1</td><td>62010129</td><td>62009458</td><td>A</td><td>223</td><td>149_[26]_[RING-H2]_18</td></tr>
<tr><td></td><td>Sbi|Sb08g002625</td><td>chromosome_8</td><td>-1</td><td>2729858</td><td>2729517</td><td>C</td><td>162</td><td>15_[4]_22_[GLD]_2_[RING-H2]_33</td></tr>
<tr><td></td><td>Sbi|Sb08g002630</td><td>chromosome_8</td><td>-1</td><td>2742800</td><td>2742162</td><td>C</td><td>212</td><td>50_[4]_28_[GLD]_10_[RING-H2]_34</td></tr>
<tr><td></td><td>Sbi|Sb09g017880</td><td>chromosome_9</td><td>1</td><td>44639115</td><td>44638243</td><td>A</td><td>206</td><td>124_[46]_[RING-H2]_28</td></tr>
<tr><td></td><td>Sbi|Sb09g017890</td><td>chromosome_9</td><td>1</td><td>44652305</td><td>44650876</td><td>A</td><td>223</td><td>44_[7]_65_[46]_8_[RING-H2]_36</td></tr>
<tr><td></td><td>Sbi|Sb10g020965</td><td>chromosome_10</td><td>1</td><td>45989830</td><td>45987143</td><td>G</td><td>252</td><td>65_[GLD]_25_[RING-H2]_102</td></tr>
<tr><td></td><td>Sbi|Sb10g020970</td><td>chromosome_10</td><td>1</td><td>45992217</td><td>45991018</td><td>G</td><td>328</td><td>74_[GLD]_32_[RING-H2]_163</td></tr>
<tr><td></td><td>Sbi|Sb10g020980</td><td>chromosome_10</td><td>1</td><td>46066033</td><td>46065356</td><td>E</td><td>225</td><td>149_[RING-H2]_29</td></tr>
<tr><td></td><td>Sbi|Sb10g020990</td><td>chromosome_10</td><td>1</td><td>46075992</td><td>46075234</td><td>E</td><td>212</td><td>74_[46]_3_[60]_10_[GLD]_24_[RING-H2]_27</td></tr>
<tr><td></td><td>Sbi|Sb10g021070</td><td>chromosome_10</td><td>1</td><td>46383406</td><td>46382915</td><td>E</td><td>163</td><td>73_[GLD]_9_[RING-H2]_22</td></tr>
<tr><td></td><td>Sbi|Sb10g021077</td><td>chromosome_10</td><td>1</td><td>46444645</td><td>46444190</td><td>E</td><td>159</td><td>4_[39]_92_[RING-H2]_4</td></tr>
<tr><td></td><td>Sbi|Sb10g021080</td><td>chromosome_10</td><td>1</td><td>46457491</td><td>46457000</td><td>E</td><td>163</td><td>97_[RING-H2]_19</td></tr>
<tr><td></td><td>Sbi|Sb10g021090</td><td>chromosome_10</td><td>1</td><td>46510345</td><td>46509701</td><td>E</td><td>214</td><td>8_[39]_131[RING-H2]_16</td></tr>
<tr><td>Aquilegia coerulea</td><td>aco|AcoGoldSmith_v1.005679m.g</td><td>scaffold_20</td><td>1</td><td>2425281</td><td>2424000</td><td>G</td><td>421</td><td>44_[22]_22_[GLD]_[63]_1_[RING-H2]_79_[48]_[17]_117_[28]_14</td></tr>
<tr><td></td><td>aco|AcoGoldSmith_v1.024946m.g</td><td>scaffold_20</td><td>1</td><td>2428687</td><td>2427371</td><td>G</td><td>438</td><td>46_[22]_27_[GLD]_[63]_1_[RING-H2]_98_[17]_25_[57]_38_[67]_63</td></tr>
<tr><td></td><td>aco|AcoGoldSmith_v1.013754m.g</td><td>scaffold_27</td><td>1</td><td>2732889</td><td>2732365</td><td>B</td><td>174</td><td>1_[68]_[7]_60_[RING-H2]_38</td></tr>
<tr><td></td><td>aco|AcoGoldSmith_v1.017218m.g</td><td>scaffold_27</td><td>1</td><td>2727595</td><td>2727021</td><td>B</td><td>156</td><td>16_[81]_45_[26]_2_[RING-H2]_21</td></tr>
<tr><td></td><td>aco|AcoGoldSmith_v1.025500m.g</td><td>scaffold_27</td><td>1</td><td>2734390</td><td>2733950</td><td>B</td><td>147</td><td>29_[81]_38_[26]_5_[RING-H2]_3</td></tr>
<tr><td></td><td>aco|AcoGoldSmith_v1.011994m.g</td><td>scaffold_5</td><td>-1</td><td>5643971</td><td>5639891</td><td>F</td><td>134</td><td>5_[34]_40_[RING-H2]_20</td></tr>
<tr><td></td><td>aco|AcoGoldSmith_v1.021743m.g</td><td>scaffold_5</td><td>-1</td><td>5652901</td><td>5652551</td><td>F</td><td>139</td><td>72_[RING-H2]_20</td></tr>
<tr><td></td><td>aco|AcoGoldSmith_v1.020546m.g</td><td>scaffold_9</td><td>-1</td><td>3957385</td><td>3956900</td><td>I</td><td>162</td><td>19_[34]_28_[GLD]_12_[RING-H2]_[14]</td></tr>
<tr><td></td><td>aco|AcoGoldSmith_v1.016895m.g</td><td>scaffold_9</td><td>-1</td><td>3958749</td><td>3958255</td><td>I</td><td>164</td><td>20_[34]_28_[GLD]_12_[RING-H2]_[14]_1</td></tr>
<tr><td></td><td>aco|AcoGoldSmith_v1.021001m.g</td><td>scaffold_9</td><td>-1</td><td>3960198</td><td>3959668</td><td>I</td><td>176</td><td>72_[GLD]_12_[RING-H2]_[14]_11</td></tr>
</table>

| | | | | | | | | |
|---|---|---|---|---|---|---|---|---|
| *Mimulus guttatus* | mgu\|mgv1a007689m.g | scaffold_228 | -1 | 82080 | 80797 | D | 398 | 13_[39]_[39]_16_[7]_[27]_3_[10]_[GLD]_[47]_1_[RING-H2]_49_[23]_8_[46]_19_[55]_26_[12]_43_[28]_12 |
| | mgu\|mgv1a014680m.g | scaffold_228 | -1 | 79219 | 78411 | H | 181 | 33_[79]_35_[RING-H2]_35 |
| | mgu\|mgv1a015778m.g | scaffold_8 | 1 | 1763474 | 1762587 | I | 146 | 93_[GLD]_10_[46]_[RING-H2]_21 |
| | mgu\|mgv1a024718m.g | scaffold_8 | 1 | 1765541 | 1764127 | I | 117 | 110_[RING-H2]_7 |
| | mgu\|mgv1a018404m.g | scaffold_8 | 1 | 1766460 | 1765963 | I | 165 | 88_[20]_[RING-H2]_17 |
| | mgu\|mgv1a026696m.g | scaffold_8 | 1 | 1767606 | 1767112 | I | 164 | 85_[20]_2_[RING-H2]_18 |
| | mgu\|mgv1a017977m.g | scaffold_8 | 1 | 1768824 | 1768354 | I | 157 | 90_[20]_2_[RING-H2]_6 |
| | mgu\|mgv1a023344m.g | scaffold_85 | 1 | 1045518 | 1045159 | F | 119 | 6_[81]_47_[RING-H2]_3 |
| | mgu\|mgv1a017791m.g | scaffold_85 | 1 | 1047386 | 1047027 | F | 120 | 71_[RING-H2]_2 |
| *Vitis vinifera* | vvi\|GSVIVG01008754001 | chr18 | 1 | 1721614 | 1721216 | H | 133 | 2_[44]_17_[GLD]_8_[RING-H2]_15 |
| | vvi\|GSVIVG01008756001 | chr18 | -1 | 1728333 | 1727184 | H | 163 | 33_[44]_17_[GLD]_[RING-H2]_15 |
| *Eucalyptus grandis* | egr\|Egrandis_v1_0.040992m.g | scaffold_10 | 1 | 3020156 | 3019707 | I | 150 | 8_[34]_28_[GLD]_9_[RING-H2]_[14]_2 |
| | egr\|Egrandis_v1_0.052297m.g | scaffold_10 | 1 | 3024682 | 3024173 | I | 170 | 78_[GLD]_9[RING-H2]_[14]_2 |
| | egr\|Egrandis_v1_0.039538m.g | scaffold_10 | 1 | 3037524 | 3037249 | I | 138 | 64_[RING-H2]_4_[14]_1 |
| | egr\|Egrandis_v1_0.048044m.g | scaffold_10 | 1 | 31722579 | 31722232 | A | 151 | 12_[34]_22_[RING-H2]_48 |
| | egr\|Egrandis_v1_0.049275m.g | scaffold_10 | 1 | 31726762 | 31725845 | A | 306 | 14_[8]_42_[GLD]_9_[RING-H2]_42_[29]_67_[24] |
| | egr\|Egrandis_v1_0.013326m.g | scaffold_3 | 1 | 71316132 | 71314816 | G | 431 | 90_[GLD]_[63]_1_[RING-H2]_80_[48]_[17]_28_[57]_32_[67]_31_[28]_19 |
| | egr\|Egrandis_v1_0.040468m.g | scaffold_3 | 1 | 71321835 | 71320648 | G | 396 | 1_[68]_[22]_24_[GLD]_[63]_1_[RING-H2]_77_[48]_[17]_28_[57]_32_[67]_31_[28]_19 |
| | egr\|Egrandis_v1_0.026696m.g | scaffold_6 | 1 | 45353111 | 45351565 | F | 211 | 26_[6]_[5]_[RING-H2]_5 |
| | egr\|Egrandis_v1_0.039515m.g | scaffold_6 | 1 | 45358987 | 45357660 | F | 168 | 12_[7]_[6]_[RING-H2]_8 |
| | egr\|Egrandis_v1_0.054012m.g | scaffold_6 | 1 | 5012362 | 5011460 | D | 301 | 73_[7]_37_[25]_1_[GLD]_[26]_1_[RING-H2]_22_[46]_64 |
| | egr\|Egrandis_v1_0.013794m.g | scaffold_6 | 1 | 5024800 | 5022557 | G | 421 | 57_[7]_[43]_17_[GLD]_1_[20]_[RING-H2]_83_[16]_2_[11]_124 |
| | egr\|Egrandis_v1_0.039779m.g | scaffold_7 | -1 | 44904013 | 44903522 | I | 164 | 28_[7]_29_[GLD]_12_[RING-H2]_20 |
| | egr\|Egrandis_v1_0.050415m.g | scaffold_7 | -1 | 44911488 | 44910997 | I | 164 | 22_[34]_29_[GLD]_12_[RING-H2]_20 |
| | egr\|Egrandis_v1_0.040560m.g | scaffold_7 | -1 | 44917807 | 44917445 | I | 121 | 18_[34]_10_[GLD]_12_[RING-H2] |
| | egr\|Egrandis_v1_0.040222m.g | scaffold_9 | 1 | 4881651 | 4880995 | C | 219 | 56_[4]_20_[GLD]_11_[RING-H2]_[49]_30 |
| | egr\|Egrandis_v1_0.025743m.g | scaffold_9 | 1 | 4895178 | 4894259 | C | 226 | 52_[4]_20_[GLD]_11_[RING-H2]_[49]_41 |
| *Citrus clementina* | ccl\|clementine0.9_011644m.g | scaffold_12 | -1 | 4185900 | 4184133 | I | 411 | 85_[15]_1_[65]_65_[45]_31_[34]_37_[GLD]_[53]_[RING-H2]_[14]_8 |
| | ccl\|clementine0.9_034929m.g | scaffold_12 | -1 | 4189625 | 4187572 | I | 231 | 27_[45]_34_[34]_32_[GLD]_[53]_[RING-H2]_29 |
| | ccl\|clementine0.9_033273m.g | scaffold_24 | 1 | 219129 | 218536 | C | 198 | 48_[4]_21_[GLD]_11_[RING-H2]_[49]_16 |
| | ccl\|clementine0.9_021416m.g | scaffold_24 | 1 | 236914 | 235547 | C | 219 | 100_[GLD]_11_[RING-H2]_[49]_18_[72]_3 |
| | ccl\|clementine0.9_021420m.g | scaffold_24 | 1 | 242017 | 240777 | C | 219 | 48_[4]_21_[GLD]_11_[RING-H2]_[49]_18_[72]_3 |
| | ccl\|clementine0.9_032013m.g | scaffold_24 | 1 | 263079 | 262468 | C | 218 | 48_[4]_20_[GLD]_11_[RING-H2]_[49]_19_[72]_2 |
| | ccl\|clementine0.9_020692m.g | scaffold_24 | 1 | 294198 | 293493 | C | 232 | 52_[4]_21_[GLD]_11_[RING-H2]_[49]_22_[72]_8 |
| | ccl\|clementine0.9_031204m.g | scaffold_24 | -1 | 312582 | 311950 | C | 211 | 46_[4]_20_[GLD]_11_[RING-H2]_[49]_32 |
| | ccl\|clementine0.9_035169m.g | scaffold_24 | 1 | 327629 | 326997 | C | 256 | 86_[4]_20_[GLD]_11_[RING-H2]_[49]_19_[72]_2 |
| *Citrus sinensis* | csi\|orange1.1g028342m.g | scaffold00143 | 1 | 286805 | 285788 | C | 211 | 46_[4]_20_[GLD]_11_[RING-H2]_[49]_32 |
| | csi\|orange1.1g042990m.g | scaffold00143 | -1 | 271775 | 271278 | C | 166 | 46_[GLD]_11_[RING-H2]_[49]_19_[72]_3 |
| | csi\|orange1.1g027813m.g | scaffold00143 | -1 | 355384 | 354207 | C | 219 | 48_[4]_21_[GLD]_11_[RING-H2]_[49]_18_[72]_3 |
| | csi\|orange1.1g046833m.g | scaffold00143 | -1 | 359776 | 359120 | C | 219 | 18_[71]_66_[GLD]_11_[RING-H2]_[49]_18_[72]_3 |
| | csi\|orange1.1g035583m.g | scaffold00047 | 1 | 26153 | 25680 | H | 165 | 112_[RING-H2]_6 |
| | csi\|orange1.1g042258m.g | scaffold00047 | 1 | 29778 | 29320 | H | 189 | 16_[78]_84_[RING-H2]_11 |
| *Carica papaya* | cpa\|evm.TU.supercontig_2.424 | supercontig_2 | 1 | 4515896 | 4515402 | F | 164 | 79_[GLD]_18_[RING-H2]_8 |
| | cpa\|evm.TU.supercontig_2.425 | supercontig_2 | -1 | 4522821 | 4521946 | A | 291 | 23_[8]_[77]_35_[GLD]_[RING-H2]_96_[24]_15 |
| | cpa\|evm.TU.supercontig_42.96 | supercontig_42 | -1 | 918578 | 917883 | C | 231 | 39_[4]_22_[GLD]_11_[RING-H2]_[49]_57 |
| | cpa\|evm.TU.supercontig_42.97 | supercontig_42 | -1 | 921382 | 920819 | C | 187 | 14_[71]_12_[4]_18_[GLD]_11_[RING-H2]_26 |
| | cpa\|evm.TU.supercontig_80.56 | supercontig_80 | 1 | 460332 | 459889 | H | 147 | 97_[RING-H2]_3 |
| | cpa\|evm.TU.supercontig_80.57 | supercontig_80 | 1 | 462421 | 461828 | F | 197 | 33_[7]_96_[RING-H2]_5 |
| | cpa\|evm.TU.supercontig_81.149 | supercontig_81 | -1 | 727433 | 726927 | F | 168 | 6_[39]_77_[RING-H2]_26 |
| | cpa\|evm.TU.supercontig_81.150 | supercontig_81 | -1 | 728648 | 728079 | F | 189 | 124_[RING-H2]_18 |
| | cpa\|evm.TU.supercontig_81.151 | supercontig_81 | -1 | 729971 | 729366 | F | 201 | 116_[20]_1_[RING-H2]_25 |
| | cpa\|evm.TU.supercontig_81.152 | supercontig_81 | -1 | 731369 | 730902 | F | 155 | 12_[39]_81_[RING-H2]_3 |
| *Arabidopsis thaliana* | ATL75\|At1g49200 | Chr1 | 1 | 18199168 | 18198227 | C | 226 | 21_[71]_18_[4]_22_[GLD]_11_[RING-H2]_[49]_18_[72]_2 |
| | ATL76\|At1g49210 | Chr1 | 1 | 18202764 | 18201889 | C | 225 | 20_[71]_18_[4]_22_[GLD]_11_[RING-H2]_[49]_18_[72]_2 |
| | ATL10\|At1g49220 | Chr1 | 1 | 18206701 | 18205934 | C | 251 | 20_[71]_18_[4]_22_[GLD]_11_[RING-H2]_[49]_44_[72]_2 |
| | ATL78\|At1g49230 | Chr1 | 1 | 18210128 | 18209261 | C | 219 | 24_[71]_12_[4]_20_[GLD]_11_[RING-H2]_[49]_34 |
| | ATL40\|At2g42350 | Chr2 | 1 | 17639998 | 17639152 | A | 217 | 24_[8]_25_[GLD]_9_[RING-H2]_39_[24]_20 |
| | ATL41\|At2g42360 | Chr2 | 1 | 17641790 | 17640798 | A | 236 | 24_[8]_28_[GLD]_11_[RING-H2]_50_[24]_20 |
| | ATL38\|At2g34990 | Chr2 | -1 | 14751168 | 14750260 | G | 302 | 66_[GLD]_1_[20]_[RING-H2]_56_[16]_[11]_5_[36]_47 |
| | ATL9\|At2g35000 | Chr2 | -1 | 14753319 | 14751574 | G | 378 | 105_[GLD]_1_[20]_[RING-H2]_74_[16]_[11]_8_[36]_63 |
| | ATL89\|AT2G44578 | Chr2 | -1 | 18401326 | 18400646 | A | 145 | 65_[RING-H2]_33 |
| | ATL90\|AT2G44581 | Chr2 | -1 | 18398433 | 18397996 | A | 145 | 65_[RING-H2]_33 |
| | ATL77\|At3g18773 | Chr3 | 1 | 6467175 | 6465825 | C | 220 | 21_[71]_11_[4]_23_[GLD]_11_[RING-H2]_[49]_18_[72]_2 |
| | ATL81\|At3g18777 | Chr3 | -1 | 6525543 | 6523530 | C | 222 | 22_[71]_22_[4]_19_[GLD]_11_[RING-H2]_[49]_30 |
| | ATL39\|At4g09100 | Chr4 | 1 | 5813396 | 5812488 | G | 132 | 13_[7]_26_[GLD]_13_[RING-H2]_5 |
| | ATL35\|At4g09110 | Chr4 | 1 | 5815035 | 5813998 | G | 302 | 93_[GLD]_1_[20]_[RING-H2]_31_[11]_8_[36]_54 |
| | ATL36\|At4g09120 | Chr4 | 1 | 5816922 | 5815849 | G | 345 | 93_[GLD]_1_[20]_[RING-H2]_56_[16]_1_[11]_8_[36]_59 |
| | ATL7\|At4g10150 | Chr4 | 1 | 6329589 | 6328136 | F | 236 | 16_[21]_19_[GLD]_9_[RING-H2]_83 |
| | ATL59\|At4g10160 | Chr4 | 1 | 6337332 | 6336023 | F | 225 | 71_[GLD]_9_[RING-H2]_86 |
| | ATL85\|AT5G41430 | Chr5 | -1 | 16586604 | 16586119 | F | 161 | 9_[81]_39_[39]_36_[RING-H2]_2 |
| | ATL86\|AT5G41440 | Chr5 | -1 | 16587609 | 16587235 | F | 124 | 34_[39]_27_[RING-H2]_4 |
| | ATL87\|AT5G41450 | Chr5 | -1 | 16589094 | 16588600 | F | 164 | 105_[RING-H2]_12 |
| *Arabidopsis lyrata* | aly\|474062 | scaffold_1 | 1 | 25169209 | 25170126 | C | 226 | 21_[71]_18_[4]_22_[GLD]_11_[RING-H2]_[49]_18_[72]_2 |
| | aly\|474063 | scaffold_1 | 1 | 25183327 | 25184109 | C | 225 | 20_[71]_18_[4]_22_[GLD]_11_[RING-H2]_[49]_18_[72]_2 |
| | aly\|474064 | scaffold_1 | 1 | 25193315 | 25193996 | C | 225 | 20_[71]_18_[4]_22_[GLD]_11_[RING-H2]_[49]_18_[72]_2 |
| | aly\|479387 | scaffold_3 | 1 | 8074087 | 8075268 | C | 222 | 22_[71]_22_[4]_19_[GLD]_11_[RING-H2]_[49]_30 |
| | aly\|929765 | scaffold_3 | 1 | 8069655 | 8070350 | C | 217 | 21_[71]_11_[4]_23_[GLD]_11_[RING-H2]_[49]_33 |
| | aly\|483328 | scaffold_4 | 1 | 20681392 | 20682202 | A | 217 | 21_[8]_26_[GLD]_9_[RING-H2]_38_[24]_20 |
| | aly\|483329 | scaffold_4 | 1 | 20682962 | 20683936 | A | 236 | 24_[8]_28_[GLD]_11_[RING-H2]_50_[24]_20 |
| | aly\|935395 | scaffold_4 | -1 | 21733655 | 21734133 | A | 145 | 65_[RING-H2]_33 |
| | aly\|935396 | scaffold_4 | -1 | 21736439 | 21737126 | A | 157 | 15_[7]_34_[RING-H2]_45 |
| | aly\|942690 | scaffold_6 | 1 | 19159491 | 19159935 | G | 133 | 55_[GLD]_13_[RING-H2]_6 |
| | aly\|489785 | scaffold_6 | 1 | 19161672 | 19162429 | G | 259 | 93_[GLD]_1_[20]_[RING-H2]_33_[11]_8_[36]_9 |
| | aly\|942692 | scaffold_6 | 1 | 19162956 | 19164048 | G | 350 | 93_[GLD]_1_[20]_[RING-H2]_59_[16]_[11]_8_[36]_62 |
| | aly\|942695 | scaffold_6 | 1 | 19177864 | 19178866 | G | 320 | 93_[GLD]_1_[20]_[RING-H2]_65_[16]_[11]_8_[36]_26 |
| | aly\|942696 | scaffold_6 | 1 | 19179831 | 19180938 | G | 355 | 88_[GLD]_1_[20]_[RING-H2]_69_[16]_[11]_8_[36]_62 |
| *Prunus persica* | ppe\|ppa015864m.g | scaffold_4 | -1 | 10808087 | 10807821 | H | 230 | 177_[RING-H2]_6 |
| | ppe\|ppa020248m.g | scaffold_4 | -1 | 10810495 | 10810193 | H | 255 | 202_[RING-H2]_6 |
| | ppe\|ppa007165m.g | scaffold_5 | -1 | 11972469 | 11971088 | I | 379 | 57_[64]_9_[15]_87_[45]_33_[34]_29_[GLD]_[53]_[RING-H2]_15 |
| | ppe\|ppa025741m.g | scaffold_5 | -1 | 11974262 | 11973053 | I | 367 | 46_[64]_13_[15]_3_[65]_60_[45]_81_[GLD]_[53]_[RING-H2]_15 |
| | ppe\|ppa018265m.g | scaffold_5 | -1 | 11996309 | 11994153 | I | 358 | 49_[64]_12_[15]_91_[45]_20_[34]_32_[GLD]_[53]_[RING-H2]_5 |
| | ppe\|ppb024146m.g | scaffold_5 | -1 | 12000251 | 11998844 | I | 388 | 54_[64]_11_[15]_88_[45]_38_[34]_32_[GLD]_[53]_[RING-H2]_16 |
| | ppe\|ppa007460m.g | scaffold_5 | -1 | 12002302 | 12000234 | I | 367 | 54_[64]_11_[15]_86_[45]_33_[34]_29_[GLD]_[53]_[RING-H2]_5 |
| | ppe\|ppa024005m.g | scaffold_5 | -1 | 12009849 | 12008447 | I | 365 | 53_[64]_11_[15]_83_[45]_33_[34]_30_[GLD]_[53]_[RING-H2]_6 |
| | ppe\|ppa019102m.g | scaffold_5 | -1 | 12012642 | 12010103 | I | 403 | 51_[64]_14_[15]_103_[45]_31_[34]_30_[GLD]_[53]_[RING-H2]_25 |
| | ppe\|ppa024544m.g | scaffold_6 | 1 | 1439362 | 1438547 | A | 272 | 6_[60]_12_[8]_53_[GLD]_19_[RING-H2]_68_[24]_4 |
| | ppe\|ppa016359m.g | scaffold_6 | -1 | 1442500 | 1442018 | F | 160 | 13_[80]_33_[GLD]_18_[RING-H2]_9 |
| | ppe\|ppa027038m.g | scaffold_6 | 1 | 22825689 | 22825240 | B | 150 | 15_[81]_55_[RING-H2]_17 |
| | ppe\|ppa024559m.g | scaffold_6 | 1 | 22827272 | 22826727 | B | 181 | 9_[39]_9_[81]_37_[20]_3_[RING-H2]_36 |
| | ppe\|ppa019638m.g | scaffold_7 | 1 | 20341765 | 20340509 | G | 418 | 42_[22]_23_[GLD]_[63]_1_[RING-H2]_82_[48]_[17]_25_[57]_37_[67]_27_[28]_9 |
| | ppe\|ppa026744m.g | scaffold_7 | 1 | 20343957 | 20342731 | G | 441 | 62_[22]_23_[GLD]_[63]_1_[RING-H2]_82_[48]_[17]_28_[57]_37_[67]_27_[28]_9 |
| *Cucumis sativis* | csa\|Cucsa.089780 | scaffold00894 | -1 | 1166549 | 1165935 | H | 178 | 13_[39]_9_[7]_12_[77]_31_[RING-H2]_26 |
| | csa\|Cucsa.089790 | scaffold00894 | -1 | 1174142 | 1172141 | D | 430 | 37_[7]_[27]_8_[10]_[GLD]_[47]_2_[RING-H2]_65_[23]_5_[9]_1_[75]_13_[55]_77_[28]_21 |
| | csa\|Cucsa.261420 | scaffold02259 | 1 | 13988 | 12720 | G | 422 | 40_[22]_25_[GLD]_[63]_1_[RING-H2]_81_[48]_[17]_28_[57]_35_[67]_26_[28]_14 |
| | csa\|Cucsa.261430 | scaffold02259 | 1 | 16679 | 15160 | G | 433 | 39_[22]_23_[GLD]_[63]_1_[RING-H2]_40_[60]_40_[48]_[17]_28_[57]_34_[67]_33_[28]_16 |
| *Glycine max* | gma\|Glyma01g02130 | Gm01 | 1 | 1643423 | 1642631 | G | 288 | 22_[39]_45_[GLD]_1_[20]_2_[RING-H2]_53_[16]_3_[11]_45 |
| | gma\|Glyma01g02140 | Gm01 | -1 | 1649811 | 1648375 | D | 352 | 6_[56]_[13]_6_[68]_[7]_[52]_29_[GLD]_[26]_1_[RING-H2]_89_[40]_63 |
| | gma\|Glyma02g37330 | Gm02 | 1 | 42657190 | 42655601 | G | 386 | 55_[22]_28_[GLD]_1_[20]_[RING-H2]_97_[11]_90 |
| | gma\|Glyma02g37340 | Gm02 | 1 | 42671833 | 42670682 | G | 344 | 39_[39]_9_[7]_[43]_16_[GLD]_1_[20]_1_[RING-H2]_43_[16]_[11]_11_[36]_8_[70]_33 |
| | gma\|Glyma09g38870 | Gm09 | 1 | 44117210 | 44116384 | I | 186 | 76_[GLD]_15_[RING-H2]_36 |
| | gma\|Glyma09g38880 | Gm09 | 1 | 44124328 | 44123366 | I | 184 | 19_[34]_20_[73]_6_[GLD]_13_[RING-H2]_[14]_7 |
| | gma\|Glyma11g27880 | Gm11 | 1 | 27796135 | 27795410 | A | 228 | 5_[8]_10_[77]_31_[GLD]_17_[RING-H2]_66 |
| | gma\|Glyma11g27890 | Gm11 | -1 | 27816253 | 27815675 | F | 149 | 88_[RING-H2]_14 |
| | gma\|Glyma13g01460 | Gm13 | -1 | 1169550 | 1159475 | H | 202 | 98_[GLD]_10_[RING-H2]_35 |
| | gma\|Glyma13g01470 | Gm13 | -1 | 1177983 | 1176209 | D | 520 | 49_[7]_[27]_3_[10]_[GLD]_[47]_2_[RING-H2]_90_[23]_3_[46]_2_[9]_1_[75]_13_[55]_38_[12]_111 |
| | gma\|Glyma17g07580 | Gm17 | -1 | 5583220 | 5582495 | H | 177 | 73_[GLD]_10_[RING-H2]_35 |
| | gma\|Glyma17g07590 | Gm17 | -1 | 5591168 | 5589140 | D | 512 | 35_[7]_[27]_3_[10]_[GLD]_[47]_2_[RING-H2]_92_[23]_3_[46]_1_[9]_1_[75]_13_[55]_40_[12]_114 |
| | gma\|Glyma18g01790 | Gm18 | -1 | 1014009 | 1013607 | D | 169 | 64_[25]_1_[GLD]_[26]_3_[RING-H2]_21 |
| | gma\|Glyma18g01800 | Gm18 | -1 | 1018353 | 1017658 | D | 387 | 40_[56]_18_[7]_31_[25]_1_[GLD]_[26]_1_[RING-H2]_85_[40]_87 |
| *Medicago truncatula* | mtr\|Medtr7g089020 | MtChr7 | -1 | 19466669 | 19464440 | I | 203 | 14_[34]_23_[73]_4_[GLD]_12_[RING-H2]_[14]_31 |
| | mtr\|Medtr7g089030 | MtChr7 | -1 | 19473081 | 19472593 | I | 162 | 73_[GLD]_14_[RING-H2]_16 |
| *Populus trichocarpa* | pop\|POPTR_0001s31710 | scaffold_1 | -1 | 30075580 | 30074897 | C | 225 | 18_[71]_24_[4]_19_[GLD]_11_[RING-H2]_[49]_19_[72] |
| | pop\|POPTR_0001s31720 | scaffold_1 | -1 | 30107918 | 30107256 | C | 220 | 18_[71]_25_[4]_10_[GLD]_12_[RING-H2]_[49]_19_[72]_2 |
| | pop\|POPTR_0008s01970 | scaffold_8 | 1 | 941941 | 941166 | I | 156 | 69_[GLD]_1_[20]_2_[RING-H2]_13 |
| | pop\|POPTR_0008s01980 | scaffold_8 | 1 | 946192 | 944528 | I | 164 | 18_[34]_29_[GLD]_1_[20]_4_[RING-H2]_19 |
| | pop\|POPTR_0010s24950 | scaffold_10 | 1 | 20887461 | 20886202 | I | 211 | 17_[34]_30_[GLD]_1_[20]_2_[RING-H2]_68 |
| | pop\|POPTR_0010s24960 | scaffold_10 | 1 | 20889101 | 20888604 | I | 165 | 68_[GLD]_15_[RING-H2]_[14]_1 |
| | pop\|POPTR_0010s24970 | scaffold_10 | 1 | 20893461 | 20892908 | I | 173 | 19_[34]_27_[GLD]_1_[20]_4_[RING-H2]_29 |
| | pop\|POPTR_0018s10710 | scaffold_18 | -1 | 11597865 | 11597497 | H | 151 | 66_[GLD]_13_[RING-H2]_13 |
| | pop\|POPTR_0018s10720 | scaffold_18 | -1 | 11602712 | 11601926 | H | 170 | 97_[RING-H2]_26 |
| | pop\|POPTR_0019s02610 | scaffold_19 | 1 | 2411013 | 2409970 | C | 213 | 18_[71]_18_[4]_10_[GLD]_12_[RING-H2]_[49]_19_[72]_2 |
| | pop\|POPTR_0019s02620 | scaffold_19 | 1 | 2429426 | 2428749 | C | 215 | 17_[71]_24_[4]_19_[GLD]_11_[RING-H2]_[49]_26 |
| *Ricinus communis* | rcu\|30025.t000011 | 30025 | -1 | 108111 | 107509 | B | 200 | 11_[39]_9_[81]_56_[RING-H2]_49 |
| | rcu\|30025.t000012 | 30025 | -1 | 109652 | 109176 | B | 158 | 15_[81]_47_[RING-H2]_33 |
| *Manihot esculenta* | mes\|cassava4.1_026046m.g | scaffold03850 | 1 | 86208 | 85684 | H | 174 | 121_[RING-H2]_6 |
| | mes\|cassava4.1_024474m.g | scaffold03850 | 1 | 92584 | 91521 | H | 172 | 119_[RING-H2]_6 |
| | mes\|cassava4.1_027091m.g | scaffold03850 | 1 | 95563 | 95099 | H | 154 | 10_[7]_75_[RING-H2]_6 |
| | mes\|cassava4.1_033087m.g | scaffold03850 | 1 | 101050 | 100547 | H | 167 | 114_[RING-H2]_6 |
