# Supplementary material for: Diversity in the Architecture of ATLs, a Family of Plant Ubiquitin-Ligases, Leads to Recognition and Targeting of Substrates in Different Cellular Environments
Source: PLoS One. 2011 Aug 24;6(8):e23934. doi: 10.1371/journal.pone.0023934 (PMC3161093; doi:10.1371/journal.pone.0023934)
Supplement: Table S5 — ATL10 interactors mapped to region VII. (PDF) [file pone.0023934.s007.pdf]

Table S5. ATL10 interactors mapped to region VII

AGI*	DESCRIPTION
AT1G05060	Unknown protein
AT1G13635	DNA methyladenine glycosylase
AT1G20100	Unknown protein
AT1G23040	hydroxyproline-rich glycoprotein family protein
AT1G24620	polcalcin, putative / calcium-binding pollen (X2)
AT1G26480	GRF12 (general regulatory factor 12)
AT1G34000	OHP2 (one-helix protein 2)
AT1G71480	NTF2 (nuclear transport factor 2) (X2)
AT1G75950	SKP1A (S phase kinase-associated protein 1)
AT2G14750	APK (APS kinase)
AT2G32670	ATVAMP725 (vesicle-associated membrane protein)
AT2G47340	invertase/pectin methylesterase inhibitor
AT3G19010	oxidoreductase, 2OG-Fe(II) oxygenase
AT3G51895	SULTR3;1 (sulfate transporter 3;1)
AT3G54366	Unknown protein
AT3G54760	dentin sialophosphoprotein-related
AT4G32530	vacuolar ATP synthase, putative (X2)
AT5G06860	PGIP1 (polygalacturonase inhibiting protein) (X2)
AT5G51040	Unknown protein
AT5G54430	PHOS32 (universal stress protein)
AT5G66470	GTP binding / RNA binding protein

* Arabidopsis Gene Index, (X2) indicates that the clone was retrieved two times.
